# Microcolin H, a novel autophagy inducer, exerts potent antitumour activity by targeting PITPα/β

**DOI:** 10.1038/s41392-023-01667-2

**Published:** 2023-11-15

**Authors:** Hange Yang, Xiaowei Zhang, Cong Wang, Hailong Zhang, Juan Yi, Kun Wang, Yanzhe Hou, Peihong Ji, Xiaojie Jin, Chenghao Li, Min Zhang, Shan Huang, Haoyuan Jia, Kuan Hu, Lingyun Mou, Rui Wang

**Affiliations:** 1 Key Laboratory of Preclinical Study for New Drugs of Gansu Province, School of Basic Medical Sciences & Research Unit of Peptide Science, Chinese Academy of Medical Sciences, 2019RU066, Lanzhou University, Lanzhou, Gansu P. R. China; 2 Institute of Materia Medica, Chinese Academy of Medical Sciences, Beijing, P. R. China; 3 Gansu University Key Laboratory for Molecular Medicine & Chinese Medicine Prevention and Treatment of Major Diseases, College of Pharmacy, Gansu University of Chinese Medicine, Lanzhou, China; 4 School of Life Science, Lanzhou University, 730000 Lanzhou, P. R. China

**Keywords:** Drug development, Target identification, Target validation

## Abstract

The identification of effective drug targets and the development of bioactive molecules are areas of high need in cancer therapy. The phosphatidylinositol transfer protein alpha/beta isoform (PITPα/β) has been reported to play an essential role in integrating phosphoinositide trafficking and lipid metabolism in diverse cellular processes but remains unexplored as a potential target for cancer treatment. Herein, data analysis of clinical cancer samples revealed that PITPα/β expression is closely correlated with the poor prognosis. Target identification by chemical proteomic methods revealed that microcolin H, a naturally occurring marine lipopeptide, directly binds PITPα/β and displays antiproliferative activity on different types of tumour cell lines. Furthermore, we identified that microcolin H treatment increased the conversion of LC3I to LC3II, accompanied by a reduction of the level of p62 in cancer cells, leading to autophagic cell death. Moreover, microcolin H showed preeminent antitumour efficacy in nude mouse subcutaneous tumour models with low toxicity. Our discoveries revealed that by targeting PITPα/β, microcolin H induced autophagic cell death in tumours with efficient anti-proliferating activity, which sheds light on PITPα/β as a promising therapeutic target for cancer treatment.

## Introduction

Globally, cancer ranks as a leading cause of death and one of the biggest barriers to increasing life expectancy in decades. In the year 2020 alone, there were a staggering 19.3 million new cases of cancer reported, resulting in almost 10 million deaths.^1^ Throughout history, natural products have emerged as invaluable resources in the development of drugs to combat various human disorders,^2^ with a particular focus on anticancer chemotherapeutics.^3^ Their contributions have been substantial and continue to hold great promise in the fight against cancer. Microcolins and majusculamide D are a series of compounds with similar structures isolated from the marine cyanobacterium *Moorea producens* (formerly *Lyngbya majuscula*) (Supplementary Fig. S1). Since 1988, microcolin A-D and majusculamide D have been isolated, and subsequent biological studies have shown that these compounds exhibited broad antitumour activities against different types of human cancer cells in vitro.^4–9^ In 2019, nine new microcolin E-M were isolated and showed significant cytotoxic activity against lung cancer cells.^10^ Therefore, previous investigations suggest the potential possibility of microcolins in exploring anticancer strategy.

Autophagy is a fundamental physiological process that plays a crucial role in maintaining cellular homoeostasis. It involves the degradation and recycling of cellular components, such as proteins and damaged organelles, through a process of self-digestion. This process is conserved across all eukaryotic cells and serves as a vital mechanism for the generation of new cells. By recycling intracellular components and metabolites and removing protein aggregates, defective organelles, and intracellular pathogens autophagy participates in the regulation of cell metabolism and energy control and is closely linked to both cellular protection and cell death. A variety of stressful conditions can upregulate autophagy flux, such as nutritional deprivation, metabolic, oxidatives, pathogens, DNA damages, and proteotoxic cues.^11^ Autophagy plays a crucial role in the pathogenesis and progression of various metabolic diseases. This highly dynamic process is intrinsically linked to the regulation of immunity, neurodegenerative disorders, heart disease, and particularly cancer.^12^ In cancer, many researches showed the dual functions of autophagy on the different stages of tumourigenesis: autophagy activation can promote cancer cell survival (protective autophagy) in tumour progression or contribute to cancer cell death (cytotoxic/nonprotective autophagy) of initial oncogenesis.^13,14^ Undoubtedly, cytotoxic autophagy holds immense potential in the field of anticancer research. Substantial evidence supports the notion that enhancing autophagy may serve as a preventive measure against cancer development, particularly in premalignant lesions.^11^ Several approved drugs, experimental agents and natural compounds (such as metformin, curcumin, quercetin, and gemcitabine) induce autophagy in different types of cancer.^15^ Other autophagy inducers (such as obatoclax, vitamin D analogue, and rapamycin) increase cancer cell death when combined with radiation and chemotherapy.^16^ Consequently, activating autophagy may serve as an effective strategy, and there is an urgent need to develop novel and more effective autophagy inducers for cancer treatment.

Phosphatidylinositol transfer protein (PITP), an abundant and ubiquitous soluble protein, is widely distributed in eukaryotes.^17–20^ Mammalian PITPα and β bind and transfer phosphatidylinositol (PI) and phosphatidylcholine (PC) from the endoplasmic reticulum (ER) to various cellular membranes.^21^ Accumulating evidence indicates that PITPs were the potential therapeutic targets for a wide range of diseases, such as cancer,^22^ neurodegenerative disease,^23^ Duchenne muscular dystrophy,^24^ inflammation, and angiocardiopathy.^25^

Here, we identified that patients with gastric cancer with high PITPα/β expression have shorter overall survival, which implicates PITPα/β as potential targets for cancer treatment. Moreover, we first report that microcolin H directly targets PITPα/β and has shown good anticancer activity in vitro and in vivo by activating autophagy.

## Results

### PITPα/β were highly expressed in tumour tissues and were associated with a poor prognosis in gastric cancer

**Fig. 1:**
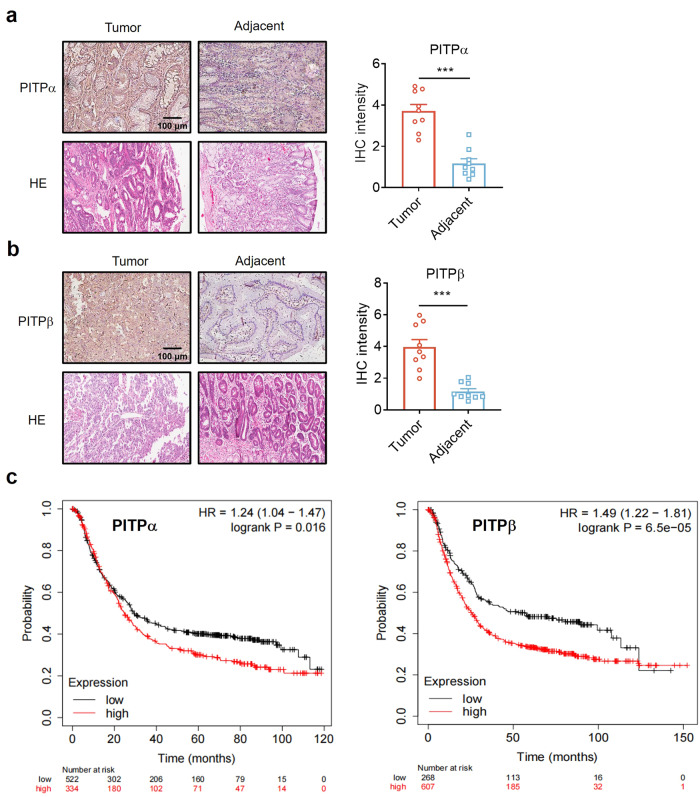
PITPα/β were highly expressed in tumour tissues and were associated with a poor prognosis in gastric cancer. **a**, **b** PITPα/β expression in gastric cancer patients revealed by IHC staining. Representative images from a tissue microarray are shown. Original magnification, ×10; scale bar: 100 μm, *n* = 9 per group. **c** Kaplan‒Meier analysis (http://kmplot.com) of overall survival defined by PITPα/β expression in gastric cancer patients. Data are presented as the mean ± SEM. *P < 0.05, **P < 0.01, ***P < 0.001

To illustrate the potential relationship between the expression of PITPα/β and cancer, immunohistochemistry (IHC) assays were performed in a tissue microarray (TMA) containing adjacent- and tumour-human gastric cancer samples. PITPα/β expression in tumour tissues was significantly higher than that in adjacent tissues (Fig. 1a, b). The relationship between PITPα/β expression and gastric cancer prognosis was evaluated by using Kaplan‒Meier survival analysis (http://kmplot.com). A total of 875 gastric cancer patients were divided into two groups by the best cutoff value of PITPα (PITPα^high^ = 334, PITPα^low^ = 552) and PITPβ (PITPβ^high^ = 607, PITPβ^low^ = 268). High PITPα expression was accompanied by a poor prognosis, with members of the PITPα^high^ group having an overall survival (OS) time of 24.8 months, compared with an OS time of 29.4 months in the PITPα^low^ group (*p*-value = 0.016). Similarly, the PITPβ^high^ group showed a poorer prognosis, with an overall survival of 25.5 months, than the PITPβ^low^ group (57.13 months, *p* = 6.5e-5) (Fig. 1c). First progression survival (FPS) and post-progression survival (PPS) analyses showed that lower PITPα/β expression came with the benefit of delayed disease progression (Supplementary Fig. S2). These results showed that PITPα/β expression was increased in gastric cancer patients and was related to poor prognosis.

### Microcolin H was synthesised with a 200 mg scale

To facilitate our subsequent chemical proteomic and biological research, it is necessary to develop a chemical method for the synthesis of microcolins. Microcolin H, a member of microcolins, bear only one chiral centre on the aliphatic carboxylic acid fragment, which provides a convenient for large-scale synthesis. We split microcolin H into three fragments, namely, aliphatic carboxylic acid (M1), tripeptide (M2), and pyrrolinone (M3), which converge at a late stage via standard peptide coupling reactions.

For the synthesis of M1, we chose (R)-4-benzyloxazolidin-2-one as the starting material, which, after the two-step reaction (n-BuLi, octanoyl chloride; NaNSi_2_Me_6_, MeI, H_2_O_2_), resulted in the final product. To efficiently build the tripeptide backbone of M2, valine derivative 3 was subsequently conjugated with commercially available Boc-threonine 4 in the presence of Bop-Cl/Et_3_N, followed by acetylation (Ac_2_O/DMAP) to produce known dipeptide 6. Then, tripeptide M2 was accessed via Boc deprotection of 6 followed by coupling with commercially available N-methyl Boc-leucine 7, and subsequently, the benzyl group was removed by hydrogenation. Finally, for the pyrrolinone fragment of M3, we followed the protocol reported by Gerwick et al.^5^ Beginning with commercially available N-Boc-cis-4-hydroxy-L-proline, the alcohol was protected by tert-butyldimethylsilyl (TBS) and the acid was coupled with pentafluorophenol to produce 10, which was reacted with chiral pyrrolidone under BuLi conditions to provide imide 11. Then, following the two-step sequence (LiHDMS, PhSeBr, H_2_O_2_; 4 N HCl in dioxane), pyrrolinone M3 was synthesised from imide 11.

**Fig. 2:**
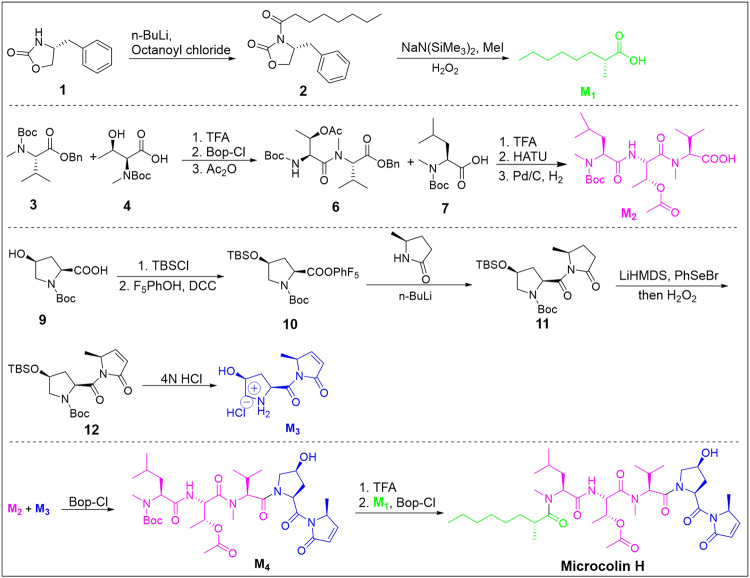
Synthesis of microcolin H

To complete the synthesis of microcolin H, the tripeptide carboxylic acid M2 was coupled with M3 using Bop-Cl to produce M4, and N-Boc was removed by using trifluoroacetic acid (TFA), which was then conjugated with octanoic acid M1 under BOP-Cl/Et3N conditions to yield microcolin H with a production of approximately 200 mg (Fig. 2). Passingly, we synthesised five analogues with different lengths of aliphatic carboxylic acids to elucidate the structure-activity relationships (SARs) of the lipid chain (Fig. 4a and Supplementary Fig. S3a).

### Microcolin H inhibits cancer cell proliferation and migration

**Fig. 3:**
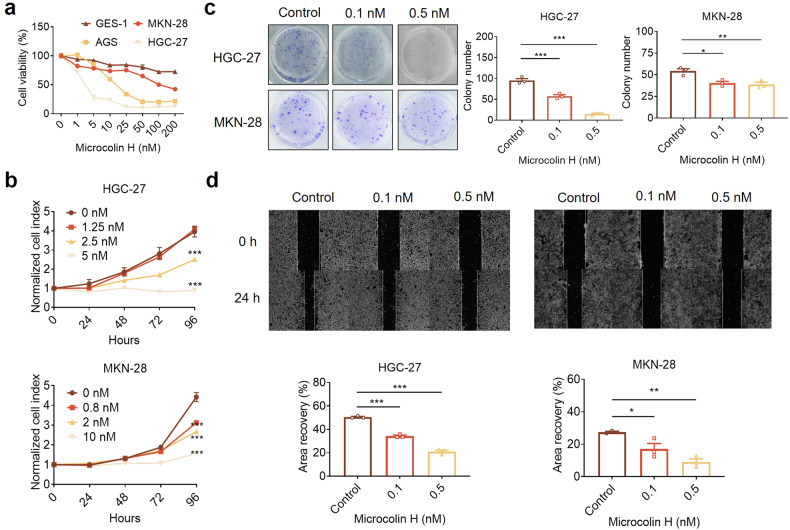
Microcolin H inhibited the proliferation and migration of gastric cancer (GC) cells. **a** Cell Counting Kit-8 (CCK-8) assay was used to analyse the effect of microcolin H on GC cell growth. Cells were treated with various doses of microcolin H for 24 h, *n* = 4 per group. **b** GC cells were treated with various concentrations of microcolin H, and cell proliferation was detected by growth curve analysis, *n* = 4 per group. **c** HGC-27 and MKN-28 cells were treated with various concentrations of microcolin H for 48 h. Then, the cells were cultured for 9 days, and the colonies were counted by ImageJ, *n* = 3 per group. **d** Wound healing assays were used to measure HGC-27 and MKN-28 cell migration following treatment with microcolin H at the indicated concentrations for 24 h, *n* = 3 per group. The results presented here are representative of three independent experiments. Data are presented as the mean ± SEM. *P < 0.05, **P < 0.01, ***P < 0.001, vs. control group

After synthesising microcolin H, we confirmed its role in more extensive cell line growth. All of the cells were treated with microcolin H for 48 h, and cell viability was detected using a CCK-8 assay. We observed that microcolin H showed significant cytotoxicity irrespective of cancer type (Supplementary Fig. S3b). We found that microcolin H showed significant antiproliferative effects in gastric cancer cell lines (HGC27, AGS, and MKN-28) dose-dependently but not in gastric mucosa epithelial cell line GES-1 (Fig. 3a). Microcolin H attenuated the growth curve of the gastric cancer cell lines and distinctively reduced the colony formation ability (Fig. 3b, c). We also asked whether microcolin H would inhibit the migration of gastric cancer cells. With microcolin H treatment for 24 h, cell migration was significantly inhibited compared to that in the control group (Fig. 3d). Altogether, these results indicate that microcolin H exhibited strong antitumour activity in vitro.

### Microcolin H directly targets PITPα and PITPβ

**Fig. 4:**
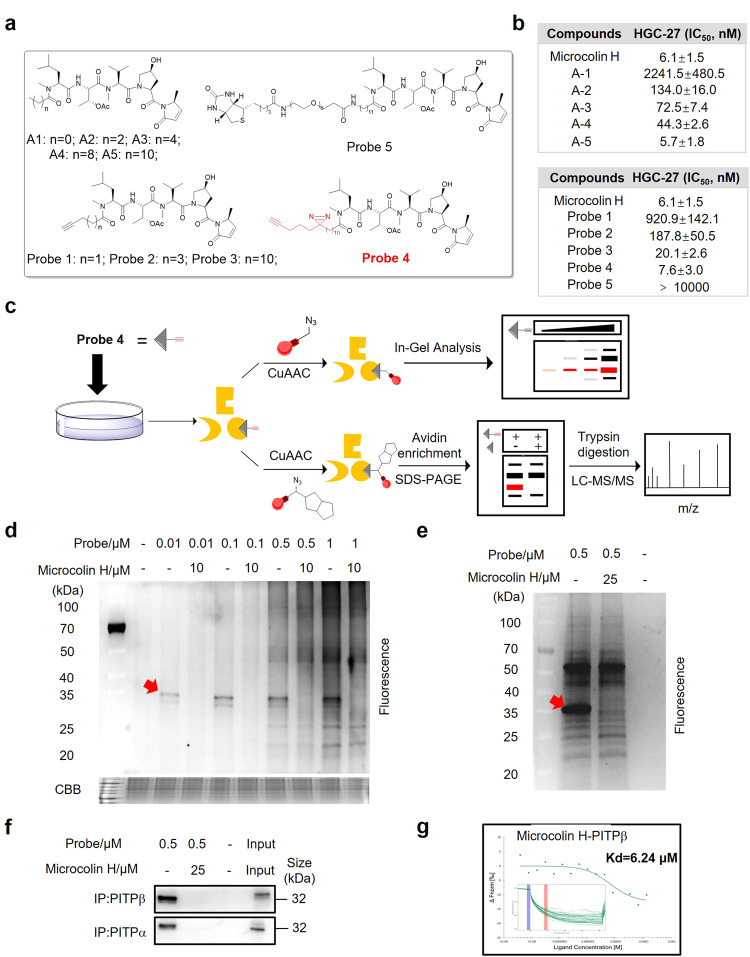
Identification of PITPα/β as the target of microcolin H. **a** Structures of analogues 1-5 and probes 1-5. **b** A cell counting kit-8 (CCK-8) assay was used to analyse the effect of analogues and probes on HGC-27 cell viability. Cells were treated with various doses of probes for 48. **c** Labelling and pull-down workflow. **d** Competitive Labelling with microcolin H in HGC-27 cells. The complete gel of Labelling was stained with Coomassie brilliant blue (CBB). **e** Pull down of HGC-27 cells, CuAAC with “TAMRA-Bioin-Azide” trifunctional reporter, followed by enrichment and in-gel analysis. **f** Western blot analysis to determine the competitive binding of the probe and microcolin H to PITPα and PITPβ. **g** MST analysis was used to assess the binding between microcolin H and PITPβ in kinetic level. Data are presented as the mean ± SD

Activity-based protein profiling (ABPP) is an elegant method that combines proteomics techniques with activity-based probes to identify protein targets of bioactive small molecules to help understand their mechanisms of action (MOA).^26,27^ Therefore, to comprehensively evaluate the genuine target of microcolin H, we designed and synthesised a set of activity-based probes **1**–**5** according to the structure-activity relationship of analogues A1-A5 (Fig. 4a). Notably, the capacity of probe **4** to inhibit cancer cell proliferation was close to that of parental microcolin H. The remaining probes 1–3 had varying degrees of proliferation loss similar to the analogues. In particular, biotinylated probe **5** completely lost its activity (Fig. 4b). These results indicated that the length of the aliphatic carboxylic acid significantly regulated the activity.

Based on the experimental results of the antitumour activity, we chose probe **4** to examine potential protein targets of microcolin H in cancer cells using two chemical proteomic approaches (Fig. 4c). First, the HGC-27 cell lysates of the competition groups were treated with 10 μM microcolin H for 0.5 h, and all the groups were treated with different concentrations of probe **4** for an additional hour, followed by a click reaction with rhodamine-azide. Following SDS‒PAGE and in-gel fluorescence scanning of the labelled proteomes, probe 4 produced a highly selectively labelled band at 35 kDa even at a concentration of 0.01 μmol/L; with increasing probe concentrations (>0.5 μmol/L), other labelled bands appeared, indicating off-targeting (Fig. 4d). It is worth mentioning that the photoaffinity group “diazirine” irradiated with UV light did not affect the labelling result, which reminds us that microcolin H is covalently bonded with its target proteins (Supplementary Fig. S4a). This ~35 kDa probe-labelled band could be successfully blocked by preincubation with microcolin H, indicating that the ~35 kDa band likely represents the true targets of microcolin H.

Subsequently, another chemical proteomic approach, “pull down”, was employed to identify the target protein. We treated the probe **4**–labelled proteome with a “biotin-azide-rhodamine” trifunctional reporter via CuAAC. Following the pull-down with avidin beads, the enriched proteome was separated by SDS‒PAGE. After enrichment, the ~35 kDa probe-labelled band was very evident in the fluorescence scanning (Fig. 4e). Then, the ~35 kDa labelled band was cut off from the gel for the subsequent gel digestion and LC-MS/MS analysis. LC‒MS/MS analysis revealed that phosphatidylinositol transfer protein alpha/beta isoform (PITPα/β) might be a target of microcolin H (Supplementary Figs. S4b, c and S5). Furthermore, Western blot analysis confirmed that PITPα/β could be selectively recovered by probe **4** (Fig. 4f and Supplementary Fig. S4d).

To further detect the binding character of microcolin H and PITPα/β at the protein level, we carried out thermodynamic and kinetic experiments. The thermal shift assay was used to evaluate the thermal stabilisation of microcolin H and PITPα/β. The result showed that after microcolin H bound with PITPα/β, the thermal stabilisation of PITPα/β was increased obviously compared with the control group (Supplementary Fig. S4e). Microscale thermophoresis (MST) was performed to detect the microcolin H-PITPα/β kinetic, which showed a strong binding between microcolin H and PITPα/β with affinity (KD) value of about 6.2 μM (Fig. 4g). Furthermore, docking simulation predicted that Cys94 is the most possible binding site of covalent engagement using the reported structure of PITPα/β (Supplementary Fig. S4f). On the basis of the above results, we believe that PITPα/β are the direct molecular targets of microcolin H.

**Fig. 5:**
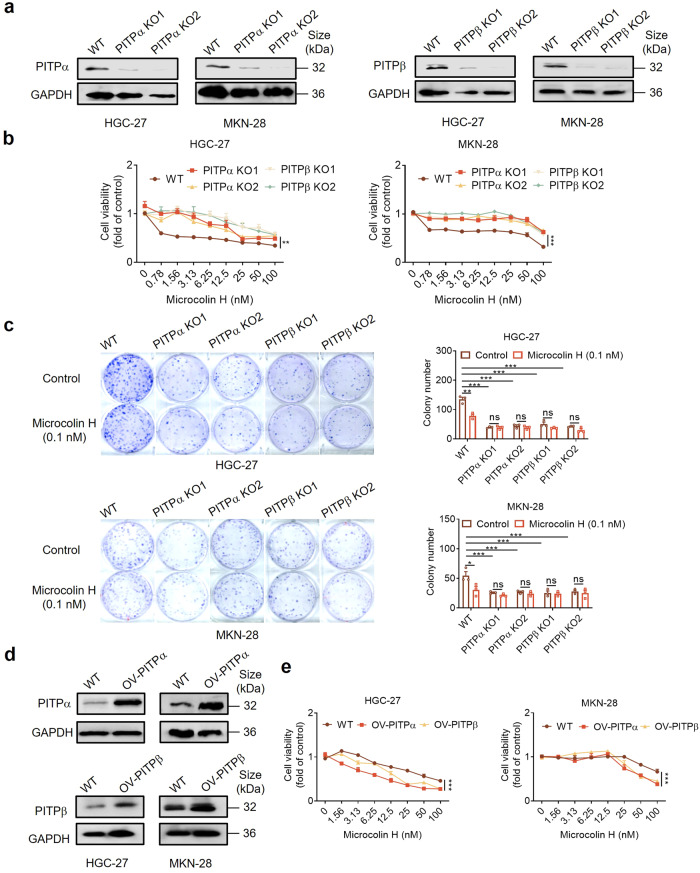
Microcolin H functions by binding PITPα/β. **a** Western blot analysis of PITPα/β expression in wild type (WT) and PITPα/β knockout HGC-27/MKN-28 cells (PITPα/βKO-1/2). **b** Wild type (WT) and PITPα/β knockout HGC-27/MKN-28 cells (PITPα/βKO-1/2) were treated with various concentrations of microcolin H for 12 h, and cell proliferation was detected by using a CCK-8 assay, *n* = 3 per group. **c** Wild type (WT) and PITPα/β knockout HGC-27/MKN-28 cells (PITPα/βKO-1/2) were treated with microcolin H for 48 h. Then, the cells were cultured for 9 d, and the colonies were counted, *n* = 3 per group. **d** Western blot analysis of PITPα/β expressions for wild type (WT) and PITPα/β overexpression HGC-27/MKN-28 cells (OV-PITPα/β). **e** Wild type (WT) and PITPα/β overexpression HGC-27/MKN-28 cells (OV-PITPα/β) were treated with various concentrations of microcolin H for 12 h, and cell proliferation was detected by using a CCK-8 assay, *n* = 3 per group. Data are presented as the mean ± SEM. *P < 0.05, **P < 0.01, ***P < 0.001, vs. control group

To determine whether microcolin H achieves pharmacological functions by acting on target proteins, we generated PITPα/β knockout cells (MKN-28 and HGC-27) using the CRISPR/Cas system vector pSpCas(BB)-2A-Puro(PX459)V2.0. PITPα/β expression was suppressed after gastric cancer cells were immediately transfected with indicated plasmids (vector control, PITPα KO1/KO2; plasmids PITPβ KO1/KO2) (Fig. 5a). Compared with the control group, the cells transfected with either PITPα KO plasmids or PITPβ KO plasmids showed less sensitivity in response to microcolin H treatment (Fig. 5b). In consistence, colony formation was reduced in both PITPα and PITPβ knockout cells, and microcolin H could not further inhibit the colony formation in the PITPα/β knockout cell lines (Fig. 5c). In contrast, when PITPα or PITPβ was overexpressed in GC cells (OV-PITPα/β), they exhibited increased cell growth abilities and enhanced sensitivity to microcolin H treatment (Fig. 5d, e and Supplementary Fig. S4g).

Thus, these results confirmed that PITPα/β expression was necessary for tumour activity and is indeed the genuine target of microcolin H under biologically relevant conditions.

### Microcolin H induces autophagy-dependent cell death via PITPα/β

**Fig. 6:**
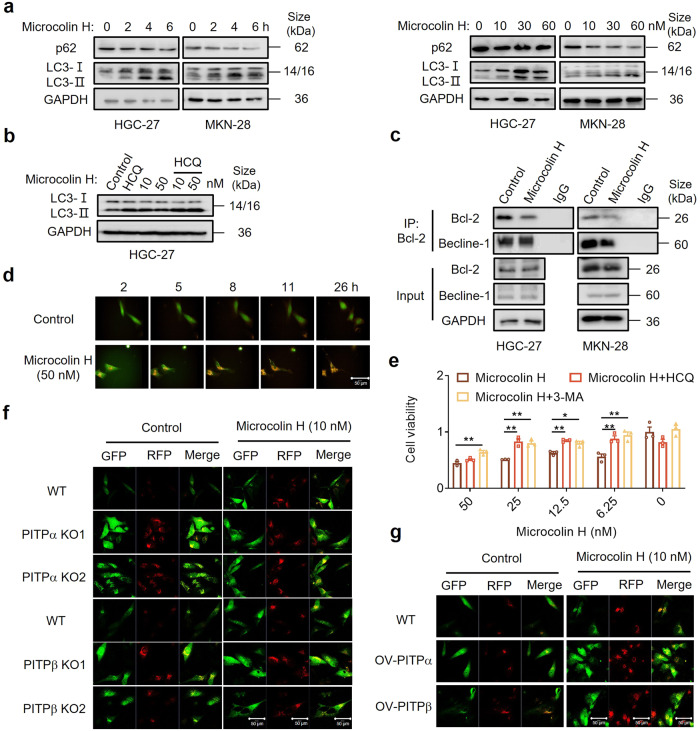
Microcolin H induces autophagy by binding PITPα/β. **a** The HGC-27/MKN-28 cells were incubated with microcolin H as indicated time or concentration. Whole lysate was used for detecting p62 and LC3 expression using western blot assay. **b** Western blot analysis of LC3 expression after HGC-27 cells had been treated with microcolin H and HCQ. **c** Co-IP assay with an antibody against BCL-2 to detect the BCL-2/Beclin-1 interaction in gastric cancer cells treated with microcolin H for 6 h. **d** HGC-27 cells were treated with 50 nM microcolin H or DMSO and dynamically monitored by a content analyser for 26 h. **e** A cell counting kit-8 (CCK-8) assay was used to analyse the effect of microcolin H on HCQ/3-MA (25 μM) pre-treated HGC-27 cells viability. Cells were treated with various doses of microcolin H for 7 h, *n* = 3 per group. **f**, **g** HeLa-Difluo™ hLC3 cells and transfected HeLa-Difluo™ hLC3 cells were treated with microcolin H, and autophagosomes were observed by CLSM. Data are presented as the mean ± SEM. *P < 0.05, **P < 0.01, ***P < 0.001, vs. control group

Given the unclear role of PITPα/β in gastric cancer, we then investigated how microcolin H inhibits cell proliferation via PITPα/β. Since microcolin H significantly reduced gastric cancer cell proliferation, we investigated the possible mechanisms of microcolin H-mediated cell death. Both Western blot analysis and flow cytometry of Annexin V/PI staining demonstrated that hardly any cells treated with microcolin H were undergoing apoptosis (Supplementary Fig. S6a, b). Since PITPα/β were reported to be essential interfaces between lipid metabolism and membrane trafficking from the trans-Golgi network,^28,29^ and autophagy is involved in the membrane-trafficking pathway leading to eukaryotic cells recycling or degrading internal constituents.^30,31^ To examine whether microcolin H-induced cell death was associated with autophagic death, we first detected the effect of microcolin H on the autophagy process in gastric cells. Microcolin H increased the conversion of LC3I to LC3II, accompanied by reduction of the level of p62, both serving as classic biomarkers of the autophagy process (Fig. 6a).^32,33^ To distinguish whether the alteration of the markers was caused by induction or inhibition of autophagy, we used an autophagy inhibitor hydroxychloroquine (HCQ, 25 µM) to pre-treat the cells for 2 h followed by the administration of microcolin H for 6 h. It was shown that compared with control, HCQ caused more conversion of LC3I to LC3II in microcolin H-treated cells while the degradation of p62 was inhibited, which suggested that microcolin H activated autophagy (Fig. 6b and Supplementary Fig. S6c). In addition, the anti-apoptotic protein Bcl-2 can interact with the autophagy protein beclin-1 and the disruption of the interaction between Bcl-2 and beclin-1 contributes to autophagy.^34–36^ The co-immunoprecipitation assay showed that when gastric cancer cells were exposed to microcolin H for 6 h, the complex of beclin-1 and Bcl-2 significantly reduced (Fig. 6c). To further confirm, we added microcolin H in an established HeLa cell line transfected with GFP-tagged LC3 plasmid.^37^ Confocal microscopy showed that microcolin H caused a significant accumulation of GFP-LC3 puncta, which represents the increase of autophagy activity in HeLa-GFP-LC3 cells in a dose-dependent manner (Supplementary Fig. S6d). Autophagic flux was detected by the HeLa-Difluo™ hLC3 cells express a fusion protein RFP::GFP::LC3, in which the N-terminus of human LC3B is fused to two fluorescent reporter proteins: an RFP (acid-stable) and a GFP (acid-sensitive). Early in autophagy, both RFP and GFP signals are detected. As the fusion of the autophagosomes with the lysosomes progresses, the GFP fluorescence diminishes, leaving only the RFP fluorescence visible. Both dual fluorescent red and green RFP::GFP::LC3 puncta or single fluorescent red RFP::LC3 puncta have not been observed in the control group (Fig. 6d and Movie 1). The distribution of the fluorescent reporter proteins was markedly altered in Microcolin H-treated cells, in which we observed more red and green RFP::GFP::LC3 puncta at the early autophagy and leaving only the RFP fluorescence visible during the fusion of the autophagosomes with the lysosomes progresses (Fig. 6d and Movie 2).

To determine whether microcolin H caused autophagic cell death, we treated the gastric cancer cells with microcolin H at different concentrations in the presence or absence of the autophagy inhibitor HCQ or 3-MA (3-methyladenine). As shown in Fig. 6e, either HCQ or 3-MA reversed the antiproliferative effect of microcolin H in HGC-27 cells, which implied that microcolin H-induced cell death was autophagy-dependent.

Then we investigated to confirm the role of PITPα/β in microcolin H-induced autophagic cell death. The ectopic PITPα/β expression was established in gastric cancer cell lines MKN-28 and HGC-27, which suppressed autophagy as featured by the reduced LC3II accumulation, as well as the minor effect of p62 degradation (Supplementary Fig. S6e). In comparison, PITPα/β knockout stimulated autophagic flux in gastric cancer cell MKN-28 and HGC-27 cells (Supplementary Fig. S6f). Consistently, the cells overexpressing PITPα/β increased the amount of beclin-1/Bcl-2 complex (Supplementary Fig. S6g). Since microcolin H directly targeted PITPα/β, we wondered if PITPα/β were involved in the microcolin H-induced autophagy process and cell death. We transfected HeLa-GFP-LC3 cells with specific dual gRNA of PITPα/β (Supplementary Fig. S6h). As shown in Supplementary Fig. S6i, PITPα/β knockout significantly increased autophagy activity, while microcolin H treatment could not further increase the autophagosomes in PITPα/β knockout cells. On the other hand, overexpression of PITPα/β improved the sensitivities of gastric cancer cells to microcolin H, accompanied by augmented autophagosome formation. PITPα/β-KO HeLa-Difluo™ hLC3 cells were visualised to characterise the influence of microcolin H on PITPα/β-mediated autophagy. As shown in Fig. 6f, RFP:GFP::LC3 puncta increased in PITPα/β-KO cells and Microcolin H could not further stimulate the autophagosome formation. We also transfected HeLa-Difluo™ hLC3 cells with OV-PITPα/β plasmids, in which microcolin H treatment induced the additional increase of RFP:GFP::LC3 puncta compared with untransfected cells (Fig. 6g and Supplementary Fig. S6j). HGC-27 and MKN-28 cells overexpressing PITPα/β exhibited a reduced protein level of LC3II and after microcolin H treatment caused more conversion of LC3I to LC3II in HGC-27-OV than in parental cells (Supplementary Fig. S6k, m). In contrast, the autophagic flux level was elevated in PITPα/β-KO cells, in which microcolin H could not further increase the autophagy level (Supplementary Fig. S6l, n). OV-PITPα/β cells with microcolin H treatment for 48 h showed further cell death compared that in WT cells; however, PITPα/β-KO cells had little difference on cell death in the presence or absence of microcolin H (Supplementary Fig. S6o, p). Taken together, these results supported that microcolin H was an efficient autophagy inducer in gastric cancer cells and PITPα/β played an important role in the microcolin H-induced autophagy process. Although there might be other possible mechanisms to be unveiled, microcolin H-induced cell death was autophagy-dependent.

### Microcolin H has strong antitumour activity in vivo and is well tolerated in mice

**Fig. 7:**
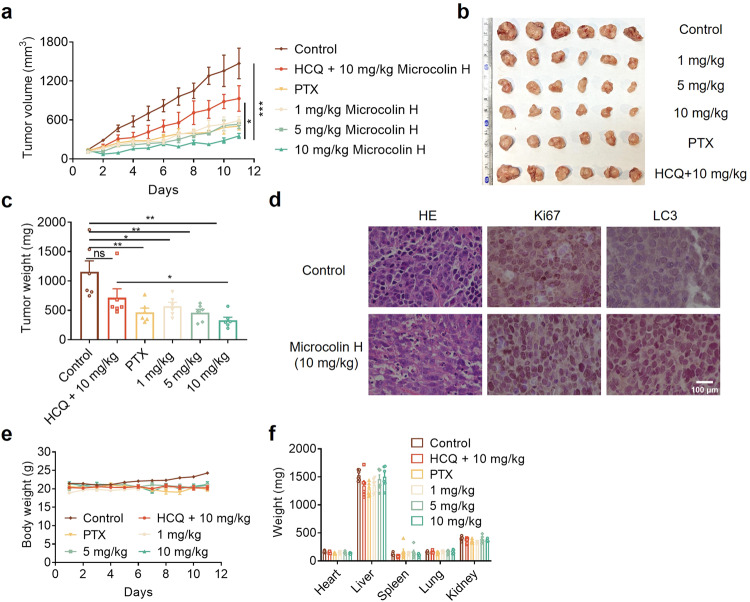
Evaluation of the antitumour efficacy of microcolin H. **a** Curves of the average tumour volume in the six groups following the day of microcolin H administration, *n* = 6 per group. **b** Images of tumours dissected from each mouse in different groups on the day the mice were sacrificed, *n* = 6 per group. **c** The tumour weight from each mouse in different groups on the day the mice were sacrificed, *n* = 6 per group. **d** Mouse tumours were stained for HE, Ki67 and LC3. **e** Curves of average mouse weight in the six groups following the day of microcolin H administration, *n* = 6 per group. **f** Curves of average organ weight in the six groups on the day the mice were sacrificed, *n* = 6 per group. Data are presented as the mean ± SEM. *P < 0.05, **P < 0.01, ***P < 0.001, vs. control group

Encouraged by the above results, we further evaluated the antitumour effect of microcolin H in vivo using the HGC-27 cell-derived xenograft tumour model. We randomly arranged Balb/c-nu/nu mice with a tumour volume of at least 150 mm^3^ into six groups. The mice were intraperitoneally injected with microcolin H at the dosages of 1 mg/kg, 5 mg/kg, and 10 mg/kg respectively. Paclitaxel (PTX, 8 mg/kg) was used as a positive control, and the negative control group mice were injected with PBS. As shown in Fig. 7a–c, microcolin H remarkably reduced the tumour size and weight in a dose-dependent way after 11 days of treatment. The 10 mg/kg microcolin H-treated group showed a TGI value of 74.2%, better than the TGI of the PTX positive control. Importantly, when HCQ was used in combination with microcolin H (50 mg/kg HCQ + 10 mg/kg microcolin H), the growth-inhibitory effect of the compound was significantly alleviated. IHC staining showed that Ki67, as a proliferative marker, decreased in the xenograft tumours treated with microcolin H while the autophagy indicator LC3 increased (Fig. 7d). These results suggested that microcolin H inhibited the growth of gastric cancer cells and induced their autophagy process in vivo, which was consistent with the conclusions of in vitro studies (Supplementary Fig. S7a).

Furthermore, the safety of microcolin H treatment was evaluated in mouse models. It is worth mentioning that mouse body weight did not significantly change during microcolin H treatment (Fig. 7e), indicating that microcolin H has no potent toxicity at the indicated dosages in vivo. At the end of the mouse experiment, the pathological sections of organs and the blood biochemical markers analysis were further used to evaluate the toxicity in vivo. H&E staining reminded that there were no obvious pathological changes in the studied organs (hearts, livers, spleens, lungs, and kidneys) between the microcolin H-treated groups and the control group (Supplementary Fig. S7b). In addition, the weight and appearance of mouse organs showed no obvious damage between the control group and the microcolin H-treated groups (Fig. 7f and Supplementary Fig. S7c). Moreover, there were no apparent changes in the blood biochemical markers after microcolin H treatment (Supplementary Fig. S7d). These experiments indicated that microcolin H metabolism was innocuous and was well tolerated in vivo. All of these data identified microcolin H as an effective small molecule with low toxicity that can remarkably suppress tumour growth in vivo.

## Discussion

The pursuit of discovering and validating new drug targets for cancer treatment is not only crucial but also incredibly exciting. The identification of novel targets holds the potential to revolutionise cancer therapy and improve patient outcomes. Kaplan‒Meier survival analysis revealed that a low level of PITPα/β expression significantly improves the lifetime of overall survival and disease progression in gastric cancer. We have also discovered that in clinical cancer samples, PITPα/β upregulation has been confirmed in tumour tissue compared to adjacent nontumour tissue. It should be mentioned that while we collected data and prepared our manuscript, Guan et al. reported microcolin B/VT01454 as an inhibitor targeting PITPα/β to activate the Hippo/YAP pathway and suppress the proliferation of YAP-dependent tumour cells.^38,39^ It was more distinct to reveal that PITPα/β are potential targets for cancer therapy.

Microcolins are a family of compounds with extensive cytotoxicity from the marine cyanobacterium *Moorea producens*. We accomplished the total synthesis of microcolin H with a significant scale (>200 mg), which was the basis for our subsequent experiments (Fig. 2). Preliminary in vitro tests indicated that microcolin H inhibited cell proliferation and had outstanding anticancer activity in pancancer cell lines, especially in gastric cancer lines (MKN-28, HGC-27, AGS), while it showed low toxicity in normal epithelial cells (GES-1) (Fig. 3).

Natural products exert their pharmacological activity through interactions with intracellular protein targets. In order to discover and develop new natural medicines, target identification is the initial key step.^40^ We employed two chemical proteomic approaches to prove that PITPα/β are direct targets of microcolin H in mammalian cells with a chemical probe based on microcolin H. The thermal shift assay experiment, MST analysis, and docking simulations further validated the results (Fig. 4). Knockout of PITPα/β not only inhibited cell growth but also decreased the sensitivity of gastric cancer cells to microcolin H. Similarly, overexpressing PITPα/β increases tumour cell survival and response to microcolin H treatment. These findings suggest that microcolin H could selectively bind PITPα/β, resulting in the subsequent potent inhibition of tumour cell proliferation (Fig. 5).

Autophagy modulation has been suggested as a potential cancer therapeutic strategy, and we further explored the role of microcolin H in autophagy. First, we examined the effect of microcolin H on the autophagy in cells pre-treated with autophagy inhibitor HCQ. The results showed that while the fusion of autophagosomes and lysosomes was repressed, microcolin H stimulated the early autophagy step (Fig. 6b). Second, the Co-IP assay showed that microcolin H released freer Beclin-1 from blocking the interaction of Beclin-1 and Bcl-2 to prevent the degradation of Beclin-1 (Fig. 6c). Meanwhile, HCQ and 3-MA reduced the antitumour effect of microcolin H (Fig. 6e). Furthermore, the activity of autophagy was increased in the HeLa-GFP-LC3 cell and HeLa-Difluo™ hLC3 cells model, which knocked out the PITPα/β proteins, while the conversion of LC3I to LC3II increased and p62 was reduced, supporting the hypothesis that the autophagic flux was influenced by PITPα/β expression. Microcolin H was identified to increase the number of LC3 puncta in HeLa-GFP-LC3 or HeLa-Difluo™ hLC3 cells with basic PITPα/β expression. Overexpression of PITPα/β enhanced the response of microcolin H to affect autophagosome formation. Inhibition of PITPα/β expression blocked the response to microcolin H treatment, leading to no significant difference in the number of LC3 puncta. We also confirmed the effect of microcolin H-induced autophagy by targeting PITPα/β in MKN28 and HGC27 cells. On account of the similar structure between microcolin H and VT01454 (researched by Guan et.al), we are all very curious whether VT01454 will cause autophagy in cancer cells. We synthesised VT01454 and found that VT01454 also induced autophagy similar to microcolin H (Supplementary Fig. S7e). Based on the above results, the role of microcolin H in autophagy is worth exploring in detail to demonstrate the anticancer activity of microcolin H, especially in regard to the relationship between PITPα/β-mediated membrane transportation and tumour autophagy.

Finally, we evaluated the antitumour effects of microcolin H in vivo. After 11 days of consecutive administration, tumour growth was remarkably suppressed, and all the microcolin H treatment groups showed almost no symptoms associated with toxicity. Meanwhile, the HCQ-microcolin H combination treatment reduced the antitumour effect of microcolin H in vivo, which implied that microcolin H slowed down the tumour growth and was autophagy-dependent. Expression of the proliferative marker Ki67 significantly decreased with microcolin H treatment, while the autophagy indicator LC3 was increased. Thus, our results demonstrate that PITPα/β are very promising tumour therapeutic targets and that the marine lipopeptide microcolin H, as a novel autophagy inducer, shows potential antitumour activity by targeting PITPα/β. In addition to cancer, microcolin H and its analogues might also have the potential to treat PITPα/β-driven diseases such as neurodegeneration, immunity and metabolic diseases. On the other hand, the PITP family are involved in the transfer of insoluble phosphatidylinositol among the different organelle membranes, which provides the possibility for the regulation of lipid metabolism by microcolin H.

## Materials and methods

### Cell culture and cell lines

For proteomics and biological experiments, HuH-7, HepaRG, PANC-1, A549, H460, Hela, HGC-27, AGS, MKN-28, and GES-1 cells were acquired from the National Collection of Authenticated Cell Cultures (Shanghai, China). HeLa-Difluo™ hLC3 cells were acquired from InvivoGen. HuH-7, HepaRG, PANC-1, A549, H460, Hela, HeLa-Difluo™ hLC3, HGC-27, MKN-28 and GES-1 cell lines were cultured in DMEM media (BasalMedia Technologies Co., LTD. Shanghai, China) supplemented with 10% (v/v) fetal bovine serum (FBS; ABW, NOVA Medical Science And Technology Co., LTD. Shanghai, China) and 1% (v/v) penicillin-streptomycin solution (Labgic Technology Co., LTD. Beijing, China). AGS cell line was cultured in RPMI-1640 media (BasalMedia Technologies Co., LTD. Shanghai, China) supplemented with 10% (v/v) fetal bovine serum (FBS; ABW, NOVA Medical Science And Technology Co., LTD. Shanghai, China) and 1% (v/v) penicillin-streptomycin solution (Labgic Technology Co., LTD. Beijing, China). The cells were grown in a humidified incubator with 5% CO_2_ at 37 °C. All cells were routinely tested to ensure there were no contaminations of mycoplasma.

### Cell viability assay

Cell counting kit-8 (CCK-8) assay (New Cell & Molecular Biotech Co., LTD. Suzhou, China) was used to detect the effect of microcolin H on cell viability. Briefly, 5 × 10^3^ cells were seeded into 96-well plates. Next, the cells were treated with the indicated concentrations of microcolin H for 48 h, followed by incubating of the cells with 10 μL CCK-8 reagent for 1 h. The absorbance (OD) was measured at 450 nm using Flex Station III Services (Thermo Fisher Scientific, Waltham, MA, USA). Trypan blue dye staining was performed to assess the effects of PITPα/β KO GC cells with/without microcolin H on cell survival. Briefly, 1 × 10^5^ cells treated with the indicated were resuspended in 1 ml of DMEM medium containing 0.5% trypan blue solution (Solarbio, Beijing, China) for 3 min. The number of dead cells was counted as a percentage using Countsee (Invitrogen, USA).

### Colony formation assay

For the effect of microcolin H on growth ability, a colony formation assay was performed in HGC-27 and MKN-28 cells. Then, 1 × 10^3^ cells were seeded in 6-well plates and treated with different concentrations of microcolin H for 48 h, then changed microcolin H with complete medium. Cells were monitored for colony formation assay for 9 days and replaced with a fresh medium every 3 days. The cells were fixed with 4% fixative solution for 10 min, followed by staining with 0.1% crystal violet solution at room temperature for 10 min. Prior to scanning, the plates were rinsed with distilled water and dried.

### Wound healing assay

Wound healing assay was performed in HGC-27 and MKN-28 cells. When the cells were over 90% confluent, the cell monolayer was scratched to the same width using a yellow pipette tip. After washing with PBS three times, these cells were then treated for 48 h with microcolin H at different concentrations. After treatment, an inverted microscope was used to capture the images. ImageJ software (National Institutes of Health, USA) measured wound healing percentages and calculated them by the formula: area recovery = (gap area before treatment − gap area after treatment) / gap area before treatment.

### Labelling of microcolin H bound proteins

GC cancer cells were lysed by brief sonication using lysate buffer (0.1% triton in PBS). The supernatant was collected after centrifugation at 15,000×*g* for 20 min and adjusted protein concentrations to 1 mg/mL with the BCA assay (Thermo Fisher Scientific, Waltham, MA, USA). Microcolin H (10 µM) or Probe 4 (10 nM-1 µM) were added and each tube was incubated for 1 h. Clicked with TAMRA-N_3_ under standard click chemistry conditions for 1 h (0.01 mM TAMRA-N_3_ in DMSO, 0.1 mM TBTA in DMSO, 1 mM TCEP in deionized water, and 1 mM CuSO_4_ in deionized water). After the reaction, 5×Loading buffer was added to each tube, followed by vortex. The mixture was heated to 95 °C for 10 min. The resulting proteins were resolved by 10% SDS-PAGE. Fluorescence images are shown in grayscale.

### In vitro pull-down assay

HGC-27 cells were harvested and lysed by brief sonication in lysate buffer (0.1% triton in PBS). After centrifugation at 15,000×*g* for 20 min, the supernatant was collected and adjusted protein concentrations to 3 mg/mL with the BCA assay (Thermo Fisher Scientific, Waltham, MA, USA). Microcolin H (25 µM) or Probe 4 (0.5 µM) were added and each tube was incubated for 1 h. Clicked with Biotin-TAMRA-N_3_ under standard click chemistry conditions for 1 h (0.01 mM Biotin-TAMRA-N_3_ in DMSO, 0.1 mM TBTA in DMSO, 1 mM TCEP in deionized water, and 1 mM CuSO_4_ in deionized water). After the reaction, each group was added pre-cooled acetone to precipitate protein at −80 °C for 50 min. The precipitated proteins were washed with −80 °C methanol and re-dissolved in 1 mL of 1.2% SDS/PBS and heated to 80–90 °C for 5 min. The dissolved protein solution was diluted to 0.2% SDS with 5 mL of PBS and incubated with streptavidin beads at room temperature for 1 h. After that, the streptavidin beads were washed five times with 0.2% SDS/PBS buffer and five times with PBS, the bead-bound proteins were eluted, separated by SDS-PAGE, and visualised by fluorescence.

### Proteomics assay

The samples were separated by SDS-PAGE, and the specific bands were isolated. The gels were dried with ACN for 10 min at room temperature. The in-gel proteins were reduced with 10 mM DTT (dithiothreitol) in 50 mM NH_4_HCO_3_ for 30 min at 56 °C and then alkylated with 55 mM IAA (iodoacetamide) in 50 mM NH_4_HCO_3_ for 1 h at room temperature in the dark. Then the gels were washed with water and acetonitrile for 10 min respectively. Gel pieces were digested in 10 ng/µL trypsin for 16 h at 37 °C. Extracted the peptides twice with 50% ACN/5% FA, combined the extracts and completely concentrated it by a vacuum centrifuge. The trypsin-digested samples were injected for nano LC-MS/MS analysis.

### Docking simulation

The crystal structure of PITPNA (PDB ID: 1UW5) was retrieved from the Protein Data Bank (www.rcsb.org). The sequence of human PITPNB (Uniport ID: P48739) was queried using the Uniport database. A homology model of human PITPNB structure based on mouse PITPNB (PDB ID: 2A1L) was predicted for SWISS-Model server. All the computational studies were performed using Schrödinger Suite v2020-4 software. All ligands were prepared with LigPrep and the proteins were prepared with Protein Preparation Wizard. Covalent docking was performed with CovDock. Cys94 was selected as the reactive residue and Michael addition was selected as the reaction type. A grid box of 15 Å was defined. All other parameters were set to defaults for the CovDock docking procedure.

### Thermal shift assay

The HGC-27 cell lysate (3.3 mg/mL) was treated with microcolin H (100 μM from a 100x stock in DMSO) or DMSO for 30 min at room temperature, each group was heated for 4 min at the selected temperature (35–77 °C). Then centrifugated for 15 min at 15,000 rpm at 4 °C. Harvested the supernatant carefully and the western blot experiment was performed subsequently.

### MST binding affinity

The interactions between the PITPβ and Microcolin H binding partners were measured in Monolith NT.115 Standard Treated Capillaries. The measurements were performed in buffer with 1% DMSO PBS Before the MST measurements, samples were centrifuged. The ligands for the binding studies were dissolved in target at double the concentration. The measurements were performed on a NanoTemper Technologies Monolith® NT.115 instrument. The samples were measured at high MST power and LED power of 100%. The data were analysed using MO.Affinity Analysis Software.

### Western blot assay

The harvested cells were lysed in 0.1% triton in PBS by brief sonication. Supernatants were collected after centrifugation at 15,000 × *g* for 20 min, and the concentration of whole protein was calculated using the BCA kit (#23227, Thermo Fisher Scientific, Waltham, MA, USA). After 10% SDS-PAGE separation, the protein was transferred into a PVDF membrane (Bio-Rad). Subsequently the membrane was blocked with 5% non-fat milk for 1 h at room temperature, and then incubated with the primary antibodies overnight at 4 °C. Above primary antibodies was following: anti-PITPNA (Affinity, DF9746, dilution 1: 1000), anti-PITPNB (Affinity, DF4291, dilution 1: 500), anti-GAPDH (Epizyme, LF206, dilution 1: 3000), anti-LC3 (CST, #3868, dilution 1:1000), anti-p62 (ProteinTech, 66184-1, dilution 1:1000), anti-Bcl2 (Abcam, ab32124, dilution 1:1000), anti-PARP (CST, #9532, dilution 1:1000), anti-Caspase-3 (Abcam, ab32351, dilution 1:5000), anti-Cleaved Caspase-3 (Abcam, ab32042, dilution 1:500), anti-Beclin1 (CST, #3495, dilution 1:1000). After that, incubation with the appropriate secondary antibodies (Beyotime, A0208, dilution 1:2000), the membranes were then visualised by ECL detection (NCM, Suzhou, China).

### Flow cytometry assay

For cell apoptosis analysis, 1 × 10^6^ cells were treated with microcolin H for 24 h. Then cells were washed by cold PBS three times before incubating with Annexin V-FITC/PI (Yuheng Biotechnology, Suzhou, China) according to the manufacturer’s instructions. Data were acquired by BD LSRFortessa Flow Cytometer (BD Biosciences, Franklin Lake, NJ, USA) and analysed using the FlowJo software (BD Biosciences, USA).

### Real-time cell imaging

HeLa-Difluo™ hLC3 cells were monitored with a high content analysis system (PerkinElmer, Operetta CLS). HeLa-Difluo™ hLC3 cells were seeded in a 96-well plate (5 × 10^3^ cells/well) and cultured overnight. Cells were treated with 50 nM microcolin H or DMSO and dynamically monitored by a content analyser for 48 h.

### Cell lines transfection

For PITPα and PITPβ knockout, the dual gRNA was designed and cloned into the CRISPR/CAS9 system vector pSpCas(BB)-2A-Puro(PX459)V2.0 (Genecarer, Xi’an, China). Briefly, PITPα KO 1# sequences was followed: gRNA-F: 5′-CTCTGTCCTTTAGTATCAAG-3′, gRNA-R: 5′-AAGTGGAGAGCTATCGAGCG -3′; PITPα KO 2# sequences was followed: gRNA-F: 5′-TTCTCACCGTCCTTCTCGTA-3′, gRNA-F: 5′-GCAGCTTAGTATGGGATCGG-3′. And PITPβ KO 1# sequences was followed: gRNA-F: 5′-CCAAGGAGCAAGATAGTTAG-3′, gRNA-R: 5′-TTGmicrocolin HTTTCAGTATCAGGT-3′; PITPβ KO 2# sequences was followed: gRNA-F: 5′-GGTCCGTATACTTATTTCGA-3′, gRNA-F: 5′-GCTAGTAAGAATGAGACTGG-3′. MKN-28, HGC-27 and Hela-GFP-LC3 cells were transfected with plasmids for PITPα/β knockout or vector control plasmid with Lipofectamine 2000 (#11668019, Thermo Fisher, US) for 48 h followed by puromycin (PU) selection (5 μg/ml) (# P8230, Solarbio, Beijing, China). The knockout and over expression efficiency were verified by western blot with anti-PITPNA (Affinity, DF9746, dilution 1:1000) and anti-PITPNB (Affinity, DF4291, dilution 1:500) antibodies.

### Co-immunoprecipitation (Co-IP)

The effect of microcolin H on the protein-protein interaction between Becline-1 and Bcl-2 was detected by Co-immunoprecipitation assay. A total of 1 × 10^6^ cells was treated with microcolin H at the indicated concentration for 6 h. Whole protein was extracted with RIPA lysis supplemented 1 mM PMSF and 1x phosphatase inhibitor cocktail. Then, 100 μg of whole lysates was immunoprecipitated with antibodies anti-Bcl-2 (#ab32124, Abcam) or anti-IgG (#7074, CST) diluted at 1:100 at 4 °C overnight. The precipitates were followed by incubation with 40 μL protein A+G agarose beads (#P2055, Beyotime) at 4 °C for 3 h and purification with cold PBS three times subsequently. The precipitates were immunoblotted with the indicated primary antibodies and detected with the Chemiluminescence Imaging System.

### Immunohistochemistry (IHC)

PITPα/β protein, LC3 and Ki67 expression were examined by IHC assay in a human gastric cancer tissue microarray (HStmA180Su09, Superchip, Shanghai, China) and tumour xenografts derived from HGC-27. IHC staining was performed following the experimental procedures as described previously. The sections were dyed with the indicated primary antibodies (diluted at 1:100) at 4 °C overnight; then, slides were washed with cold PBS buffer and incubated with HRP-conjugated secondary antibody at room temperature for 30 min for visualising the protein expression with DAB staining (Service, Wuhan, China). For histomorphometric analysis, hematoxylin and eosin (HE) staining was carried out in tissue sections. The sections were acquired using an NIS-element imaging system (Nikon, Japan).

### Mouse models

This study was approved by the Ethics Committee of Lanzhou University (jcyxy20210501) and was conducted in accordance with the Declaration of Helsinki. Female athymic nude Balb/c mice aged 5–6 weeks were purchased from GemPharmtechnology (Jiangsu, China), keeping in the standard specific pathogen-free (SPF) condition. A total of 5 × 10^6^ HGC-27 cells was suspended in a 100 μL mixture of Matrigel matrix and PBS (1:2) and injected into the right flank of nude mice. All the mice were divided into six groups randomly until tumour volume reached 150 mm^3^. The treatment groups were intraperitoneally injected with microcolin H (1 mg/kg, 5 mg/kg, and 10 mg/kg), paclitaxel (PTX, 8 mg/kg), HCQ-microcolin H (50 mg/kg+10 mg/kg) daily for 11 days while the control group was injected with 0.15 mL of PBS daily. Tumour volume was measured every day after the injection. The tumour size was calculated with the following formula: tumour volume = 1/2 x length (mm) x width (mm)^2^. At the end of the mouse experiment, blood samples were collected and centrifuged to obtain serum for blood biochemical markers analysis. Subsequently, the main organs (Heart, liver, spleen, lung, and kidney) and tumours were weighed and photographed after the mice were euthanized. All the tissues were fixed in 4% formalin and embedded in paraffin for tissue sections and the remaining was saved at −80 °C.

### Tissue microassay (TMA) information

The expression of PITPα/β in gastric cancer and adjacent tissues was determined by IHC. Gastric carcinoma and adjacent tissue samples collected from gastric cancer patients who underwent radical gastrectomy without chemotherapy or radiation were purchased from Shanghai Outdo Biotech CO., LTD (HStmA180Su09, China), and the informed consent was obtained from all patients.

### Statistical analysis

All data was presented as the mean ± standard error of the mean (SEM) in GraphPad Prism 7.00. The data was repeated in at least three independent experiments. The statistical significance between different groups was evaluated as One-way ANOVA with Tukey’s test and two-tailed Student’s *t*-test. For the overall analysis of PITPα/β expression Log-rank test was used in the Kaplan–Meier analysis (http://kmplot.com). The significant difference is identified as the following: *P < 0.05, **P < 0.01, ***P < 0.001.

### Supplementary information

Supplementary-information

Movie 1

Movie 2

## Data Availability

All data will be available upon reasonable request.
