# Supplementary material for: Microcolin H, a novel autophagy inducer, exerts potent antitumour activity by targeting PITPα/β
Source: Signal Transduct Target Ther. 2023 Nov 15;8:428. doi: 10.1038/s41392-023-01667-2 (PMC10645841; doi:10.1038/s41392-023-01667-2)
Supplement: Supplementary file 1 — Supplementary-information [file 41392_2023_1667_MOESM1_ESM.docx]

Supplementary Materials for

Microcolin H, a novel autophagy inducer, exerts potent antitumour activity by targeting PITPα/β

Hange Yang^1,#^, Xiaowei Zhang ^1,#^, Cong Wang^1,#^, Hailong Zhang^1,^ ^#, *^, Juan Yi^1^, Kun Wang^2^, Yanzhe Hou^1^, Peihong Ji^1^, Xiaojie Jin^3,^, Chenghao Li^3^, Min Zhang^3^, Shan Huang^1^, Haoyuan Jia^1,4^, Kuan Hu^2^, Lingyun Mou^1,4*^, Rui Wang^1,2,*^

Correspondence to: zhanghailong@lzu.edu.cn or muly@lzu.edu.cn or wangrui@lzu.edu.cn

**This PDF file includes:**

Materials for organic synthesis

Methods for organic synthesis

Figures. S1 to S46

Movies S1 to S2

Materials and Methods

**Materials for organic synthesis**

Unless stated otherwise, all reactions were carried out in flame dried glassware. All solvents were purified and dried according to standard methods before use. TFA = trifluoroacetic acid, Bop-Cl = Bis(2-oxo-3-oxazolidinyl)phosphinic chloride, HATU = O-(7-Azabenzotriazol-1-yl)-N,N,N',N'-tetraMethyluroniuM hexafluorophosphate, TBS-Cl = tert-Butyldimethylsilyl chloride, LiHMDS = Lithium bis(trimethylsilyl)amide, DCC = N,N'-Dicyclohexylcarbodiimide. Reagents were purchased from Energy chemical and Bidepharm, and were used without further purification. Reactions were monitored by thin layer chromatography (TLC) carried out on silica gel plates using UV light as visualizing agent and aqueous phosphomolybdic acid as a developing agent. 1H NMR and 13C NMR were recorded on a Bruker instrument (300MHz and 75 MHz, respectively) and calibrated using internal references and solvent signals CHCl3 (δH = 7.26 ppm, δC = 77.16 ppm), DMSO-d6 (δH = 2.50 ppm, δC = 39.52 ppm). 1H NMR data are reported as follows: chemical shift, multiplicity (s = singlet, d = doublet, t = triplet, q = quartet, br = broad, m = multiplet), coupling constants and integration. Date for 13C NMR were reported in terms of chemical shift (δ, ppm). High-resolution mass spectra (HRMS) were obtained by the ESI ionization sources.

**Methods for organic synthesis**

**(R)-2-methyloctanoic acid M1**

(R)-4-benzyloxazolidin-2-one **1** (4.32 g, 24.45 mmol) was dissolved in dry THF (75 mL) followed by the addition of butyl lithium (9.78 mL, 24.48 mmol) dropwise under argon at -78℃. The reaction was stirred at that temperature for 10 minutes, then octanoyl chloride (4.59 mL, 26.88 mmol) was added dropwise. Stirring was continued for 30 minutes at -78℃, then the reaction temperature was raised to gradually room temperature. The reaction was diluted with a saturated solution of NH_4_Cl. THF was evaporated at reduced pressure and the reaction was extracted with EtOAc (3×100 mL). The combined organic layers were washed with brine and dried over MgSO_4._ Concentrated and purified by silica column chromatography (hexanes/EtOAc=5/1) yielded the product (R)-4-benzyl-3-octanoyloxazolidin-2-one **2** (6.0 g, 19.8 mmol, 81.0% yield). To a stirring solution of (R)-4-benzyl-3-octanoyloxazolidin-2-one **2** (3.5 g, 11.6 mmol) in THF (15 mL) at -78℃ was added NaN(SiMe_3_)_2_ (12.7 mL, 25.4 mmol) dropwise and the mixture was stirred 30min at this temperature. Then MeI (1.45 mL, 23.2 mmol) was added to the reaction mixture and the reaction was stirred at -78℃ for 5 h. The reaction mixture was quenched with a saturated solution of NH_4_Cl and extracted with EtOAc (3×100 mL), the combined organic layers were washed with 5% NaS_2_O_3_ solution, brine, and dried over MgSO_4_. Concentrated and purified by silica column chromatography (hexanes/EtOAc=8/1). The purified intermediate was dissolved in THF (90 mL) and H_2_O (30 mL) and added LiOH (0.62 g, 15.8 mmol), H_2_O_2_ (7 mL, 63 mmol) at 0℃. After 4 h stirring at 0℃, 1M Na_2_S_2_O_3_ solution was added and adjusted pH to 1.0 with 6M HCl. The mixture was extracted with DCM three times. The combined organic layers were washed with brine and purified by silica column chromatography (hexanes/EtOAc=8/1) to get (R)-2-methyloctanoic acid **M1** (550 mg, 3.5 mmol, 30% yield) as a clear oil. ^1^H NMR (300 MHz, Chloroform-*d*) δ 2.39 (h, *J* = 6.9 Hz, 1H), 1.61 (dd, *J* = 14.1, 7.2 Hz, 1H), 1.37 (dd, *J* = 7.2, 2.9 Hz, 1H), 1.21 (q, *J* = 6.8, 5.7 Hz, 9H), 1.11 (d, *J* = 7.0 Hz, 3H), 0.84 – 0.78 (m, 3H).

**benzyl N-(tert-butoxycarbonyl)-N-methyl-L-valinate 3**

To a solution of N-(tert-butoxycarbonyl)-N-methyl-L-valine (4.64 g, 20 mmol) and potassium carbonate (6.07 g, 44 mmol) in acetonitrile (100 mL) was added benzyl bromide (4.27 mL, 32 mmol) dropwise and was stirred at room temperature for 4 h. The mixture was concentrated under reduced pressure and purified by silica column chromatography (hexanes/EtOAc=10/1) to get benzyl N-(tert-butoxycarbonyl)-N-methyl-L-valinate **3** (5.64 g, 17.6 mmol, 88% yield) as a white foam. ^1^H NMR (300 MHz, Chloroform-d) δ 7.34 (s, 5H), 5.16 (s, 2H), 4.33 (dd, J = 107.1, 10.5 Hz, 1H), 2.80 (d, J = 21.9 Hz, 3H), 2.19 (dq, J = 11.3, 6.5, 5.7 Hz, 1H), 1.44 (d, J = 11.4 Hz, 9H), 0.96 (d, J = 6.5 Hz, 3H), 0.89 (d, J = 5.3 Hz, 3H). ^13^C NMR (75 MHz, Chloroform-*d*) δ 171.35, 170.94, 156.22, 135.87, 128.53, 128.44, 128.24, 128.05, 127.96, 79.94, 66.18, 65.11, 63.24, 30.44, 28.33, 27.81, 20.00, 19.06.

**benzyl N-((tert-butoxycarbonyl)-L-threonyl)-N-methyl-L-valinate 5**

To a solution of benzyl N-(tert-butoxycarbonyl)-N-methyl-L-valinate **3** (5.64 g, 17.6 mmol) in DCM (40 mL) was added TFA (10 mL) dropwise at 0℃ and was stirred at room temperature for 1 h. The mixture was concentrated under reduced pressure. The residue was added DCM (3×30 mL) and concentrated to yield benzyl methyl-L-valinate. At the same time, to a stirring solution of (tert-butoxycarbonyl)-L-threonine **4** (4.7 g, 21.3 mmol) in DCM (50 mL) at 0℃ was added Bop-Cl (5.5 g, 21.3 mmol) and the mixture was stirred 10 min at this temperature. Then, a solution of benzyl methyl-L-valinate in DCM (50 mL) with DIPEA (9.3 mL, 53.3 mmol) was transferred to the pre-activated (tert-butoxycarbonyl)-L-threonine solution, and the reaction was allowed to reach room temperature and stirred overnight. The mixture was concentrated, dissolved in H_2_O, and extracted with EtOAc three times. The combined organic layers were washed with brine, dried over MgSO_4_, and concentrated. The crude residue was purified by silica column chromatography (hexanes/EtOAc=4/1) to get benzyl N-((tert-butoxycarbonyl)-L-threonyl)-N-methyl-L-valinate **5** (2.8 g, 6.7 mmol, 38% yield) as a foamy-like off white solid. ^1^H NMR (300 MHz, Chloroform-d) δ 7.34 (d, J = 1.0 Hz, 5H), 5.41 (d, J = 9.5 Hz, 1H), 5.25 – 5.06 (m, 2H), 4.90 (d, J = 10.5 Hz, 1H), 4.42 (dd, J = 9.5, 2.1 Hz, 1H), 3.97 (dd, J = 6.4, 2.0 Hz, 1H), 3.72 (s, 1H), 3.03 (s, 3H), 2.32 – 2.16 (m, 1H), 1.40 (d, J = 13.3 Hz, 9H), 1.14 (d, J = 6.4 Hz, 3H), 1.00 (d, J = 6.6 Hz, 3H), 0.84 (d, J = 6.7 Hz, 3H). ^13^C NMR (75 MHz, Chloroform-*d*) δ 173.61, 170.31, 156.15, 135.43, 128.59, 128.48, 128.41, 128.04, 80.07, 67.26, 66.72, 64.92, 61.74, 53.53, 31.55, 29.76, 28.21, 28.16, 27.29, 19.80, 18.63, 18.23.

**benzyl N-(O-acetyl-N-(tert-butoxycarbonyl)-L-threonyl)-N-methyl-L-valinate 6**

To a solution of benzyl N-((tert-butoxycarbonyl)-L-threonyl)-N-methyl-L-valinate **5** (1.4 g, 3.31 mmol) and DMAP (0.485 g, 3.97 mmol) in EtOAc (20 mL) was added Ac_2_O (0.38 mL, 3.97 mmol) dropwise and was stirred at room temperature for 5 h. The mixture was concentrated under reduced pressure and was purified by silica column chromatography (hexanes/EtOAc=4/1) to provide benzyl N-(O-acetyl-N-(tert-butoxycarbonyl)-L-threonyl)-N-methyl-L-valinate **6** as an off-white foam (1.26g, 2.71 mmol, 82% yield). ^1^H NMR (300 MHz, Chloroform-d) δ 7.34 (s, 5H), 5.39 (d, J = 9.3 Hz, 1H), 5.22 (d, J = 6.0 Hz, 1H), 5.15 (s, 2H), 4.91 (d, J = 10.6 Hz, 1H), 4.67 (dd, J = 9.3, 5.4 Hz, 1H), 3.05 (s, 3H), 2.23 (dt, J = 10.6, 6.6 Hz, 1H), 1.96 (s, 3H), 1.42 (s, 9H), 1.16 (d, J = 6.4 Hz, 3H), 0.99 (d, J = 6.5 Hz, 3H), 0.83 (d, J = 6.7 Hz, 3H). ^13^C NMR (75 MHz, Chloroform-*d*) δ 170.59, 170.26, 170.17, 155.74, 135.51, 128.51, 128.35, 128.18, 79.98, 70.37, 69.46, 66.71, 65.37, 61.77, 54.04, 31.35, 29.69, 28.23, 27.17, 20.95, 19.70, 18.60, 17.35, 16.71.

**benzyl N-(O-acetyl-N-(N-(tert-butoxycarbonyl)-N-methyl-L-leucyl)-L-threonyl)-N-methyl-L-valinate 8**

To a solution of benzyl N-(O-acetyl-N-(tert-butoxycarbonyl)-L-threonyl)-N-methyl-L-valinate **6** (1 g, 2.08 mmol) in DCM (20 mL) was added TFA (10 mL) dropwise at 0℃ and was stirred at room temperature for 1 h. The mixture was concentrated under reduced pressure. The residue was added DCM (3×30 mL) and concentrated to yield benzyl N-(O-acetyl-L-threonyl)-N-methyl-L-valinate. At the same time, to a stirring solution of N-(tert-butoxycarbonyl)-N-methyl-L-leucine **7** (0.61 g, 2.5 mmol) in DMF (15 mL) at 0℃ was added HATU (0.95 g, 2.5 mmol) and DIPEA (0.83 mL, 5.0 mmol). The mixture was stirred for 10 mins at this temperature. Then, a solution of pre-activated benzyl N-(O-acetyl-L-threonyl)-N-methyl-L-valinate in DMF (15 mL) with DIPEA (0.83 mL, 5.0 mmol) was transferred to the N-(tert-butoxycarbonyl)-N-methyl-L-leucine solution and the reaction was allowed to reach room temperature and stirred overnight. The mixture was dissolved in H_2_O and extracted with EtOAc three times. The combined organic layers were washed with brine, dried over MgSO_4_, and concentrated. The crude residue was purified by silica column chromatography (hexanes/EtOAc=5/1) to get N-(O-acetyl-N-(N-(tert-butoxycarbonyl)-N-methyl-L-leucyl)-L-threonyl)-N-methyl-L-valinate **8** (0.8 g, 1.35 mmol, 65% yield) as a clear semi-solid. ^1^H NMR (300 MHz, Chloroform-*d*) δ 7.34 (s, 5H), 6.81 (d, *J* = 34.4 Hz, 1H), 5.25 (s, 1H), 5.15 (s, 2H), 4.99 (dd, *J* = 8.7, 5.0 Hz, 1H), 4.91 (d, *J* = 10.6 Hz, 1H), 4.77 – 4.55 (m, 1H), 3.06 (s, 3H), 2.76 (s, 3H), 2.22 (dt, *J* = 10.7, 6.5 Hz, 1H), 1.93 (s, 3H), 1.75 (s, 2H), 1.49 (s, 10H), 1.12 (d, *J* = 6.4 Hz, 3H), 0.99 – 0.89 (m, 9H), 0.81 (d, *J* = 6.7 Hz, 3H). ^13^C NMR (75 MHz, Chloroform-*d*) δ 171.47, 171.27, 170.18, 170.02, 169.72, 169.36, 135.50, 128.51, 128.44, 128.35, 69.26, 66.72, 61.75, 57.17, 56.09, 52.48, 52.23, 36.51, 31.25, 29.68, 29.21, 28.33, 27.15, 24.50, 23.26, 21.78, 21.32, 20.87, 19.62, 18.63, 16.74.

**N-(O-acetyl-N-(N-(tert-butoxycarbonyl)-N-methyl-L-leucyl)-L-threonyl)-N-methyl-L-valine M2**

To a solution of N-(O-acetyl-N-(N-(tert-butoxycarbonyl)-N-methyl-L-leucyl)-L-threonyl)-N-methyl-L-valinate **8** (0.72 g, 1.22 mmol) in MeOH (30 mL) was added Pd/C 10%Wt (160 mg). The reaction vessel was evacuated/backfilled with H_2_ and stirred under this atmosphere for 2 h. The mixture was filtered through celatom, washed with MeOH, and concentrated to give N-(O-acetyl-N-(N-(tert-butoxycarbonyl)-N-methyl-L-leucyl)-L-threonyl)-N-methyl-L-valine **M2** (0.55 g, 1.1 mmol, 90% yield) as a clear semi-solid.

.

**For the synthesis of M3,** see *Org. Lett.***2019,** 21, 3, 793–796.

**N-Boc pentapeptide M4**

N-Boc-OTBS-Pyrrylproline enone **12** (350 mg, 0.825 mmol) was dissolved in 4M HCl-Dioxane at 0℃ for 1 h. The mixture was concentrated under reduced pressure to get **M3**. The residue was added DCM (3×30 mL) and concentrated to removed excess acid. The residue was added N-(O-acetyl-N-(N-(tert-butoxycarbonyl)-N-methyl-L-leucyl)-L-threonyl)-N-methyl-L-valine **M2** (456 mg, 0.91 mmol), BOP-Cl (233 mg, 0.91 mmol) and DCM (30 mL). To the mixture solution was added TEA (0.93 mL, 6.6 mmol) dropwise at 0℃ and stirred overnight. The mixture was concentrated and purified by silica column chromatography (hexanes/EtOAc=1/6) to get N-Boc pentapeptide **M4** (291 mg, 0.42 mmol, 51% yield) as a clear oil. ^1^H NMR (300 MHz, Chloroform-*d*) δ 7.29 (d, *J* = 2.1 Hz, 1H), 6.90 (d, *J* = 9.1 Hz, 1H), 6.09 (dd, *J* = 6.0, 1.6 Hz, 1H), 5.74 – 5.53 (m, 1H), 5.27 (s, 1H), 5.04 (s, 1H), 5.03 – 5.00 (m, 1H), 4.82 (dt, *J* = 6.8, 1.9 Hz, 1H), 4.37 (d, *J* = 6.0 Hz, 1H), 3.85 (dd, *J* = 21.6, 12.5 Hz, 2H), 3.11 (s, 3H), 2.78 (s, 3H), 2.49 – 2.44 (m, 1H), 2.28 – 2.21 (m, 1H), 1.99 (d, *J* = 2.4 Hz, 4H), 1.69 (d, *J* = 9.6 Hz, 1H), 1.50 (s, 10H), 1.48 – 1.45 (m, 3H), 1.43 (d, *J* = 5.6 Hz, 1H), 1.20 (d, *J* = 6.4 Hz, 3H), 0.99 (d, *J* = 6.5 Hz, 3H), 0.94 – 0.88 (m, 6H), 0.81 (d, *J* = 6.7 Hz, 3H). ^13^C NMR (75 MHz, Chloroform-*d*) δ 174.59, 174.47, 173.47, 168.97, 168.89, 168.82, 167.83, 153.22, 124.28, 70.79, 67.56, 58.26, 58.06, 57.61, 57.13, 55.85, 52.48, 50.98, 35.54, 30.85, 29.45, 28.54, 27.32, 26.12, 24.21, 23.73, 22.19, 20.70, 19.96, 18.09, 17.80, 17.34, 16.43, 15.87.

**Microcolin H**

N-Boc pentapeptide **M4** (409 mg, 0.59 mmol) dissolved in DCM (20 mL) was added TFA (10 mL) dropwise at 0℃ and was stirred at room temperature for 1 h. The mixture was concentrated under reduced pressure. The residue was added DCM (3×30 mL) and concentrated to remove excess acid. The residue was added (R)-2-methyloctanoic acid **M1** (102.7 mg, 0.65 mmol), BOP-Cl (165.8 mg, 0.65 mmol) and DCM (30 mL). To the mixture solution was added TEA (0.73 mL, 4.72 mmol) dropwise at 0℃ and stirred overnight. The mixture was concentrated and purified by silica column chromatography (hexanes/EtOAc=1/8) to get microcolin H (209 mg, 0.29 mmol, 49% yield) as a clear oil. ^1^H NMR (300 MHz, Chloroform-*d*) δ 7.29 (d, *J* = 2.1 Hz, 1H), 6.98 (d, *J* = 8.9 Hz, 1H), 6.09 (dd, *J* = 6.1, 1.6 Hz, 1H), 5.67 (dd, *J* = 10.1, 2.1 Hz, 1H), 5.28 (dd, *J* = 10.9, 6.2 Hz, 2H), 5.02 (d, *J* = 11.1 Hz, 1H), 4.86 – 4.77 (m, 1H), 4.43 – 4.33 (m, 1H), 3.87 – 3.77 (m, 2H), 3.10 (s, 3H), 2.94 (s, 3H), 2.72 (q, *J* = 6.8 Hz, 1H), 2.48 – 2.42 (m, 1H), 2.32 – 2.22 (m, 1H), 2.00 (s, 4H), 1.79 (d, *J* = 8.8 Hz, 1H), 1.73 – 1.69 (m, 1H), 1.60 (dd, *J* = 9.2, 5.6 Hz, 1H), 1.47 (d, *J* = 6.7 Hz, 4H), 1.26 (s, 9H), 1.14 (t, *J* = 6.5 Hz, 6H), 0.97 (dd, *J* = 12.5, 6.5 Hz, 6H), 0.90 – 0.85 (m, 6H), 0.82 (d, *J* = 6.6 Hz, 3H). ^13^C NMR (75 MHz, Chloroform-d) δ 177.84, 174.21, 171.23, 169.86, 169.80, 169.68, 168.71, 154.16, 125.26, 71.56, 68.42, 59.12, 58.58, 58.08, 56.70, 53.81, 51.84, 36.76, 36.12, 35.82, 34.15, 31.71, 30.42, 30.37, 29.19, 27.36, 27.11, 24.74, 23.29, 22.50, 21.58, 20.98, 18.78, 18.36, 17.54, 17.33, 16.91, 14.03. HRMS (ESI) calcd for C_38_H_63_N_5_NaO_9_ [M ^+^ Na^+^] 756.4518; found, 756.4502.

**Analogues(A1-A5) and probes(1-5) of microcolin H**

N-Boc pentapeptide **M4** (13 mg, 0.019 mmol) dissolved in DCM (20 mL) was added TFA (10 mL) dropwise at 0℃ and was stirred at room temperature for 1 h. The mixture was concentrated under reduced pressure. The residue was added DCM (3×30 mL) and concentrated to removed excess acid. The residue was added acetic acid (2 mg, 0.03375 mmol), BOP-Cl (8.6 mg, 0.03375 mmol), and DCM (1 mL). To the mixture solution was added TEA (0.0065 mL, 0.042 mmol) dropwise at 0℃ and stirred overnight. The mixture was concentrated and purified by silica column chromatography to get **A-1** (5.5 mg, 0.0086 mmol, 38.5% yield) as a clear oil. ^1^H NMR (300 MHz, Chloroform-*d*) δ 7.29 (d, *J* = 2.1 Hz, 1H), 6.91 (d, *J* = 8.6 Hz, 1H), 6.09 (dd, *J* = 6.0, 1.6 Hz, 1H), 5.69 – 5.56 (m, 1H), 5.28 – 5.15 (m, 2H), 5.05 – 4.99 (m, 1H), 4.87 – 4.74 (m, 1H), 4.38 (s, 1H), 3.84 (dd, *J* = 10.3, 6.2 Hz, 2H), 3.11 (s, 3H), 2.93 (s, 3H), 2.46 (dd, *J* = 9.6, 4.8 Hz, 1H), 2.26 (d, *J* = 4.8 Hz, 1H), 2.15 (s, 3H), 2.02 (d, *J* = 2.2 Hz, 4H), 1.69 – 1.63 (m, 2H), 1.47 (d, *J* = 6.7 Hz, 3H), 1.42 (s, 1H), 1.18 (d, *J* = 6.5 Hz, 3H), 0.99 (d, *J* = 6.5 Hz, 3H), 0.92 (dd, *J* = 13.0, 6.6 Hz, 6H), 0.80 (d, *J* = 1.6 Hz, 3H). HRMS (ESI) calcd for C_31_H_49_N_5_NaO_9_ [M ^+^ Na^+^] 658.3422; found, 658.3407.

N-Boc pentapeptide **M4** (13 mg, 0.019 mmol) was dissolved in 4M HCl-Dioxane at 0℃ for 1 h. The mixture was concentrated under reduced pressure. The residue was added DCM (3×30 mL) and concentrated to removed excess acid. The residue was added butyric acid (3mg, 0.03375 mmol), BOP-Cl (8.6 mg, 0.03375 mmol), and DCM (1 mL). To the mixture solution was added TEA (0.0065 mL, 0.042 mmol) dropwise at 0℃ and stirred overnight. The mixture was concentrated and purified by silica column chromatography to get **A-2** (5.4 mg, 0.0081 mmol, 36% yield) as a clear oil. ^1^H NMR (300 MHz, Chloroform-*d*) δ 7.29 (d, *J* = 2.0 Hz, 1H), 6.94 (d, *J* = 8.8 Hz, 1H), 6.09 (dd, *J* = 6.1, 1.6 Hz, 1H), 5.66 (dd, *J* = 10.0, 2.2 Hz, 1H), 5.24 (q, *J* = 7.9, 6.6 Hz, 2H), 5.06 – 4.99 (m, 1H), 4.86 – 4.77 (m, 1H), 4.38 (s, 1H), 3.88 – 3.77 (m, 2H), 3.11 (s, 3H), 2.92 (s, 3H), 2.46 (dt, *J* = 9.7, 4.4 Hz, 1H), 2.35 (s, 2H), 2.28 – 2.22 (m, 1H), 2.00 (d, *J* = 2.3 Hz, 4H), 1.70 (d, *J* = 8.1 Hz, 2H), 1.64 (d, *J* = 7.8 Hz, 2H), 1.47 (d, *J* = 6.7 Hz, 3H), 1.40 (d, *J* = 7.0 Hz, 1H), 1.29 (d, *J* = 24.0 Hz, 3H), 1.17 (d, *J* = 6.6 Hz, 3H), 1.00 (s, 3H), 0.93 (s, 3H), 0.89 (d, *J* = 6.5 Hz, 3H), 0.81 (d, *J* = 6.6 Hz, 3H). HRMS (ESI) calcd for C_33_H_53_N_5_NaO_9_ [M ^+^ Na^+^] 686.3735; found, 686.3725.

N-Boc pentapeptide **M4** (13 mg, 0.019 mmol) was dissolved in 4M HCl-Dioxane at 0℃ for 1 h. The mixture was concentrated under reduced pressure. The residue was added DCM (3×30 mL) and concentrated to removed excess acid. The residue was added hexanoic acid (4 mg, 0.03375 mmol), BOP-Cl (8.6 mg, 0.03375 mmol), and DCM (1 mL). To the mixture solution was added TEA (0.0065 mL, 0.042 mmol) dropwise at 0℃ and stirred overnight. The mixture was concentrated and purified by silica column chromatography to get **A-3** (10 mg, 0.0145 mmol, 64.5% yield) as a faint yellow oil. ^1^H NMR (300 MHz, Chloroform-*d*) δ 7.29 (d, *J* = 2.2 Hz, 1H), 6.91 (d, *J* = 8.8 Hz, 1H), 6.09 (dd, *J* = 6.1, 1.7 Hz, 1H), 5.69 – 5.55 (m, 1H), 5.23 (q, *J* = 7.7 Hz, 2H), 5.01 (dd, *J* = 11.1, 5.6 Hz, 1H), 4.81 (d, *J* = 7.1 Hz, 1H), 4.37 (s, 1H), 3.83 (dd, *J* = 10.8, 6.5 Hz, 2H), 3.11 (t, *J* = 2.1 Hz, 3H), 2.92 (s, 3H), 2.49 – 2.42 (m, 1H), 2.33 (s, 1H), 2.01 (d, *J* = 2.5 Hz, 4H), 1.77 (s, 2H), 1.72 (s, 6H), 1.63 (s, 2H), 1.40 (s, 3H), 1.38 (s, 1H), 1.17 (d, *J* = 6.4 Hz, 3H), 0.99 (s, 6H), 0.93 (d, *J* = 3.2 Hz, 6H), 0.83 – 0.80 (m, 3H). HRMS (ESI) calcd for C_35_H_57_N_5_NaO_9_ [M ^+^ Na^+^] 714.4048; found, 714.4035.

N-Boc pentapeptide **M4** (13 mg, 0.019 mmol) was dissolved in 4M HCl-Dioxane at 0℃ for 1 h. The mixture was concentrated under reduced pressure. The residue was added DCM (3×30 mL) and concentrated to removed excess acid. The residue was added decanoic acid (5.8 mg, 0.03375 mmol), BOP-Cl (8.6 mg, 0.03375 mmol), and DCM (1 mL). To the mixture solution was added TEA (0.0065 mL, 0.042 mmol) dropwise at 0℃ and stirred overnight. The mixture was concentrated and purified by silica column chromatography to get **A-4** (13 mg, 0.0172 mmol, 76.5% yield) as a faint yellow oil. ^1^H NMR (300 MHz, Chloroform-*d*) δ 7.29 (s, 1H), 6.91 (d, *J* = 8.7 Hz, 1H), 6.09 (dd, *J* = 6.1, 1.6 Hz, 1H), 5.70 – 5.56 (m, 1H), 5.23 (q, *J* = 7.9 Hz, 2H), 5.01 (dd, *J* = 11.1, 5.7 Hz, 1H), 4.86 – 4.76 (m, 1H), 3.85 (dd, *J* = 12.0, 4.3 Hz, 2H), 3.11 (s, 3H), 2.92 (s, 3H), 2.50 – 2.42 (m, 1H), 2.26 (d, *J* = 5.0 Hz, 1H), 2.01 (d, *J* = 2.3 Hz, 4H), 1.67 (s, 5H), 1.45 (s, 8H), 1.40 (s, 3H), 1.17 (d, *J* = 6.5 Hz, 3H), 0.94 (s, 9H), 0.88 (dd, *J* = 4.4, 2.2 Hz, 9H), 0.84 – 0.79 (m, 3H). HRMS (ESI) calcd for C_39_H_65_N_5_NaO_9_ [M ^+^ Na^+^] 770.4647; found, 770.4655.

N-Boc pentapeptide **M4** (13 mg, 0.019 mmol) was dissolved in 4M HCl-Dioxane at 0℃ for 1 h. The mixture was concentrated under reduced pressure. The residue was added DCM (3×30 mL) and concentrated to removed excess acid. The residue was added lauric acid (6.8 mg, 0.03375 mmol), BOP-Cl (8.6 mg, 0.03375 mmol), and DCM (1 mL). To the mixture solution was added TEA (0.0065 mL, 0.042 mmol) dropwise at 0℃ and stirred overnight. The mixture was concentrated and purified by silica column chromatography to get **A-5** (14 mg, 0.018 mmol, 80.3% yield) as a faint yellow oil. ^1^H NMR (300 MHz, Chloroform-*d*) δ 7.29 (s, 1H), 6.92 (d, *J* = 8.8 Hz, 1H), 6.09 (dd, *J* = 6.0, 1.6 Hz, 1H), 5.66 (dd, *J* = 10.1, 2.2 Hz, 1H), 5.23 (q, *J* = 8.1 Hz, 2H), 5.01 (dd, *J* = 11.1, 5.8 Hz, 1H), 4.81 (d, *J* = 6.9 Hz, 1H), 3.83 (dd, *J* = 10.6, 6.5 Hz, 2H), 3.11 (s, 3H), 2.92 (s, 3H), 2.50 – 2.42 (m, 1H), 2.26 (d, *J* = 5.5 Hz, 1H), 2.01 (d, *J* = 2.4 Hz, 4H), 1.67 (s, 3H), 1.48 (s, 3H), 1.40 (s, 3H), 1.27 (s, 6H), 1.17 (d, *J* = 6.5 Hz, 3H), 0.99 (s, 8H), 0.94 (d, *J* = 3.2 Hz, 8H), 0.90 – 0.86 (m, 7H), 0.81 (d, *J* = 6.7 Hz, 3H). HRMS (ESI) calcd for C_41_H_69_N_5_NaO_9_ [M ^+^ Na^+^] 798.4987; found, 798.4970.

N-Boc pentapeptide **M4** (15.6 mg, 0.0225 mmol) was dissolved in 4M HCl-Dioxane at 0℃ for 1 h. The mixture was concentrated under reduced pressure. The residue was added DCM (3×30 mL) and concentrated to removed excess acid. The residue was added 4-pentynoic acid (3.3 mg, 0.03375 mmol), BOP-Cl (8.6 mg, 0.03375 mmol), and DCM (1 mL). To the mixture solution was added TEA (0.0065 mL, 0.042 mmol) dropwise at 0℃ and stirred overnight. The mixture was concentrated and purified by silica column chromatography to get **Probe 1** (7.4 mg, 49.0% yield) as a clear oil. ^1^H NMR (300 MHz, Chloroform-*d*) δ 7.31 – 7.27 (m, 1H), 6.84 (d, *J* = 8.8 Hz, 1H), 6.09 (dd, *J* = 6.1, 1.6 Hz, 1H), 5.66 (dd, *J* = 10.0, 2.1 Hz, 1H), 5.30 – 5.16 (m, 2H), 5.02 (d, *J* = 11.1 Hz, 1H), 4.95 (dd, *J* = 8.8, 3.7 Hz, 1H), 4.87 – 4.77 (m, 1H), 4.39 (s, 1H), 3.89 (d, *J* = 11.6 Hz, 1H), 3.80 (dd, *J* = 11.5, 4.3 Hz, 1H), 3.54 (d, *J* = 11.0 Hz, 1H), 3.49 (s, 1H), 3.11 (s, 3H), 2.94 (s, 3H), 2.66 – 2.55 (m, *J* = 4.0 Hz, 4H), 2.48 (ddd, *J* = 14.5, 10.0, 4.8 Hz, 1H), 2.34 – 2.21 (m, 1H), 2.06 (d, *J* = 2.3 Hz, 1H), 2.01 (d, *J* = 4.8 Hz, 3H), 1.99 (d, *J* = 2.5 Hz, 1H), 1.66 (ddd, *J* = 9.3, 6.1, 3.1 Hz, 2H), 1.47 (d, *J* = 6.8 Hz, 3H), 1.17 (d, *J* = 6.5 Hz, 3H), 0.99 (d, *J* = 6.5 Hz, 3H), 0.94 (d, *J* = 6.6 Hz, 3H), 0.89 (d, *J* = 6.5 Hz, 3H), 0.81 (d, *J* = 6.7 Hz, 3H).

N-Boc pentapeptide **M4** (15.6 mg, 0.0225 mmol) was dissolved in 4M HCl-Dioxane at 0℃ for 1 h. The mixture was concentrated under reduced pressure. The residue was added DCM (3×30 mL) and concentrated to removed excess acid. The residue was added 6-heptynoic acid (4.25 mg, 0.03375 mmol), BOP-Cl (8.6 mg, 0.03375 mmol), and DCM (1 mL). To the mixture solution was added TEA (0.0065 mL, 0.042 mmol) dropwise at 0℃ and stirred overnight. The mixture was concentrated and purified by silica column chromatography to get **Probe 2** (8.2 mg, 52.2% yield) as a clear semi-solid. ^1^H NMR (300 MHz, Chloroform-*d*) δ 7.29 (d, *J* = 2.2 Hz, 1H), 6.92 (d, *J* = 8.8 Hz, 1H), 6.09 (dd, *J* = 6.0, 1.6 Hz, 1H), 5.66 (dd, *J* = 10.1, 2.2 Hz, 1H), 5.24 (dt, *J* = 14.0, 5.7 Hz, 2H), 5.02 (d, *J* = 11.1 Hz, 1H), 4.95 (dd, *J* = 8.9, 3.5 Hz, 1H), 4.86 – 4.76 (m, 1H), 4.39 (s, 1H), 3.93 – 3.78 (m, 2H), 3.58 (s, 1H), 3.11 (s, 3H), 2.93 (s, 3H), 2.49 (td, *J* = 9.9, 5.0 Hz, 1H), 2.40 (t, *J* = 7.4 Hz, 2H), 2.24 (td, *J* = 6.9, 2.9 Hz, 4H), 2.05 (s, 1H), 2.02 (s, 3H), 1.96 (t, *J* = 2.7 Hz, 1H), 1.78 (q, *J* = 7.6, 7.2 Hz, 2H), 1.64 (q, *J* = 7.6 Hz, 4H), 1.47 (d, *J* = 6.8 Hz, 3H), 1.17 (d, *J* = 6.5 Hz, 3H), 0.99 (d, *J* = 6.5 Hz, 3H), 0.94 (d, *J* = 6.6 Hz, 3H), 0.89 (d, *J* = 6.4 Hz, 3H), 0.81 (d, *J* = 6.6 Hz, 3H). ^13^C NMR (75 MHz, Chloroform-*d*) δ 174.59, 173.68, 171.16, 169.87, 168.88, 154.19, 125.33, 84.04, 71.85, 68.61, 68.55, 59.20, 58.59, 58.12, 56.93, 54.16, 51.99, 36.57, 36.32, 33.21, 30.76, 30.45, 28.02, 27.15, 24.93, 24.10, 23.13, 21.92, 21.08, 18.84, 18.36, 18.24, 17.42, 16.92.

N-Boc pentapeptide **M4** (15.6 mg, 0.0225 mmol) was dissolved in 4M HCl-Dioxane at 0℃ for 1 h. The mixture was concentrated under reduced pressure. The residue was added DCM (3×30 mL) and concentrated to removed excess acid. The residue was added myristic acid alkyne (7.56 mg, 0.03375 mmol), BOP-Cl (8.6 mg, 0.03375 mmol), and DCM (1 mL). To the mixture solution was added TEA (0.0065 mL, 0.042 mmol) dropwise at 0℃ and stirred overnight. The mixture was concentrated and purified by silica column chromatography to get **Probe 3** (9.7 mg, 53.3% yield) as a clear oil. ^1^H NMR (300 MHz, Chloroform-d) δ 7.29 (s, 1H), 6.91 (d, J = 8.8 Hz, 1H), 6.09 (d, J = 6.1 Hz, 1H), 5.68 (t, J = 8.6 Hz, 1H), 5.27 (s, 1H), 5.23 (q, J = 7.8, 6.1 Hz, 1H), 5.02 (d, J = 11.1 Hz, 1H), 4.82 (d, J = 7.4 Hz, 1H), 4.38 (d, J = 10.0 Hz, 1H), 3.83 (dd, J = 10.9, 6.5 Hz, 2H), 3.11 (d, J = 2.7 Hz, 3H), 2.92 (s, 3H), 2.48 (t, J = 4.7 Hz, 1H), 2.35 (t, J = 7.7 Hz, 2H), 2.27 (dd, J = 11.4, 4.8 Hz, 1H), 2.18 (dt, J = 7.1, 3.5 Hz, 2H), 2.01 (s, 4H), 1.75 (s, 2H), 1.65 (t, J = 7.3 Hz, 3H), 1.54 (d, J = 7.3 Hz, 2H), 1.45 (t, J = 7.3 Hz, 4H), 1.27 (s, 14H), 1.17 (d, J = 6.4 Hz, 3H), 1.01 (dd, J = 12.2, 6.6 Hz, 3H), 0.94 (d, J = 6.5 Hz, 3H), 0.89 (d, J = 6.3 Hz, 3H), 0.81 (d, J = 6.5 Hz, 3H).

N-Boc pentapeptide **M4** (15.6 mg, 0.0225 mmol) was dissolved in 4M HCl-Dioxane at 0℃ for 1 h. The mixture was concentrated under reduced pressure. The residue was added DCM (3×30 mL) and concentrated to removed excess acid. The residue was added diazirine-alkyne modified acid (9.8 mg, 0.03375 mmol), BOP-Cl (8.6 mg, 0.03375 mmol), and DCM (1 mL). To the mixture solution was added TEA (0.0065 mL, 0.042 mmol) dropwise at 0℃ and stirred overnight. The mixture was concentrated and purified by silica column chromatography to get **Probe 4** (8.6 mg, 40.7% yield) as a clear semi-solid. ^1^H NMR (300 MHz, Chloroform-*d*) δ 7.29 (d, *J* = 5.5 Hz, 1H), 6.89 (d, *J* = 8.8 Hz, 1H), 6.09 (dd, *J* = 6.0, 1.6 Hz, 1H), 5.66 (dd, *J* = 10.0, 2.2 Hz, 1H), 5.29 – 5.18 (m, 2H), 5.06 – 4.99 (m, 1H), 4.95 (dd, *J* = 8.9, 3.3 Hz, 1H), 4.82 (dddd, *J* = 8.9, 6.9, 4.4, 1.9 Hz, 1H), 4.45 – 4.31 (m, 1H), 3.93 – 3.73 (m, 2H), 3.55 (d, *J* = 11.0 Hz, 1H), 3.11 (s, 3H), 2.92 (s, 3H), 2.48 (ddd, *J* = 14.5, 10.1, 4.6 Hz, 1H), 2.35 (dd, *J* = 8.7, 6.7 Hz, 2H), 2.27 (d, *J* = 6.0 Hz, 1H), 2.17 (dt, *J* = 6.8, 3.4 Hz, 1H), 2.01 (s, 3H), 2.00 – 1.94 (m, 2H), 1.65 (t, *J* = 7.4 Hz, 4H), 1.47 (dd, *J* = 7.7, 3.9 Hz, 5H), 1.39 – 1.20 (m, 19H), 1.17 (d, *J* = 6.5 Hz, 3H), 1.08 (d, *J* = 7.6 Hz, 1H), 0.99 (d, *J* = 6.5 Hz, 3H), 0.94 (d, *J* = 6.6 Hz, 3H), 0.89 (d, *J* = 6.4 Hz, 3H), 0.81 (d, *J* = 6.6 Hz, 3H). ^13^C NMR (75 MHz, Chloroform-*d*) δ 174.62, 174.18, 171.21, 169.85, 168.89, 154.16, 125.33, 83.46, 71.88, 68.89, 68.56, 60.40, 59.19, 58.60, 58.12, 56.96, 54.09, 51.96, 36.59, 36.29, 33.85, 32.82, 31.81, 30.76, 30.44, 29.69, 29.43, 29.36, 29.17, 28.46, 27.15, 25.11, 24.92, 23.82, 23.15, 22.74, 21.93, 21.06, 18.84, 18.37, 17.95, 17.41, 16.93, 14.20. HRMS (ESI) calcd for C_46_H_73_N_7_NaO_9_ [M ^+^ Na^+^] 890.5345; found, 890.5362.

N-Boc pentapeptide **M4** (7.8 mg, 0.0112 mmol) was dissolved in 4M HCl-Dioxane at 0℃ for 1 h. The mixture was concentrated under reduced pressure. The residue was added DCM (3×10 mL) and concentrated to removed excess acid. The residue was added biotin-derived acid (13 mg), BOP-Cl (4.3 mg, 0.017 mmol), and DCM (1 mL). To the mixture solution was added TEA (0.0033 mL, 0.021 mmol) dropwise at 0℃ and stirred overnight. The mixture was concentrated and purified by HPLC to get **Probe 5** (3.6 mg, 23.6% yield) as a faint yellow oil. HRMS (ESI) calcd for C_66_H_113_N_9_NaO_18_S [M ^+^ Na^+^] 1374.7814; found, 1374.7814.

Supplementary Figures

**
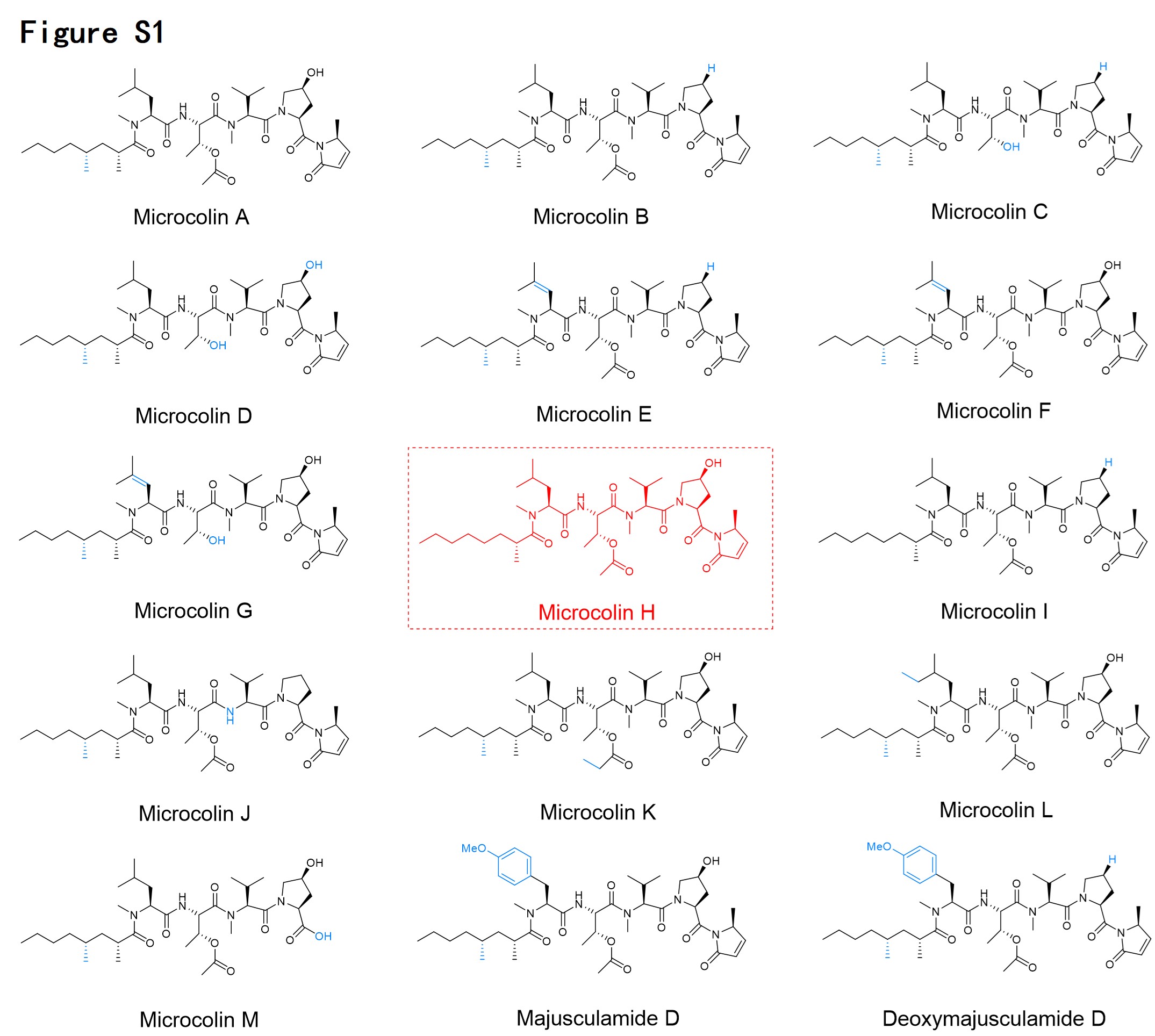
**

**Fig. S1 Natural products of microcolins and majusculamide D**

**
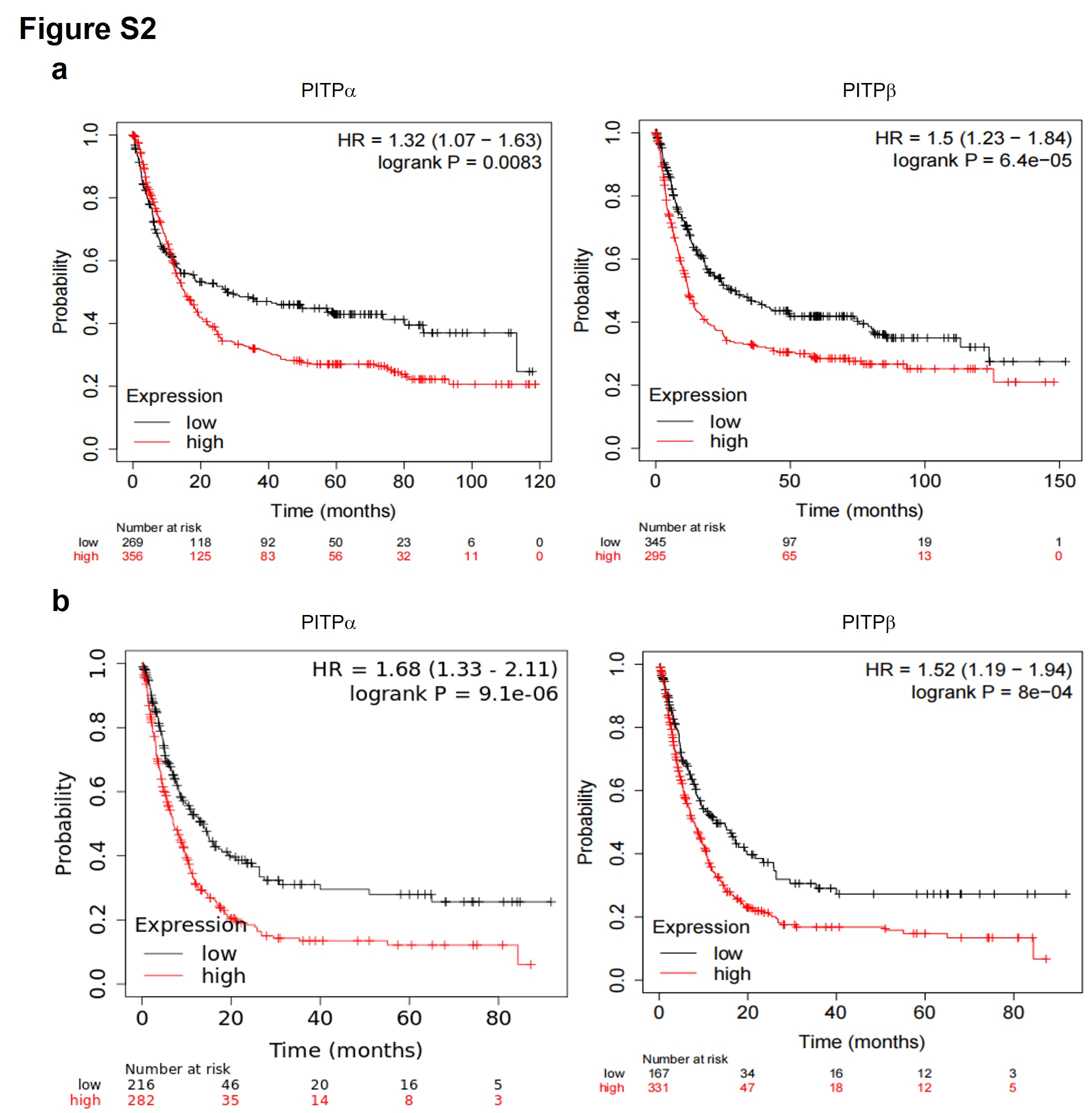
**

**Fig. S2 PITPα/β were highly expressed in tumour tissues and were associated with a poor prognosis in gastric cancer.** Statistical analysis of PITPα/β expressions and patients’ first progression survival (PFS) (**a**) and patients’ post progression survival (PPS) (**b**) using Kaplan-Meier plotter.


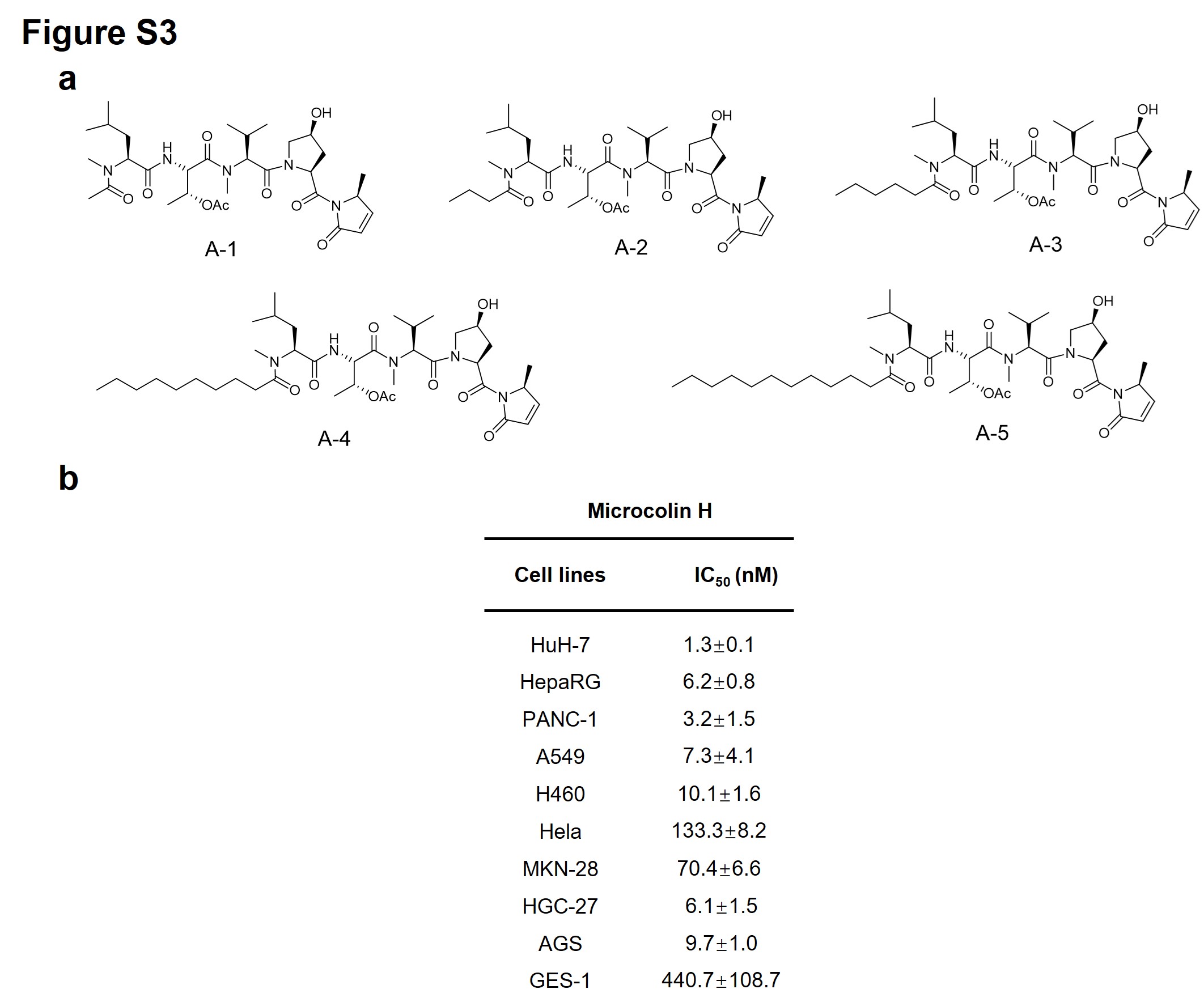


**Fig. S3 Analogs and anti-tumour activity of microcolin H. a** Analogs structures of microcolin H. **b** The effect of microcolin H on various tumour cells viability using CCK8 assay. Cells were treated with various doses of microcolin H for 48h. Tumour cell lines were from liver cancer (HuH-7 and HepaRG), pancreatic cancer (PANC-1), lung cancer (A549 and H460), cervical cancer (Hela), gastric cancer (MKN-28, HGC-27, and AGS), and gastric mucosa epithelial cell line GES-1. Data was presented as mean ± SD.


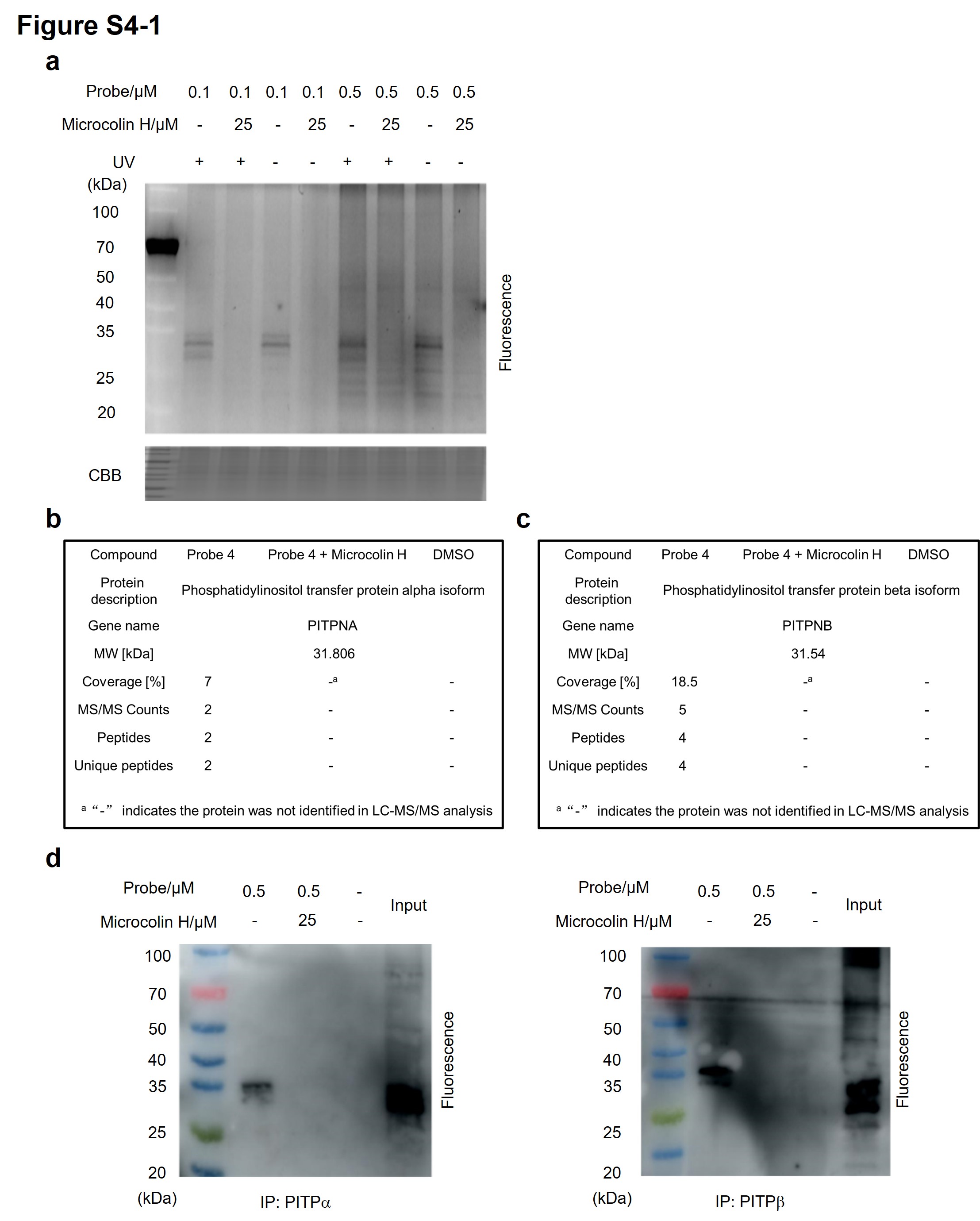


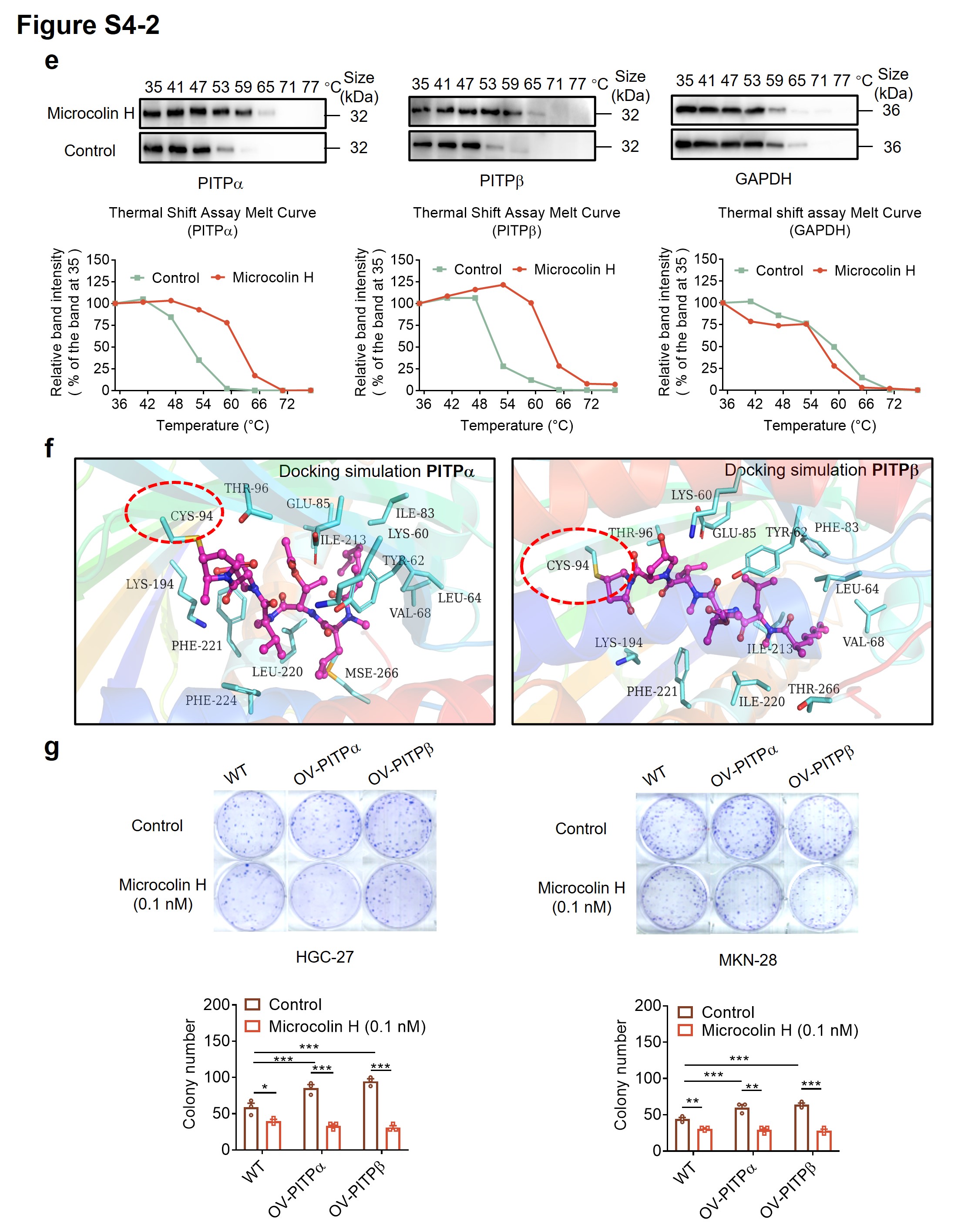


**Fig. S4 Microcolin H directly targets PITPα and PITPβ**. **a** Competitive Labeling with microcolin H in HGC-27 cells with or without UV. **b, c** The LC-MS/MS analysis revealed that phosphatidylinositol transfer protein alpha/ beta isoform (PITPα/β) might be a target of microcolin H. **d** Western blot analysis to determine competitive binding of probe and microcolin H to PITPα and PITPβ. **e** Thermal shift assay was used to evaluate the binding between microcolin H and PITPα/β in thermodynamic levels. **f** Docking simulation predicts the microcolin H-binding pocket in PITPα/β and Cys94 as the potential target residue. **g** Wild type (WT) and PITPα/β over expression HGC-27/MKN-28 cells (OV-PITPα/β) were treated with microcolin H for 48 h. Then, the cells were cultured for 9 d, and the colonies were counted, n=3 per group. Data are presented as the mean ± SEM. *P<0.05, **P<0.01, ***P<0.001, vs. control group.


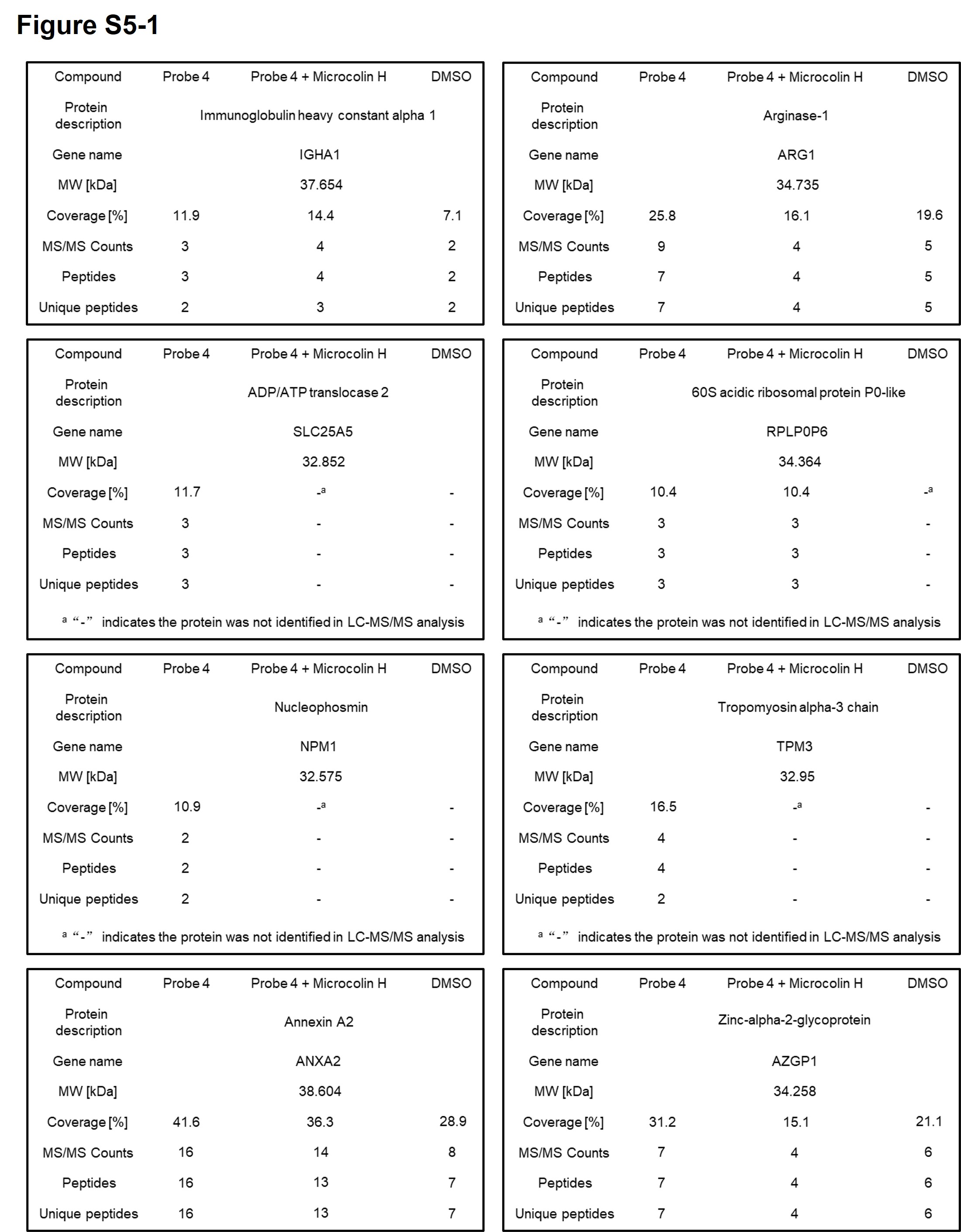


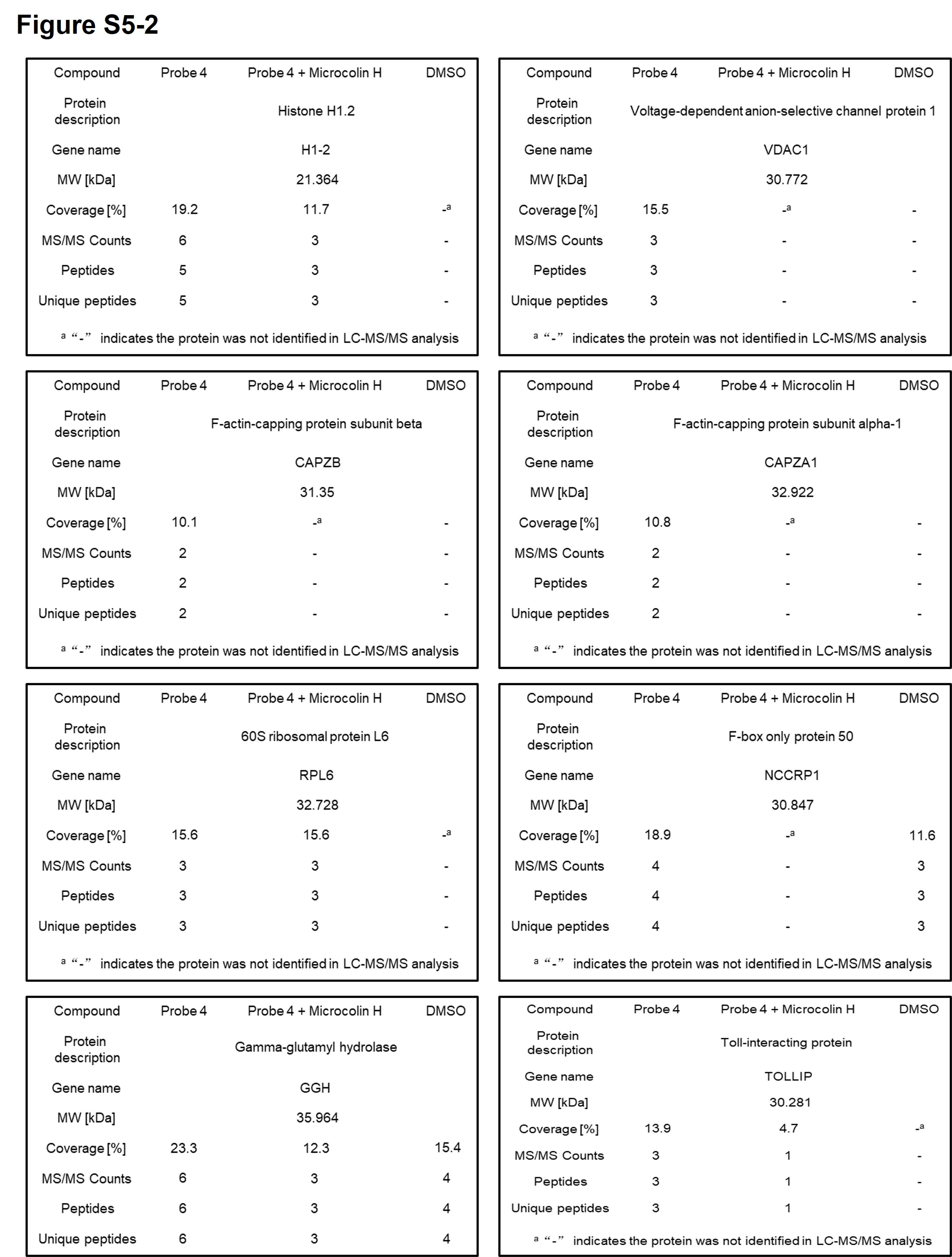


**Fig. S5 The other candidate targets of Microcolin H.**


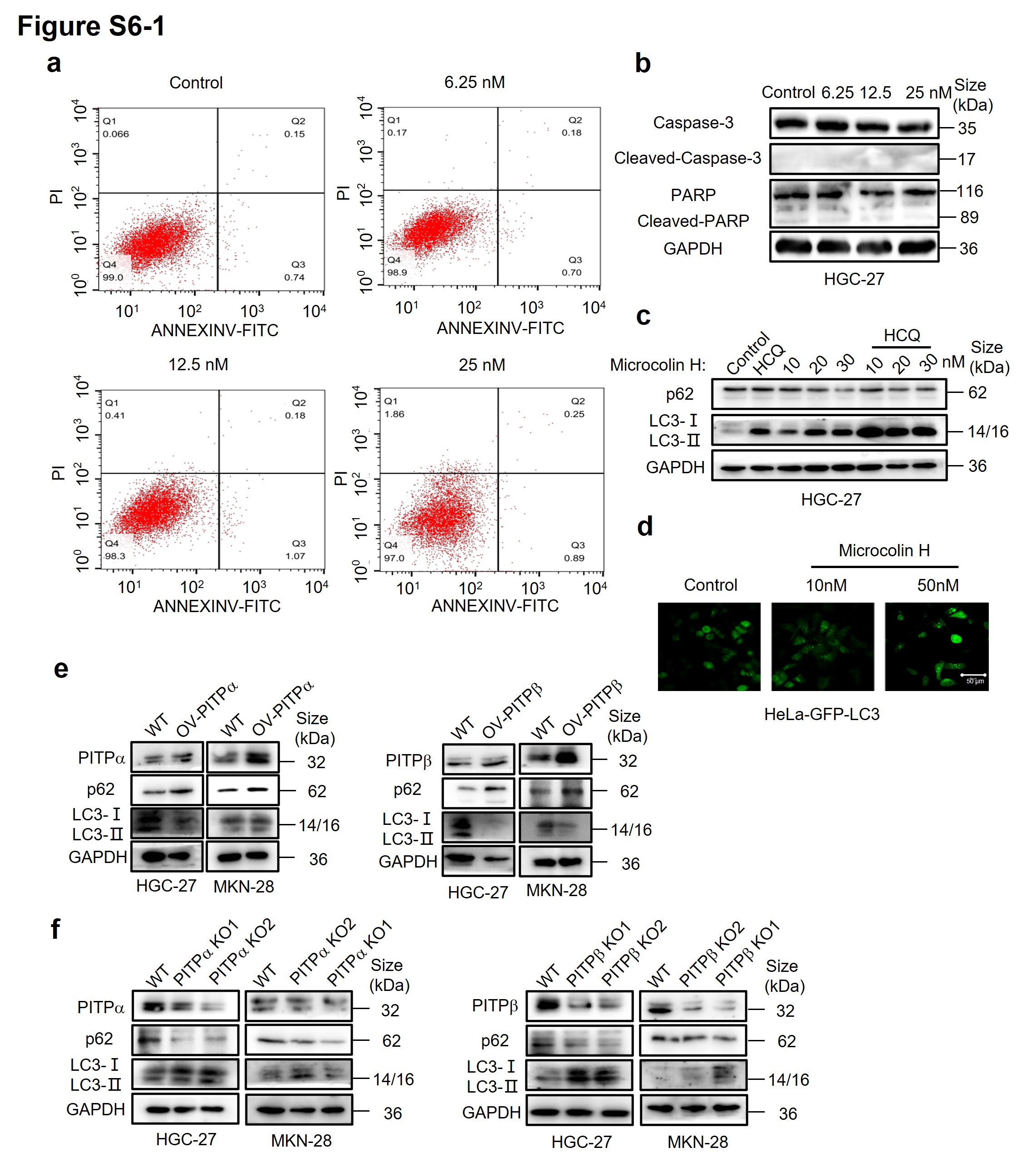


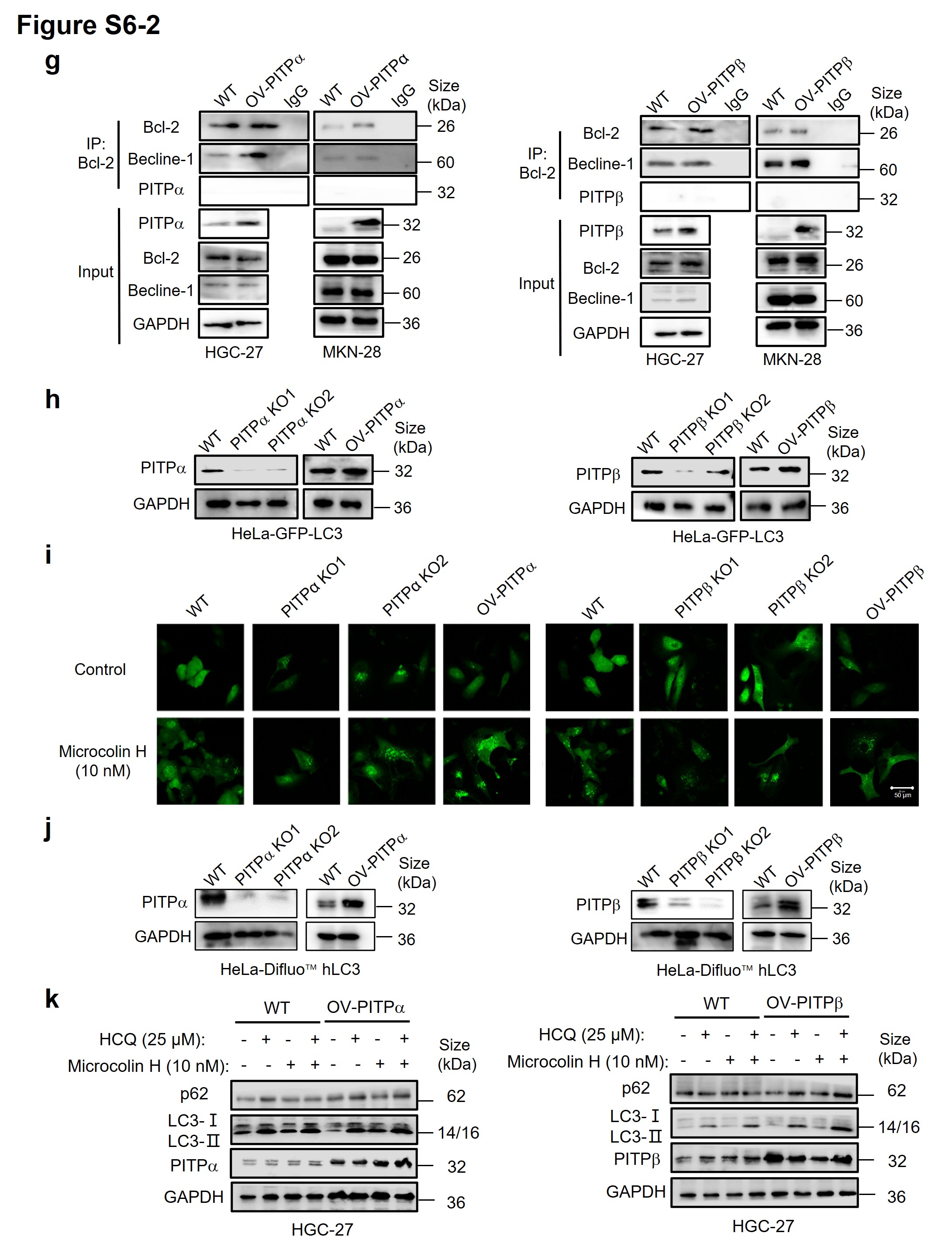


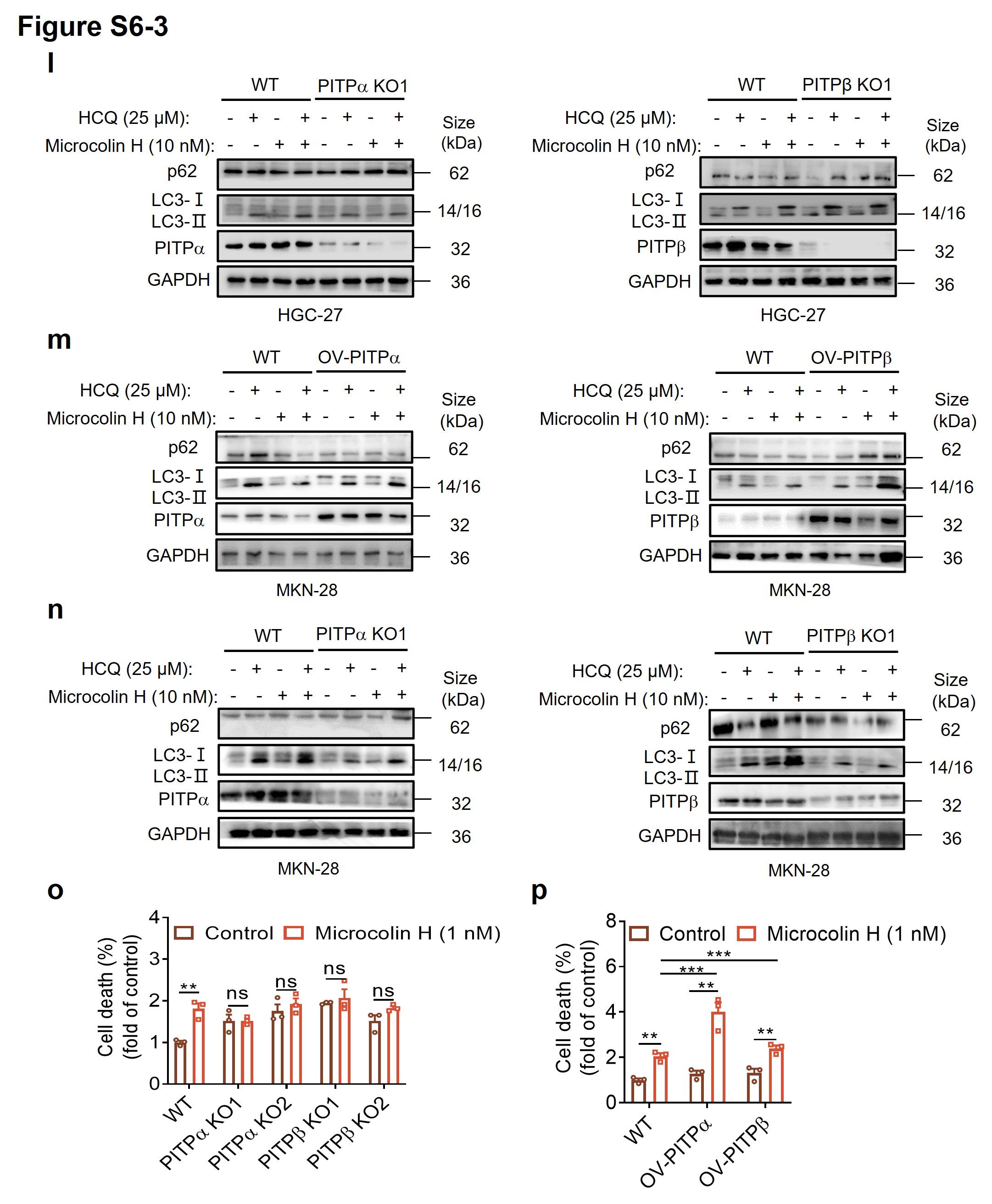


**Fig. S6 Microcolin H induces autophagy-dependent cell death via PITPα/β. a** PI/Annexin V staining for cell apoptosis in HGC-27 cells treated with microcolin H for 24 h. **b** Total and cleave Caspase 3 and PARP expression in HGC-27 treated with microcolin H for 48 h. **c** Western blot assay of LC3 expression in HGC-27 cells treated with microcolin H and HCQ for 6 h. **d** Autophagosomes were observed in Hela-GFP-LC3 cells treated with different concentrations of microcolin H by confoal microscope. **e**. Western blot analysis of PITPα/β; p62 and LC3I/II expression for wild type (WT) and PITPα/β over-expression (OV-PITPα/β) in HGC-27 and MKN-28 cells. **f** Western blot assay of PITPα/β; p62 and LC3I/II expression for wild type (WT) and PITPα/β knockout (PITPα/β KO) in HGC-27 and MKN-28 cells. **g** Co-IP assay with an antibody against Bcl-2 to detect the Bcl-2/Beclin-1 interaction in OV-PITPα/β GC cells. **h** Western blot assay of PITPα/β expressions for wild type (WT) and PITPα/β knockout (PITPα/β KO) and overexpression (OV-PITPα/β) in HeLa-GFP-LC3 cells. **i** Autophagosomes were observed in Hela-GFP-LC3 cells transfected with plasmids (PITPα/β KO/ OV-PITPα/β) with or without microcolin H treatment by confoal microscope. **j** Western blot assay for PITPα/β expressions for wild type (WT) and PITPα/β knockout (PITPα/β KO) and overexpression (OV-PITPα/β) in H HeLa-Difluo™ hLC3 cells. **k, l** Western blot analysis of LC3 and p62 expression after HGC-27 and transfected HGC-27 cells had been treated with microcolin H and HCQ. **m, n** LC3I/II and p62 expression in MKN-28 cells transfected with plasmids (PITPα/β KO and OV-PITPα/β) with or without microcolin H (10μM) and HCQ (25 μM) treatment for 6 h by western blot assay. **o, p** Trypan blue staining for cell death in MKN-28 cells transfected with plasmids (PITPα/β KO/ OV-PITPα/β) with or without microcolin H treatment for 48 h. Data are presented as the mean ± SEM. *P<0.05, **P<0.01, ***P<0.001, vs. control group.


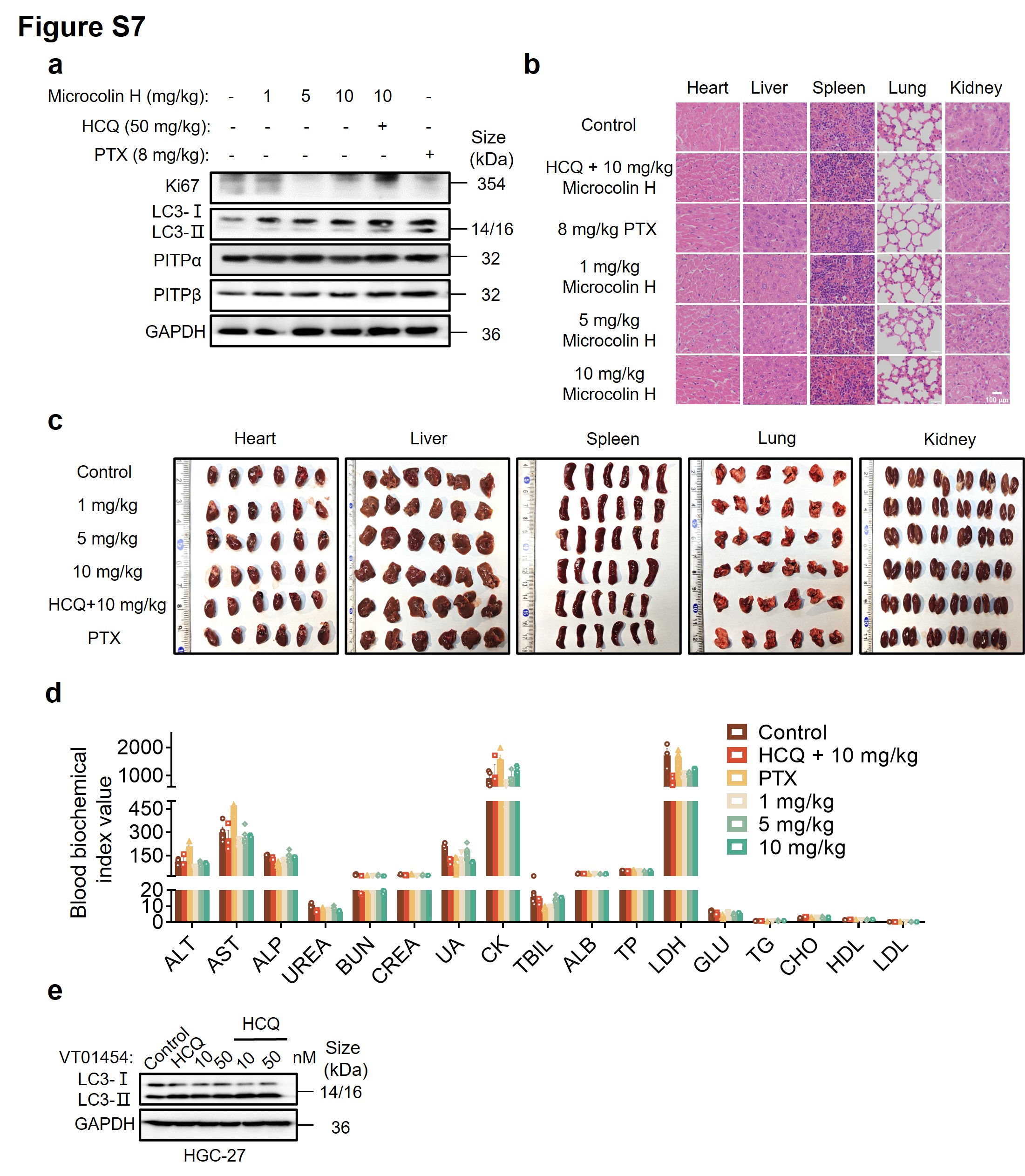


**Fig. S7 Microcolin H has strong antitumour activity in vivo and is well tolerated in mice. a** Western blot assay for Ki67, LC3I/II and PITPα/β expression in tumour xenografts. **b** HE staining for tissue mice organs structure damage in control and treatment groups. **c** The appearance of mice organs in control and treatment groups, n=6 per group. **d** Blood biochemical markers from control and treated Balb/c-nu/nu mice. The parameters included alanine aminotransferase (ALT, U/L),

aspartate aminotransferase (AST, U/L), alkaline phosphatase (ALP, U/L), urea (UREA, mmol/L), blood urea nitrogen (BUN, mg/dl), creatinine (CREA, μmol/L), uric acid (UA, μmol/L), creatine kinase (CK, U/L), total bilirubin (TBIL, μmol/L), albumin (ALB, g/L), total protein (TP, g/L), lactate dehydrogenase (LDH, U/L), glucose (GLU, mmol/L), triglycerides (TG, mmol/L), total cholesterol (CHO, mmol/L), high density lipoprotein (HDL, mmol/L) and low density lipoprotein (LDL, mmol/L). **e** LC3I/II expression in HGC-27 cells treated with VT01454 and HCQ (25μM) for 6 h by western blot assay. Data are presented as the mean ± SEM.


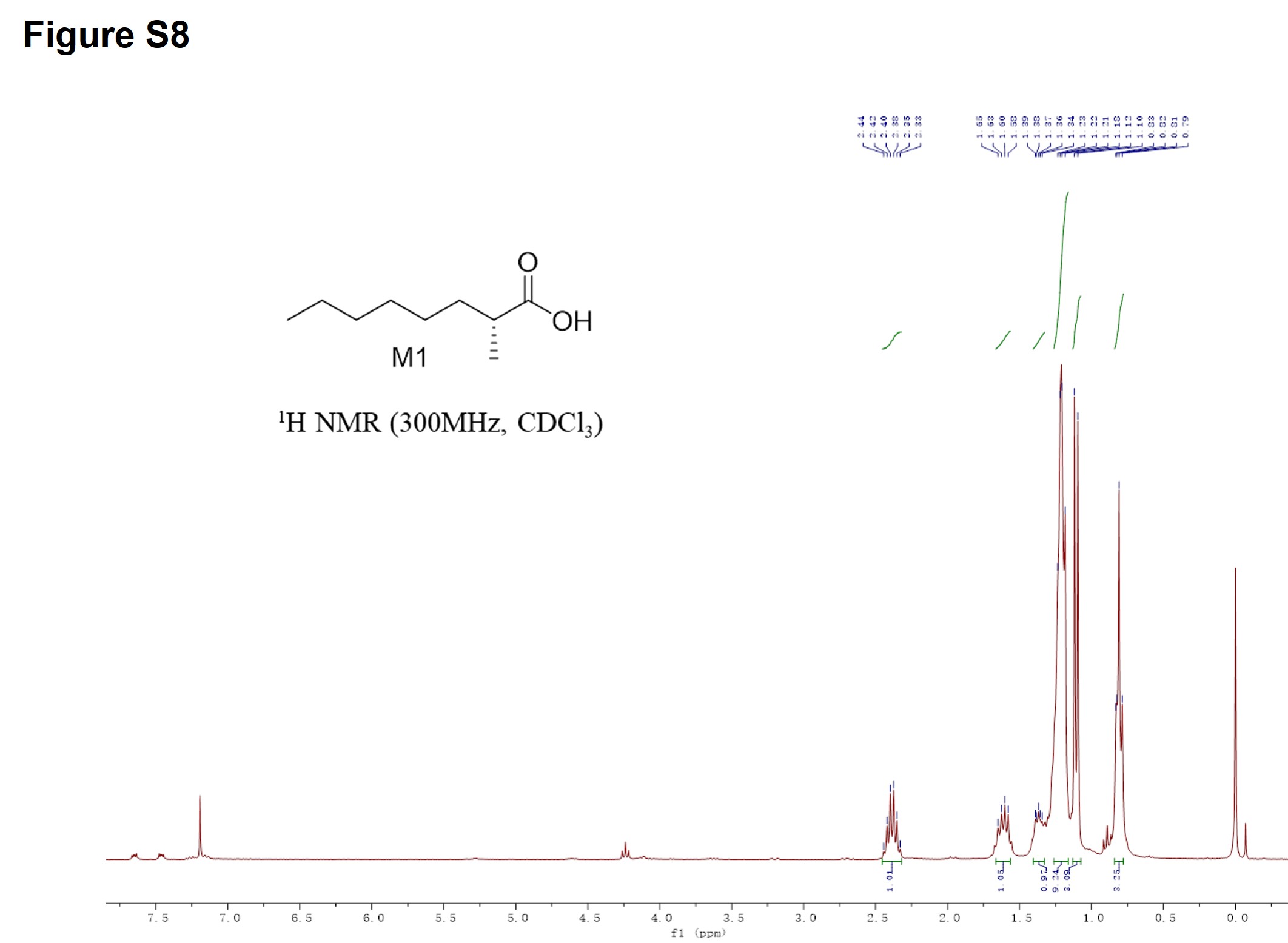


**Fig. S8** ^1^H NMR spectrum of compound **M1**.


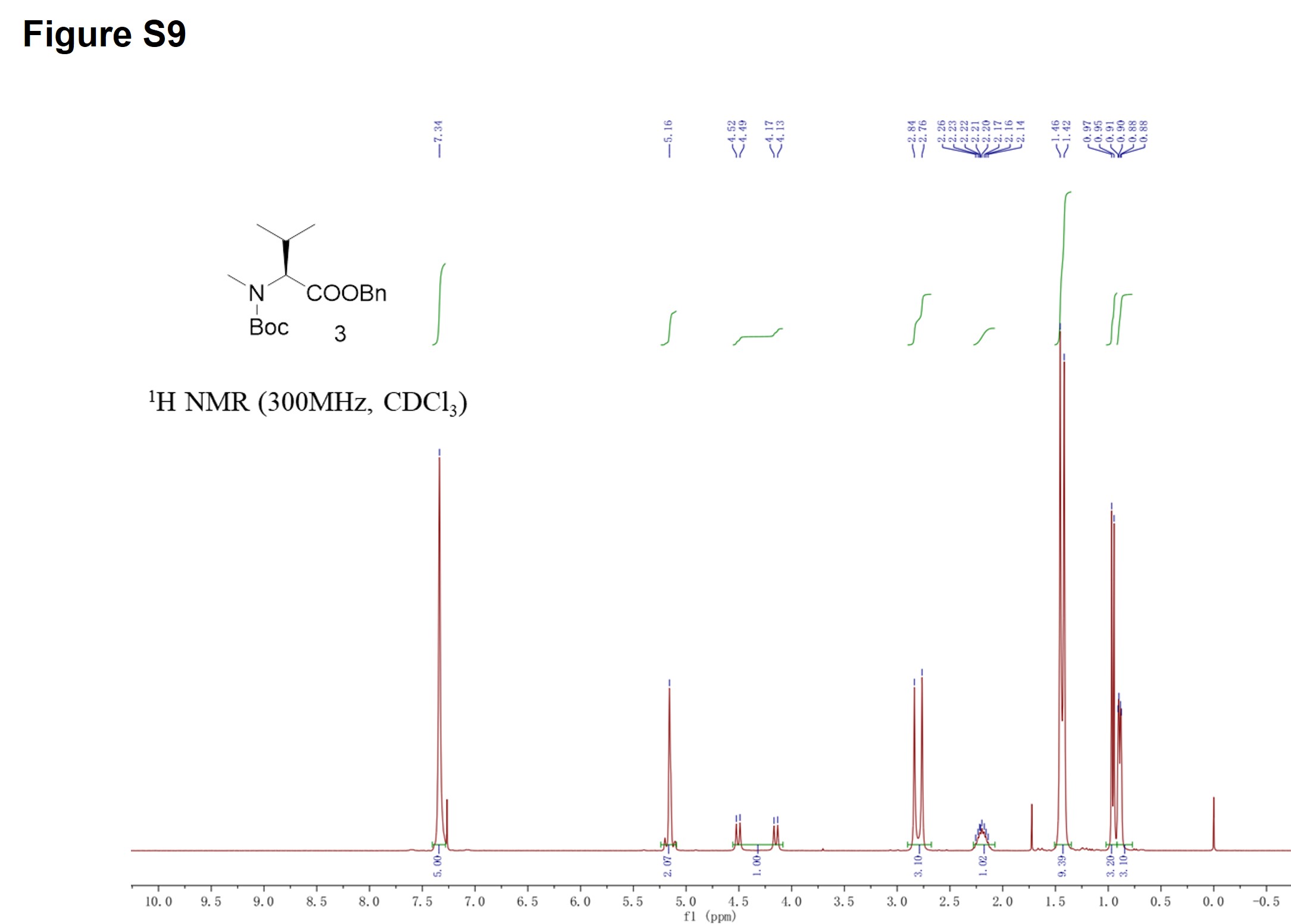


**Fig. S9** ^1^H NMR spectrum of compound **3**.


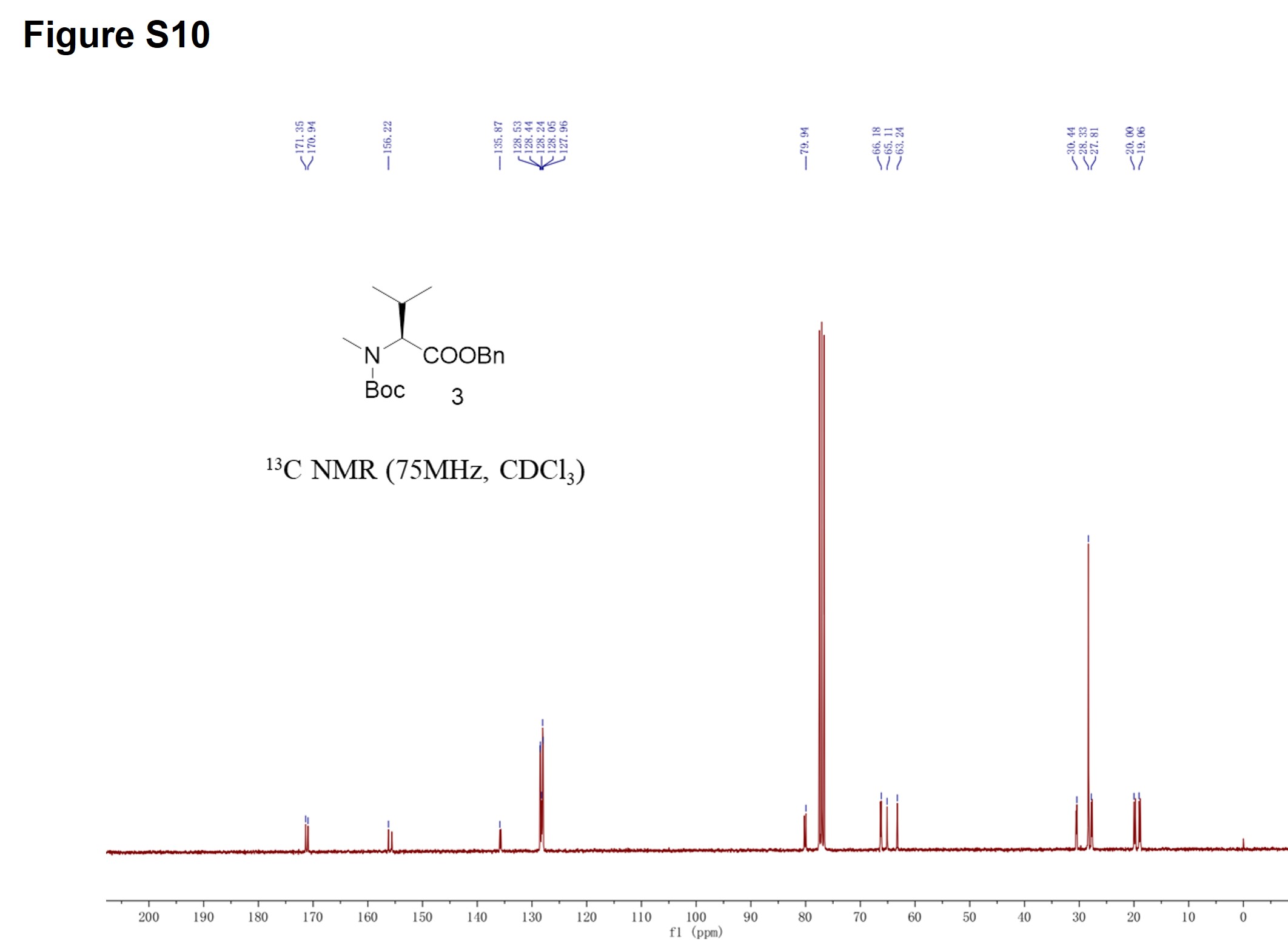


**Fig. S10** ^13^C NMR spectrum of compound **3**.

**
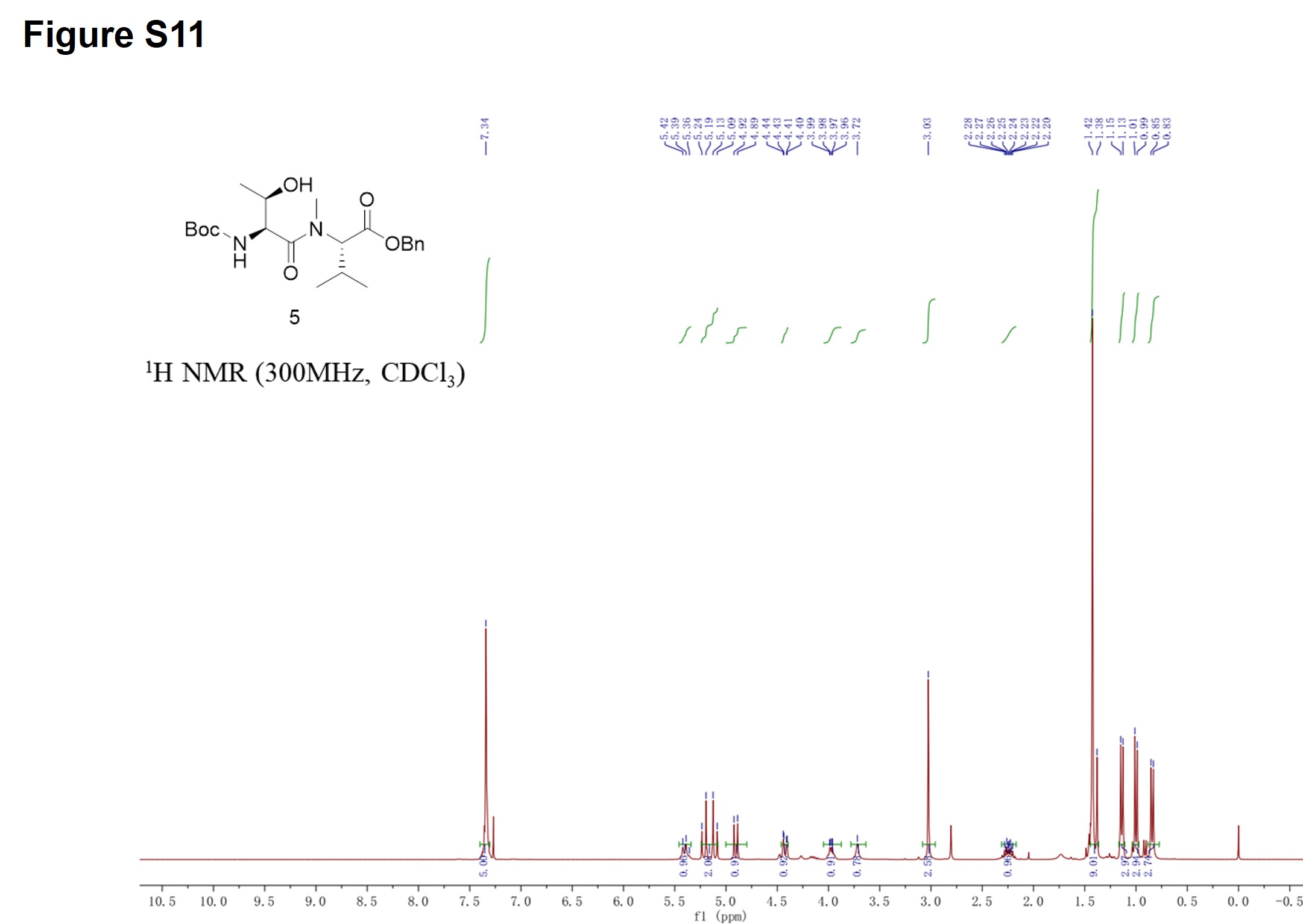
**

**Fig. S11** ^1^H NMR spectrum of compound **5**.


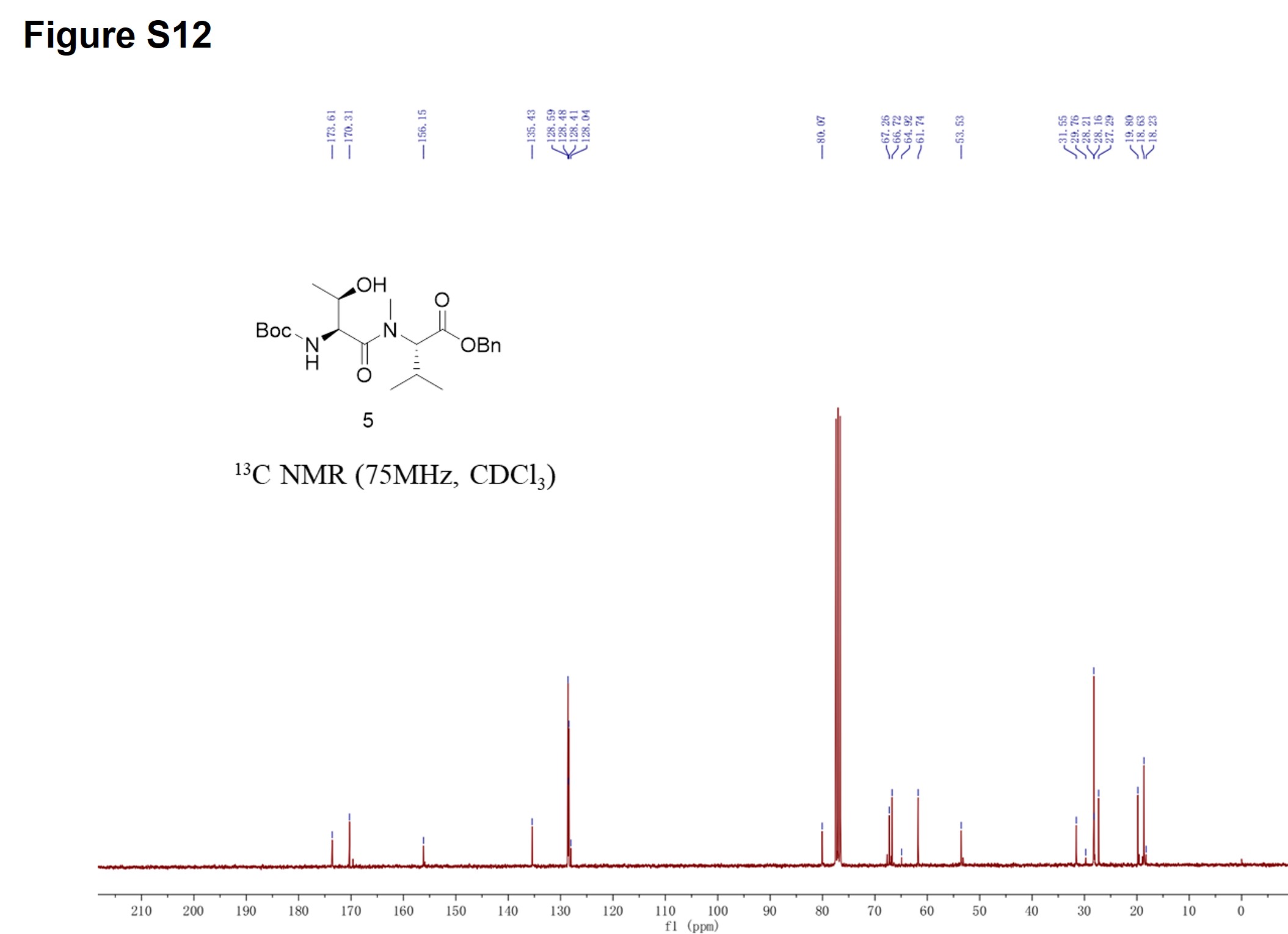


**Fig. S12** ^13^C NMR spectrum of compound **5**.


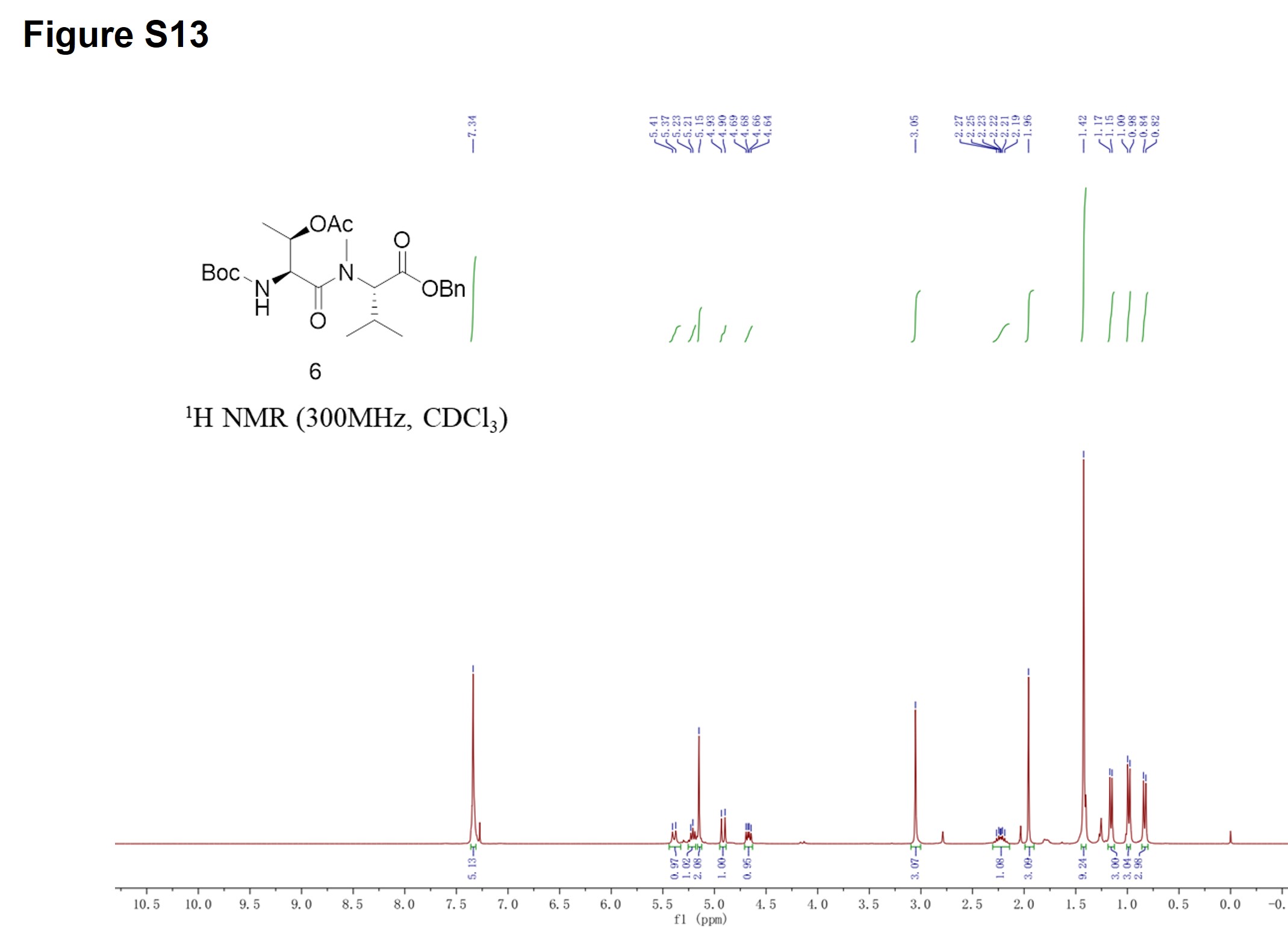


**Fig. S13** ^1^H NMR spectrum of compound **6**.


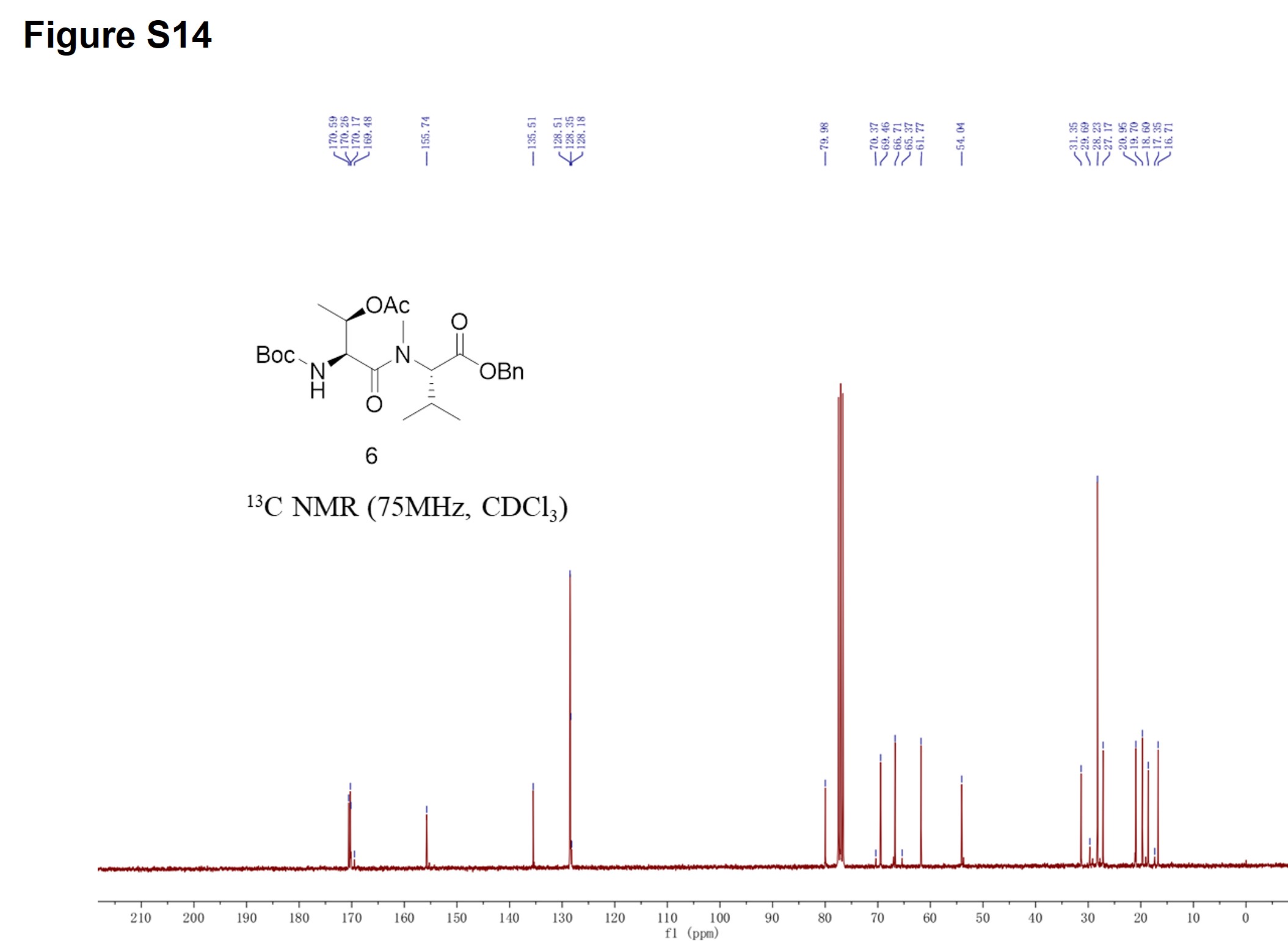


**Fig. S14** ^13^C NMR spectrum of compound **6**.


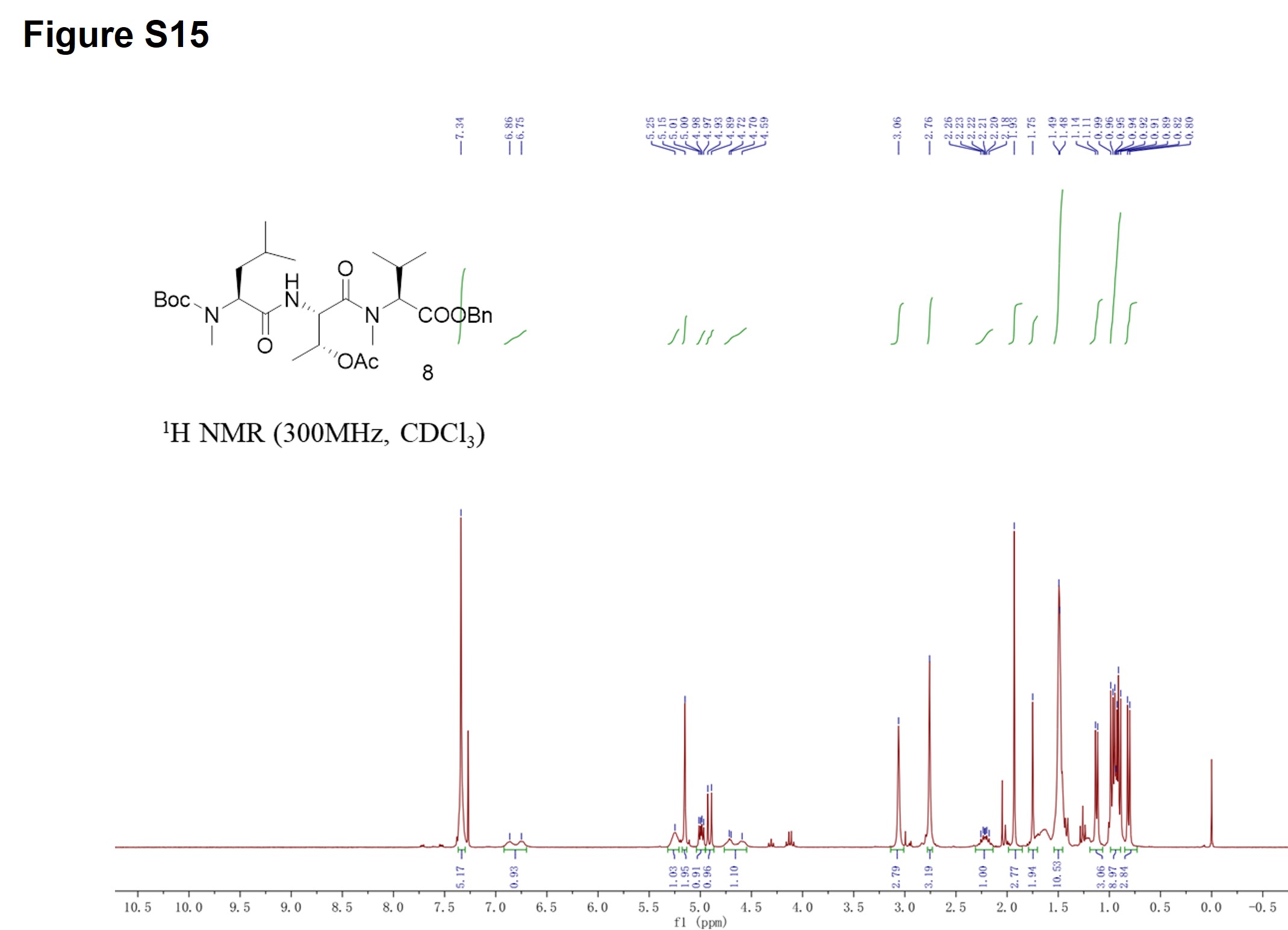


**Fig. S15** ^1^H NMR spectrum of compound **8**.

**
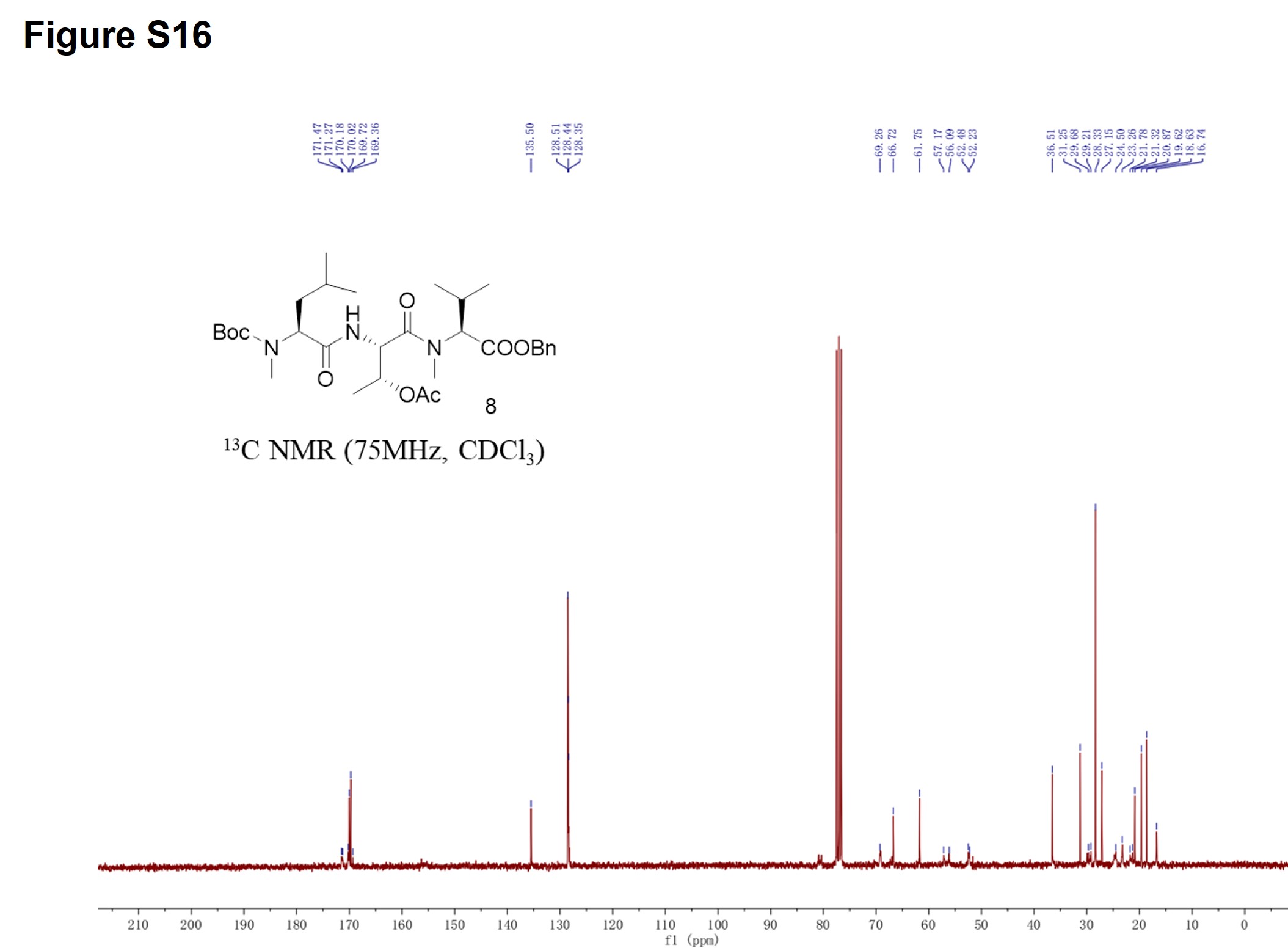
**

**Fig. S16** ^13^C NMR spectrum of compound **8**.

**
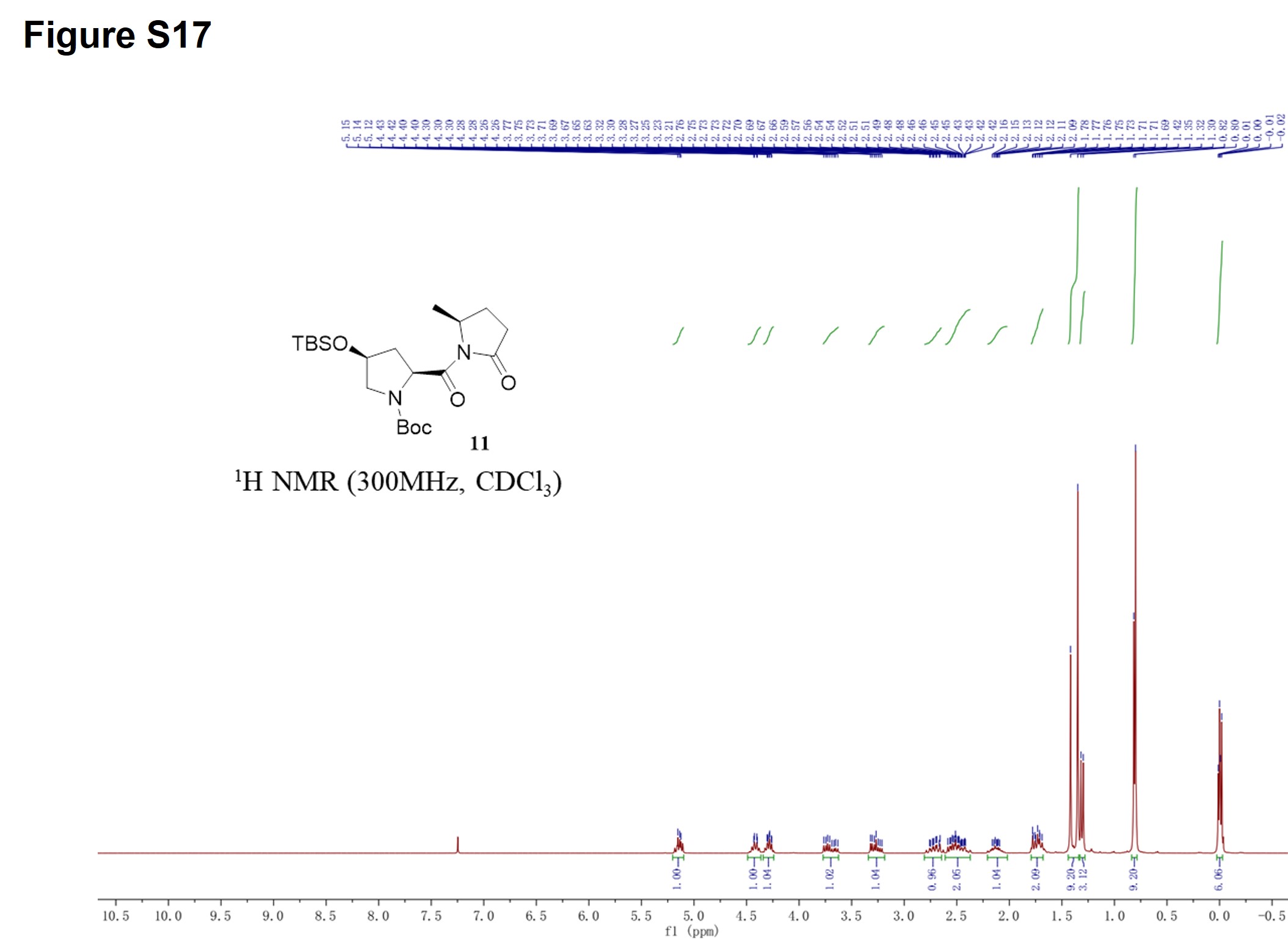
**

**Fig. S17** ^1^H NMR spectrum of compound **11**.

**
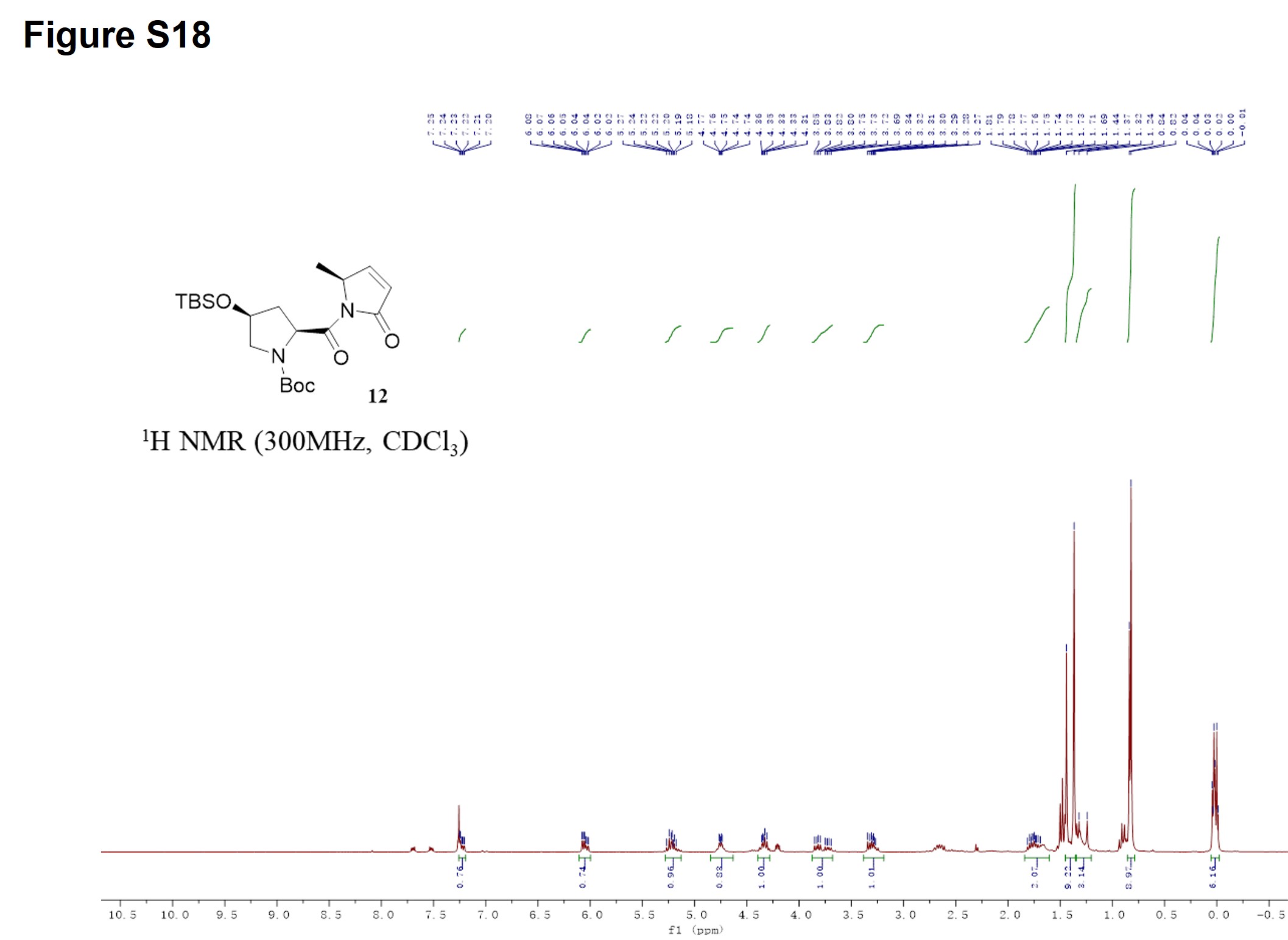
**

**Fig. S18** ^1^H NMR spectrum of compound **12**.

**
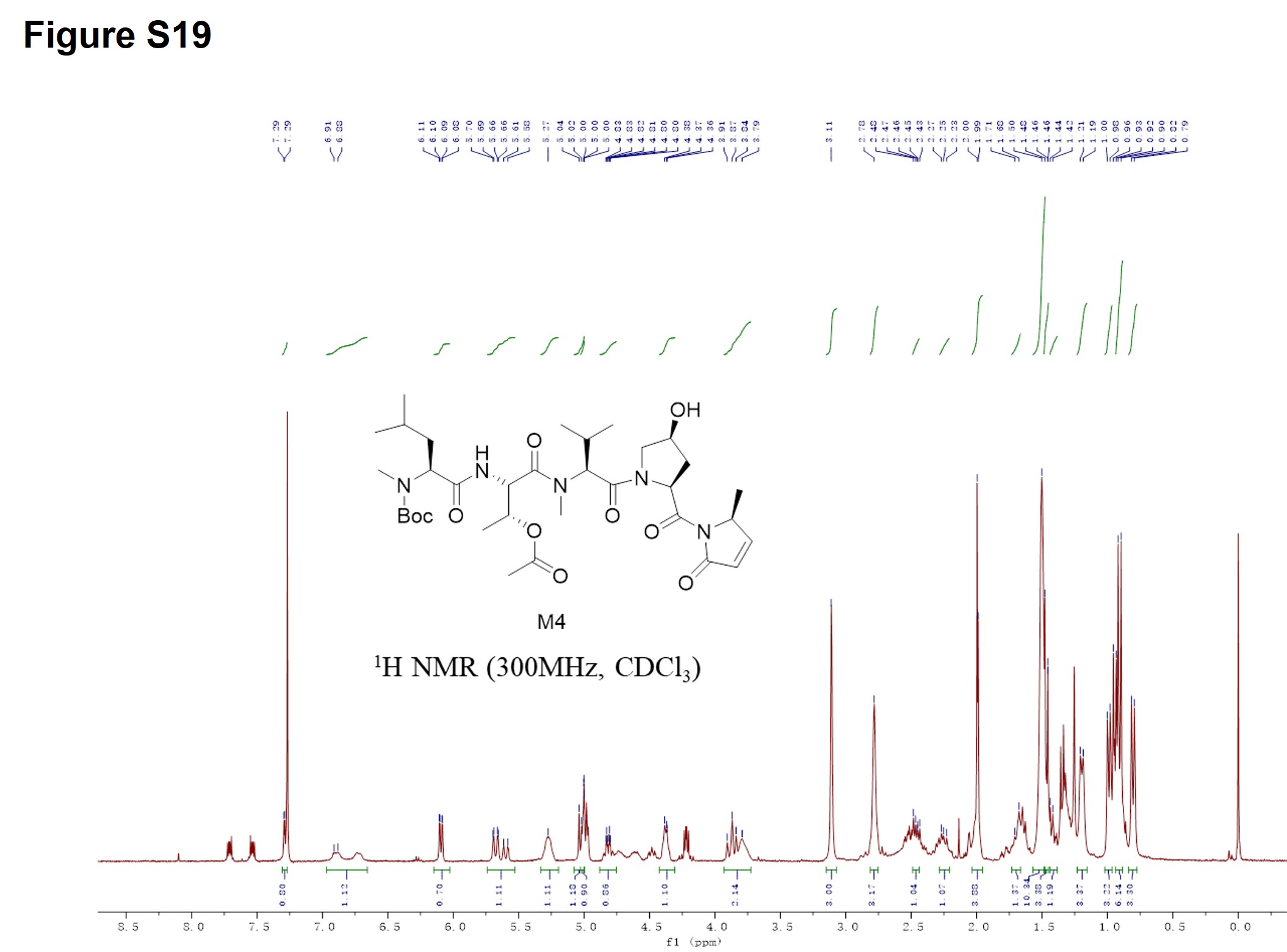
**

**Fig. S19** ^1^H NMR spectrum of compound **M4**.

**
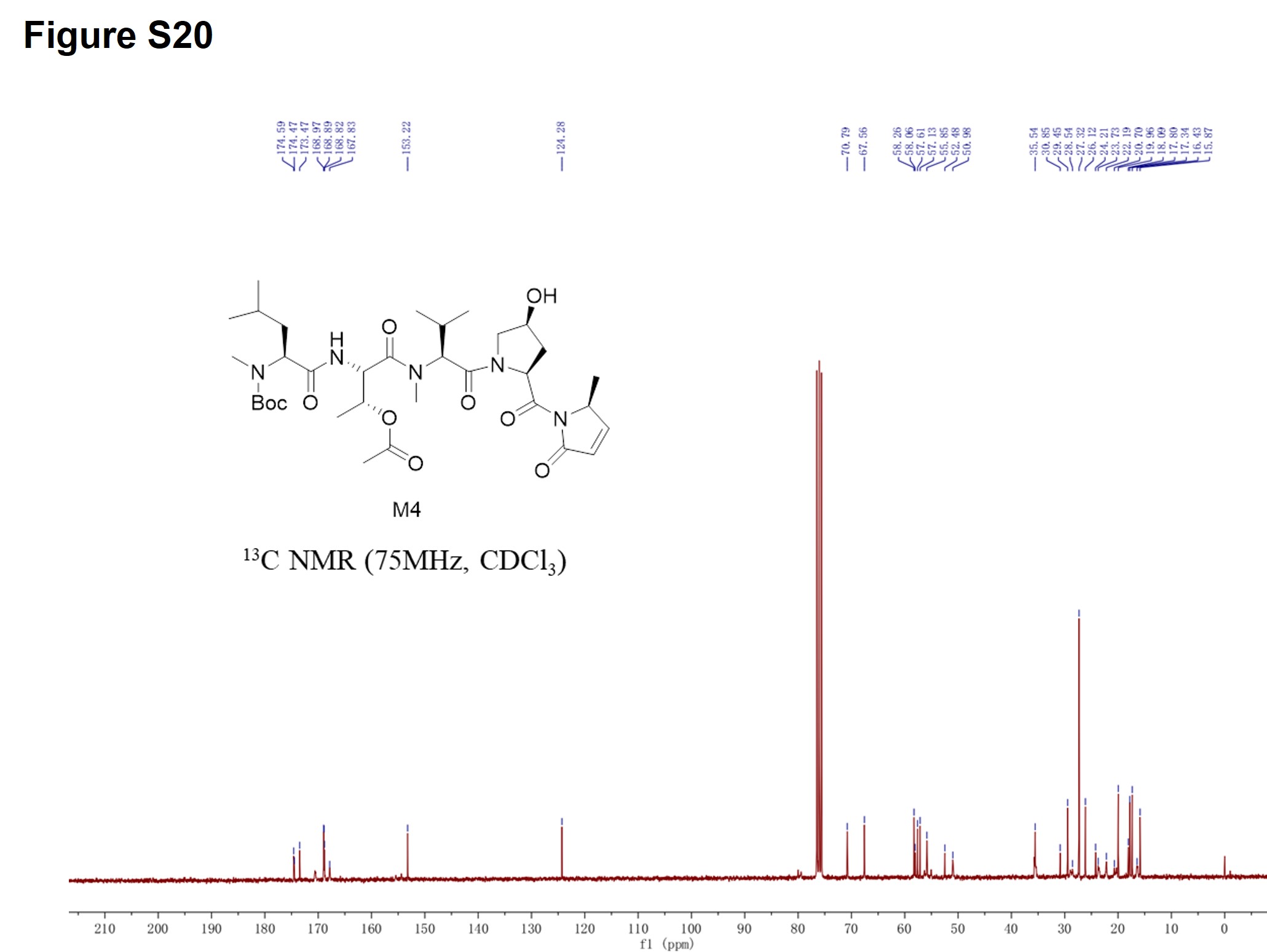
**

**Fig. S20** ^13^C NMR spectrum of compound **M4**.

**
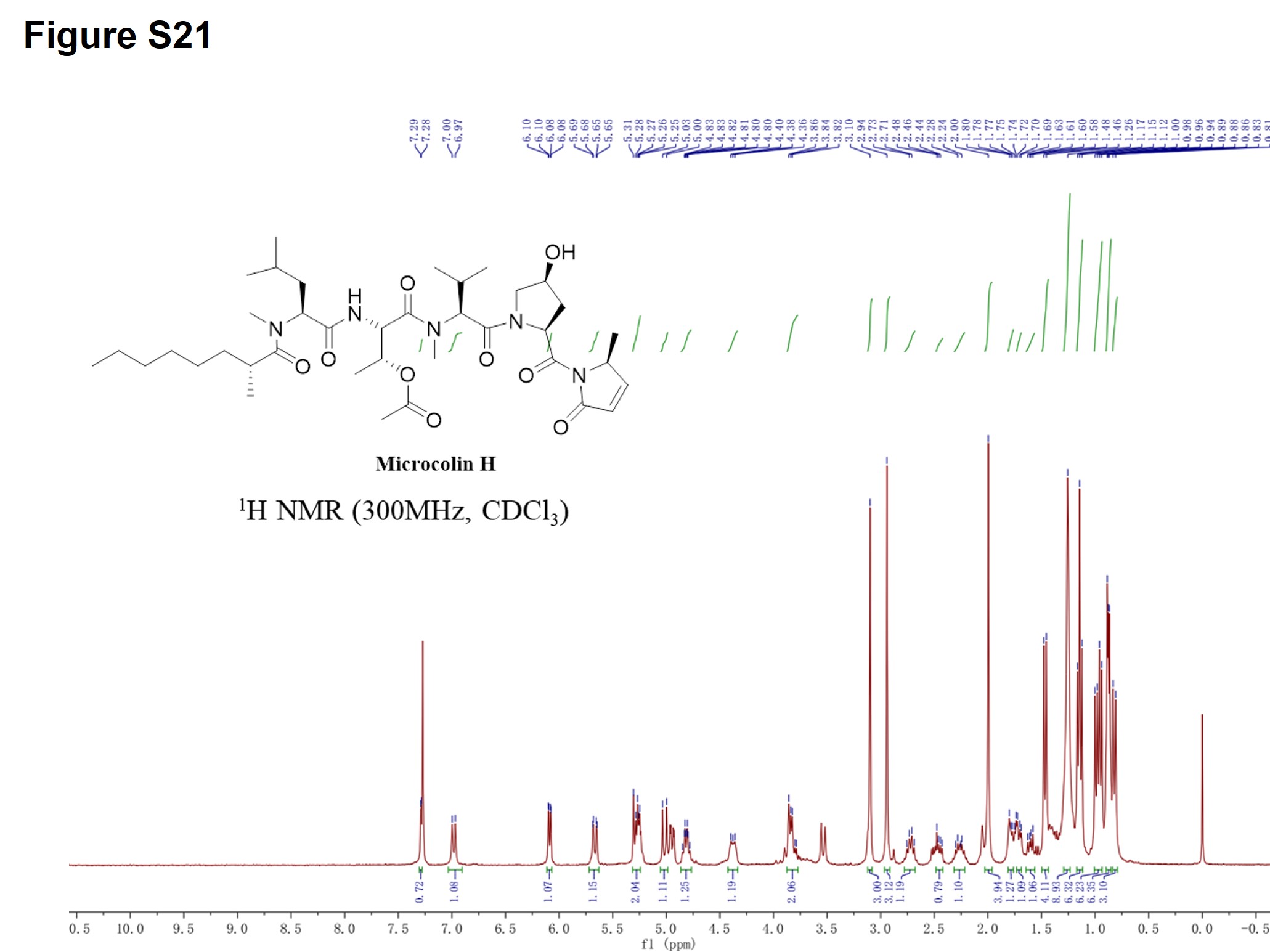
**

**Fig. S21** ^1^H NMR spectrum of compound **Microcolin H**.

**
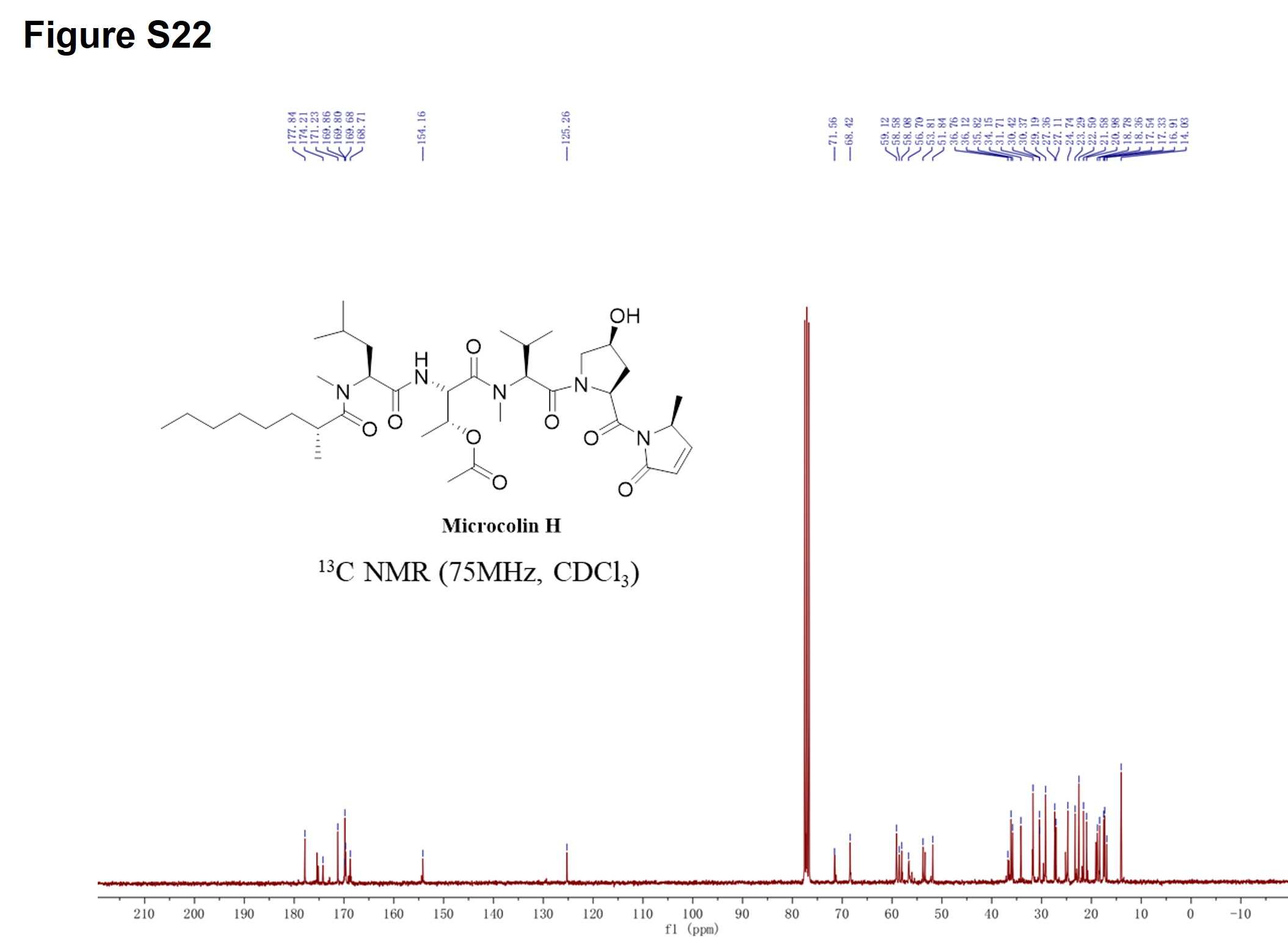
**

**Fig. S22** ^13^C NMR spectrum of compound **Microcolin H**.

**
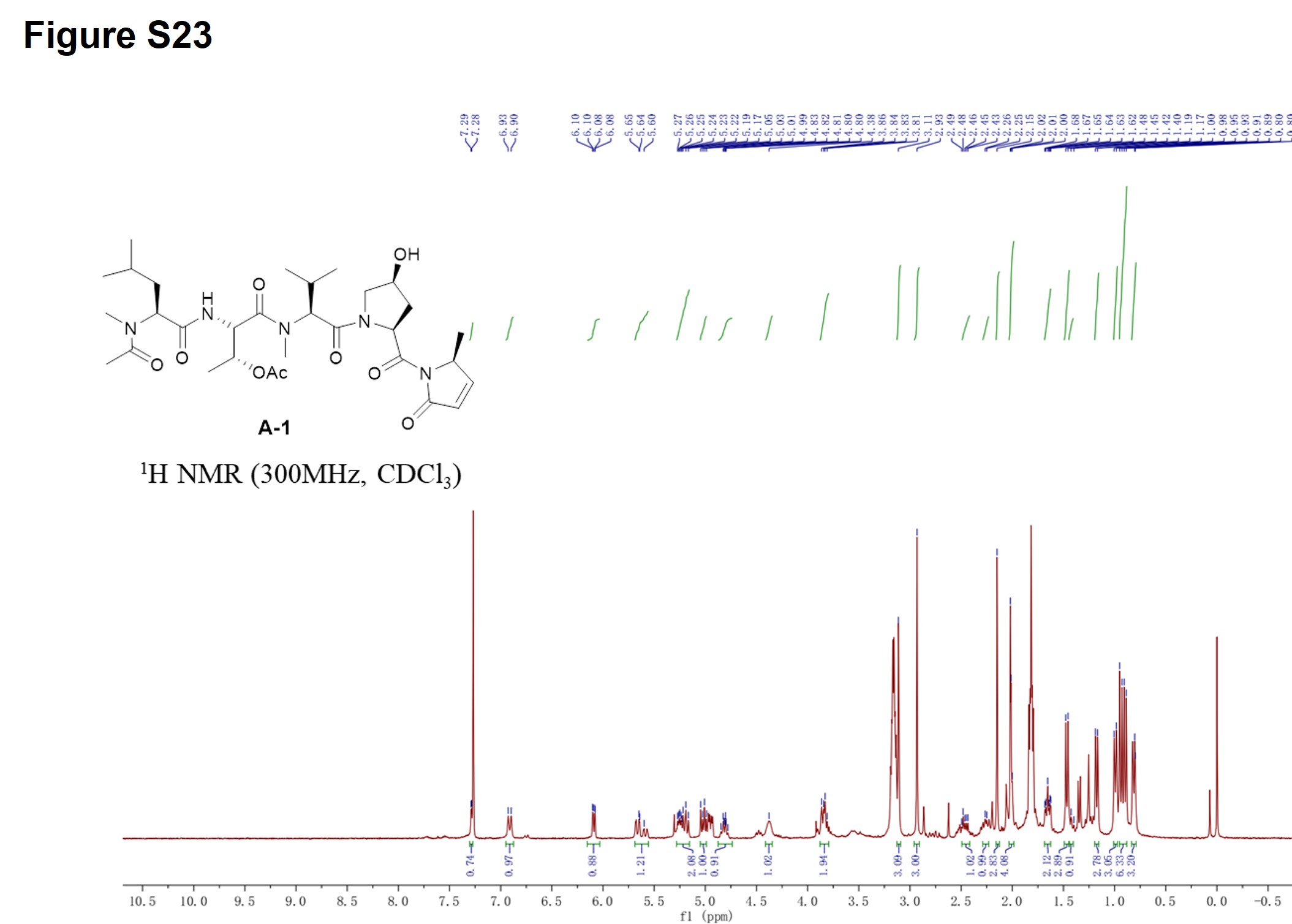
**

**Fig. S23** ^1^H NMR spectrum of compound **A-1**.

**
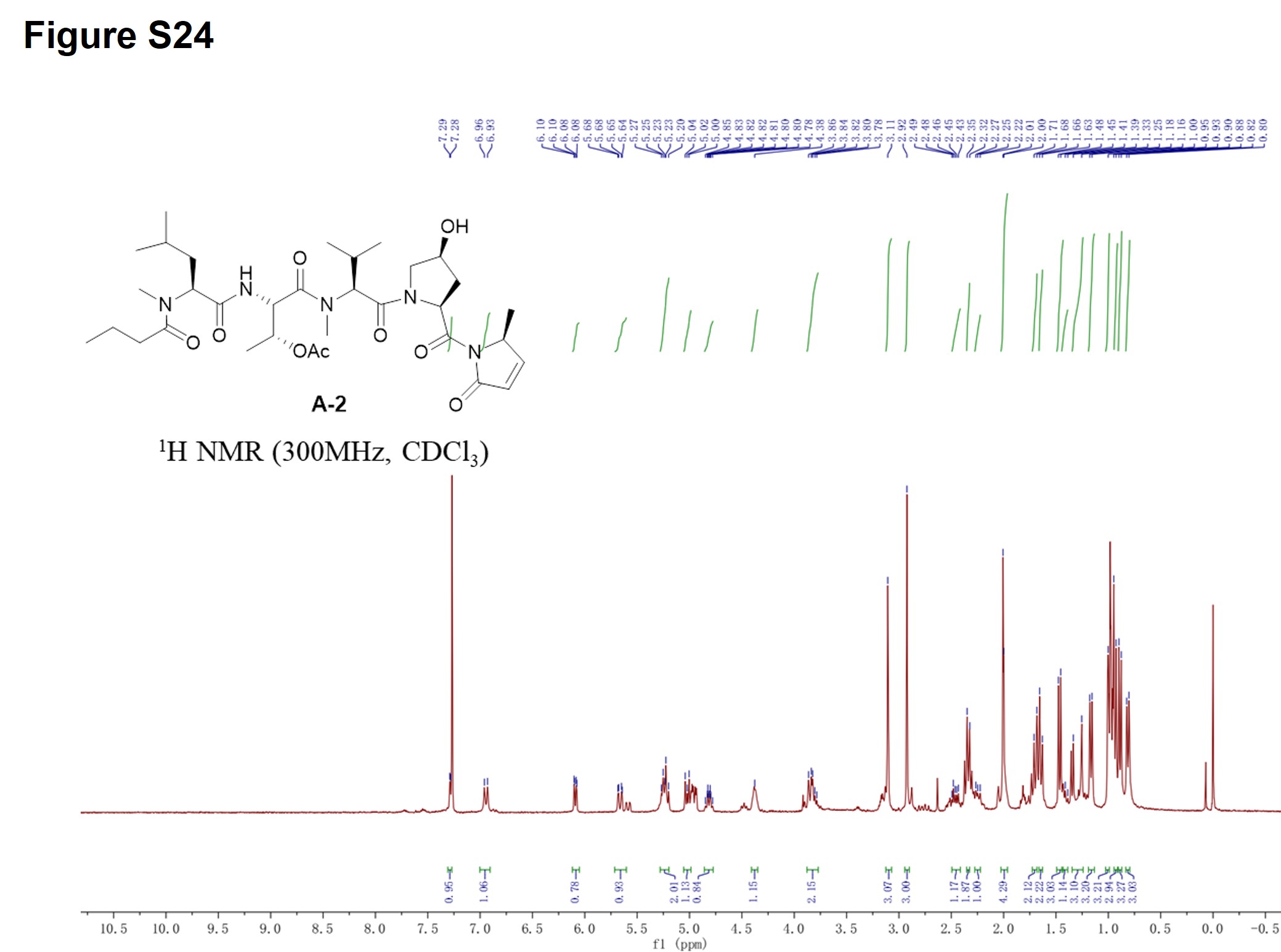
**

**Fig. S24** ^1^H NMR spectrum of compound **A-2**.

**
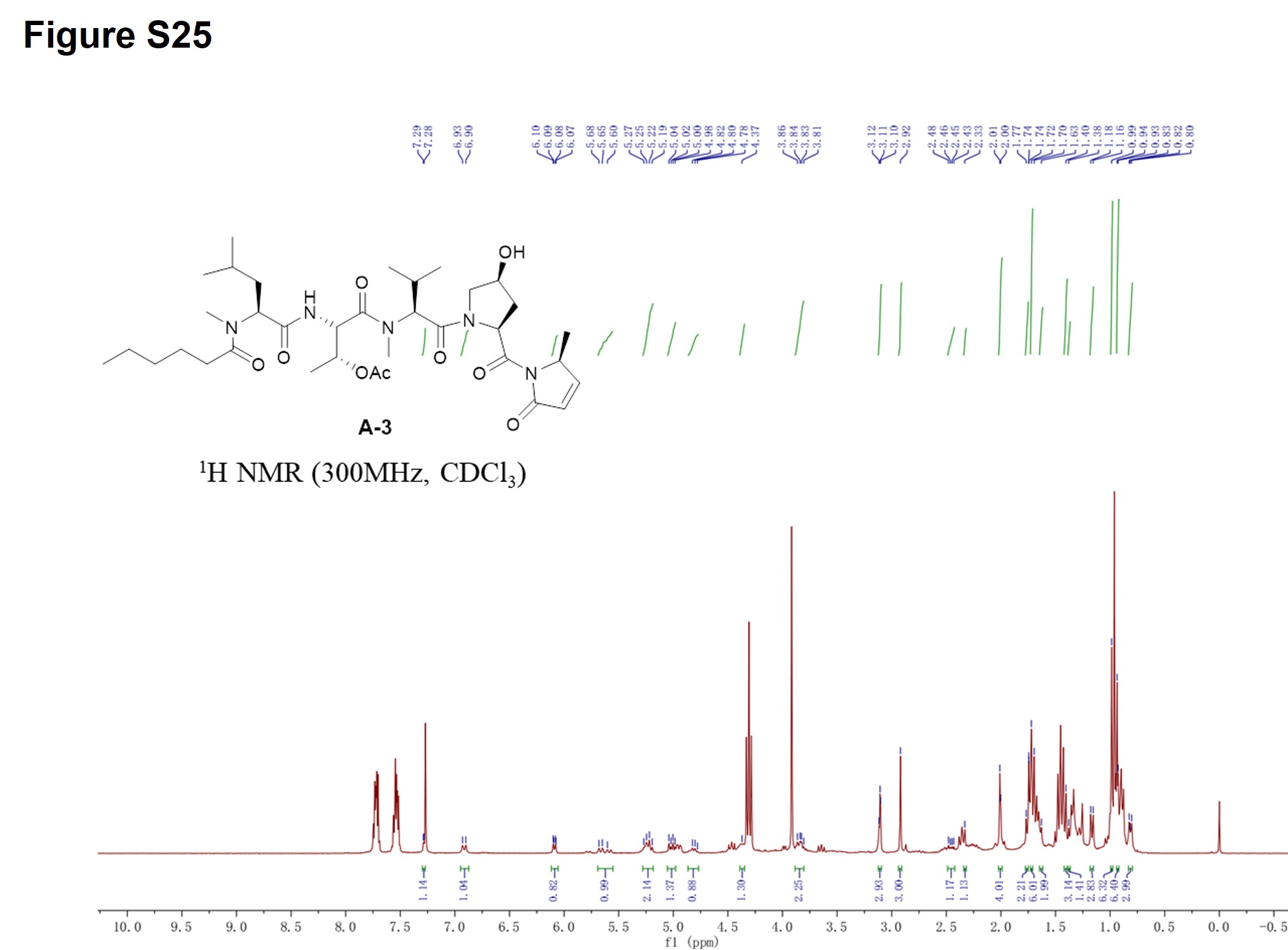
**

**Fig. S25** ^1^H NMR spectrum of compound **A-3**.

**
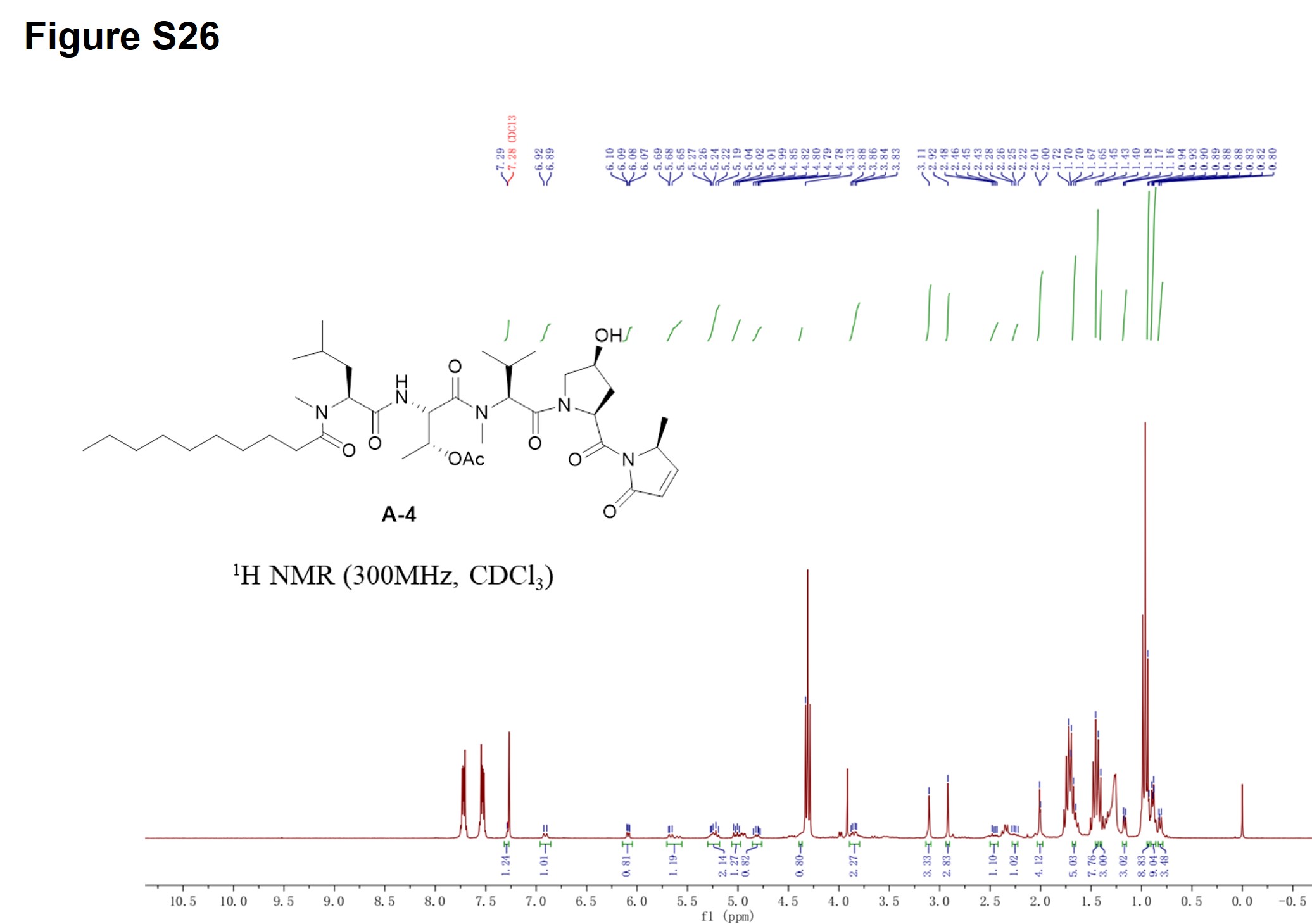
**

**Fig. S26** ^1^H NMR spectrum of compound **A-4**.

**
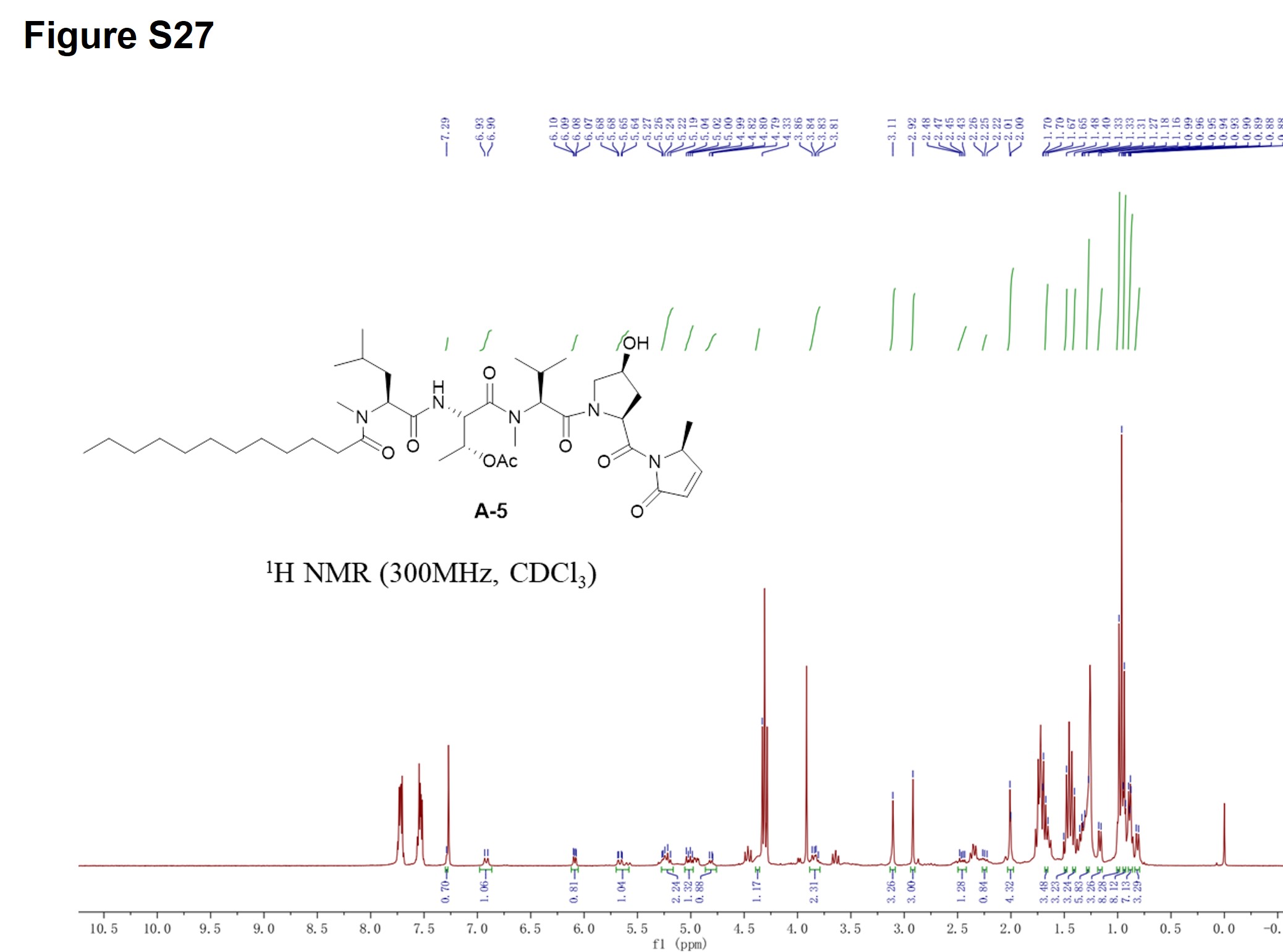
**

**Fig. S27** ^1^H NMR spectrum of compound **A-5**.

**
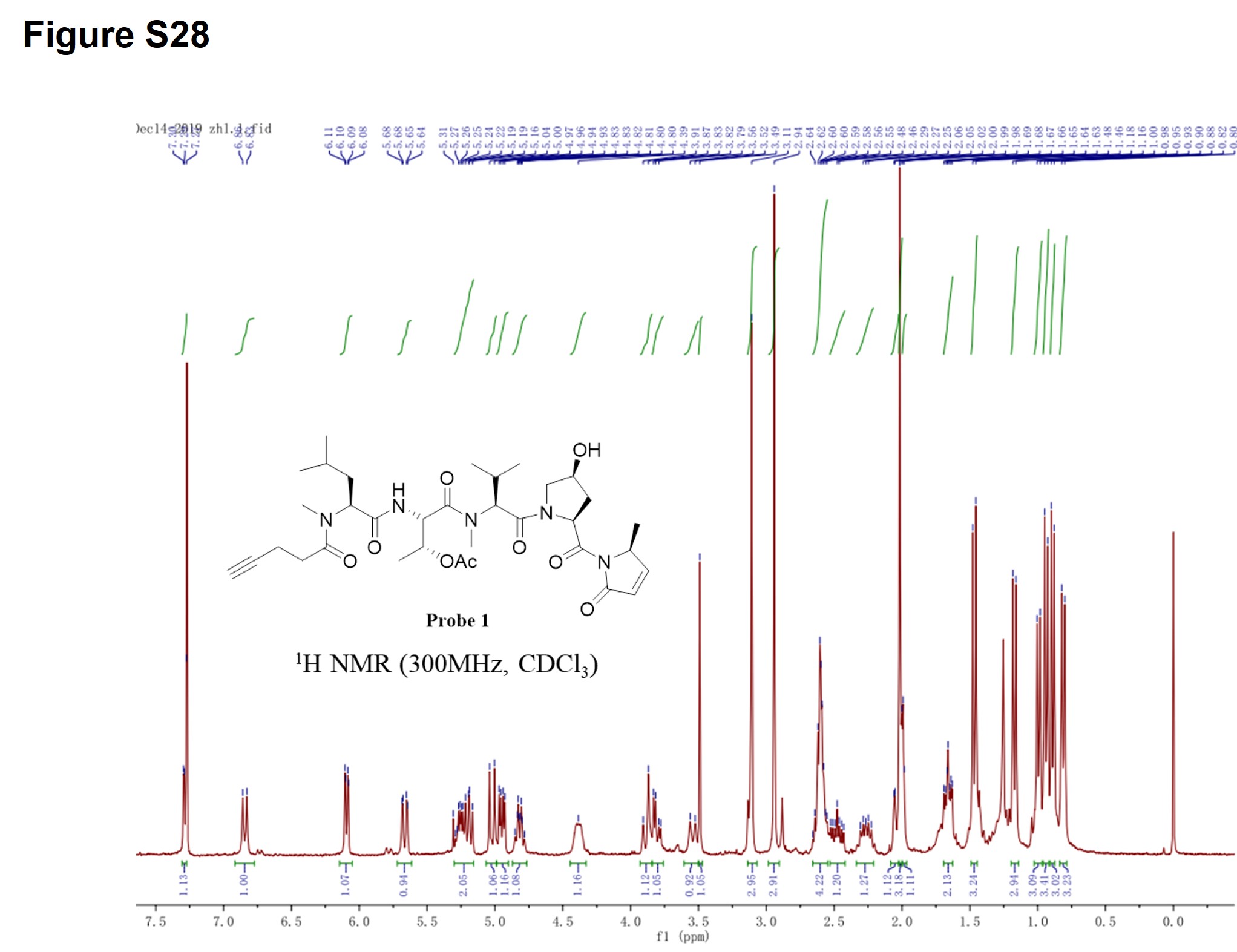
**

**Fig. S28** ^1^H NMR spectrum of compound **Probe 1**.

**
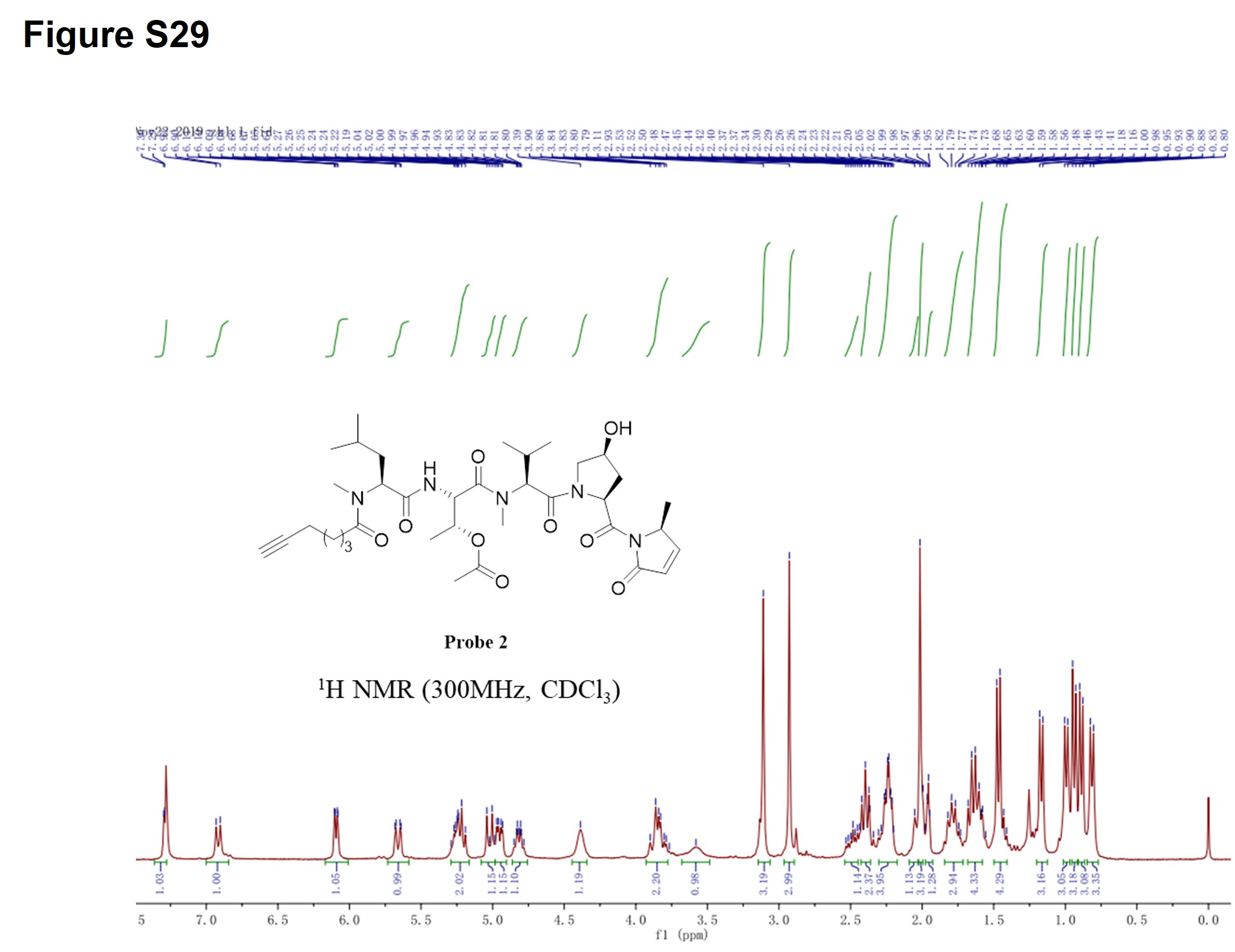
**

**Fig. S29** ^1^H NMR spectrum of compound **Probe 2**.

**
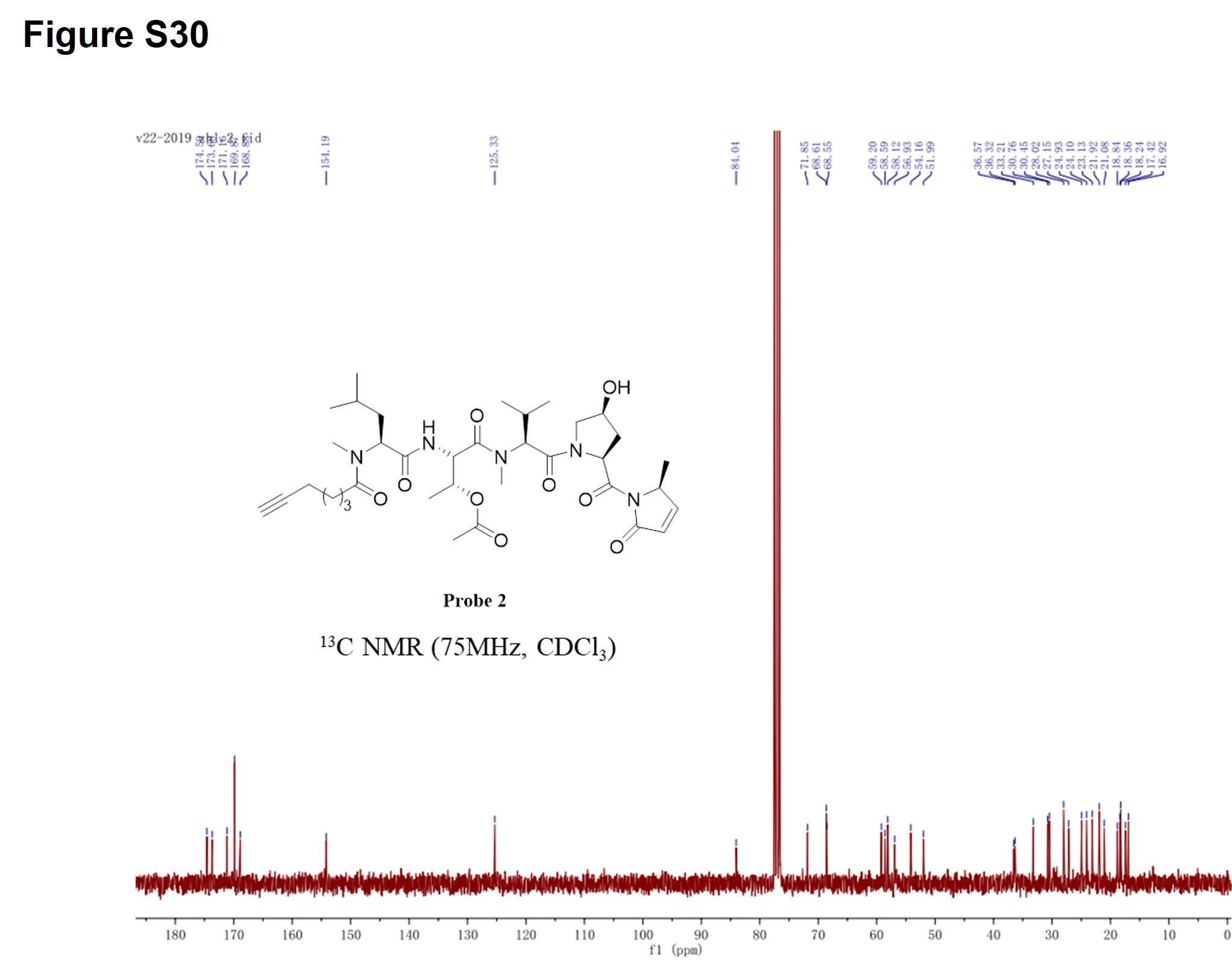
**

**Fig. S30** ^13^C NMR spectrum of compound **Probe 2**.

**
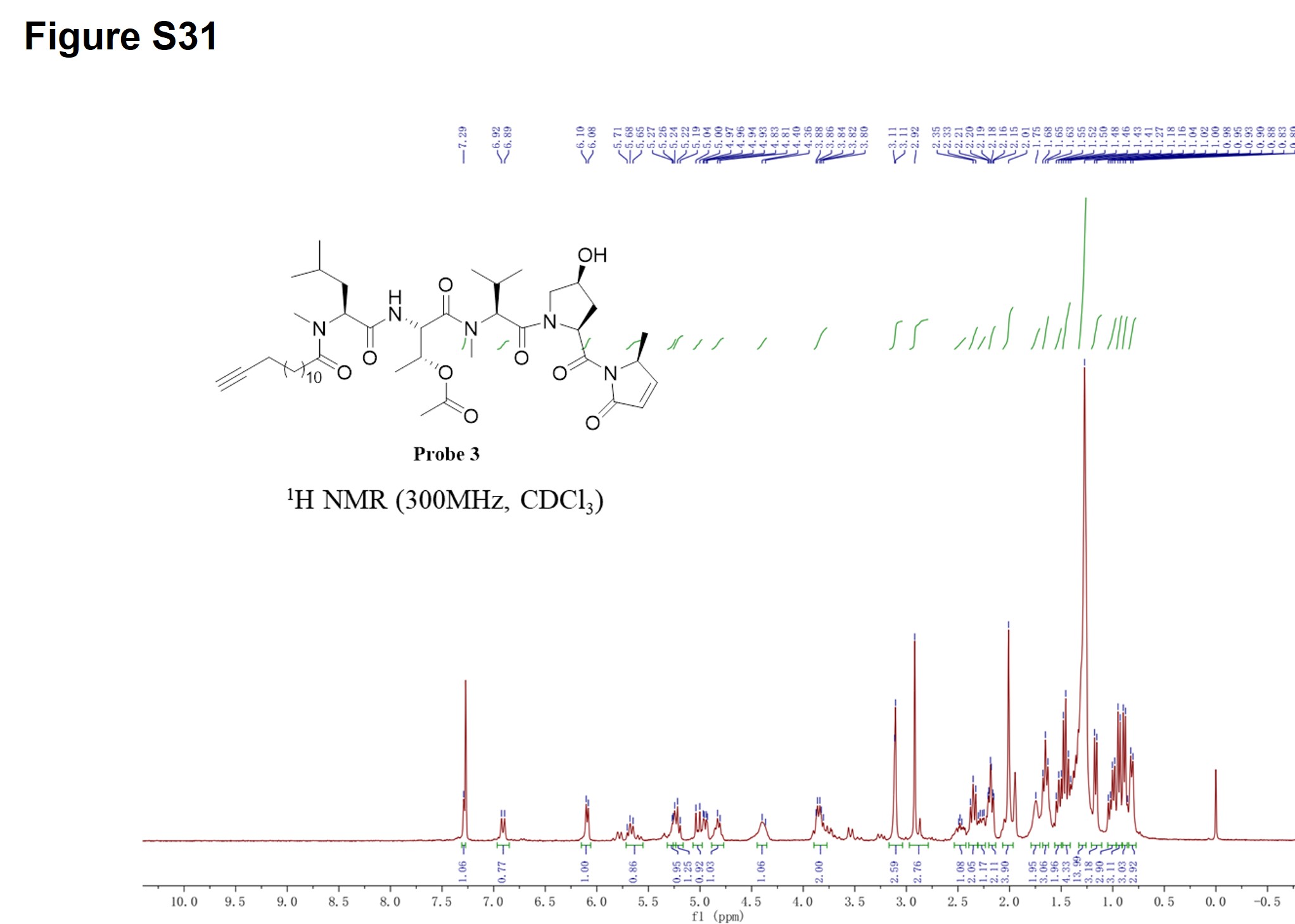
**

**Fig. S31** ^1^H NMR spectrum of compound **Probe 3**.

**
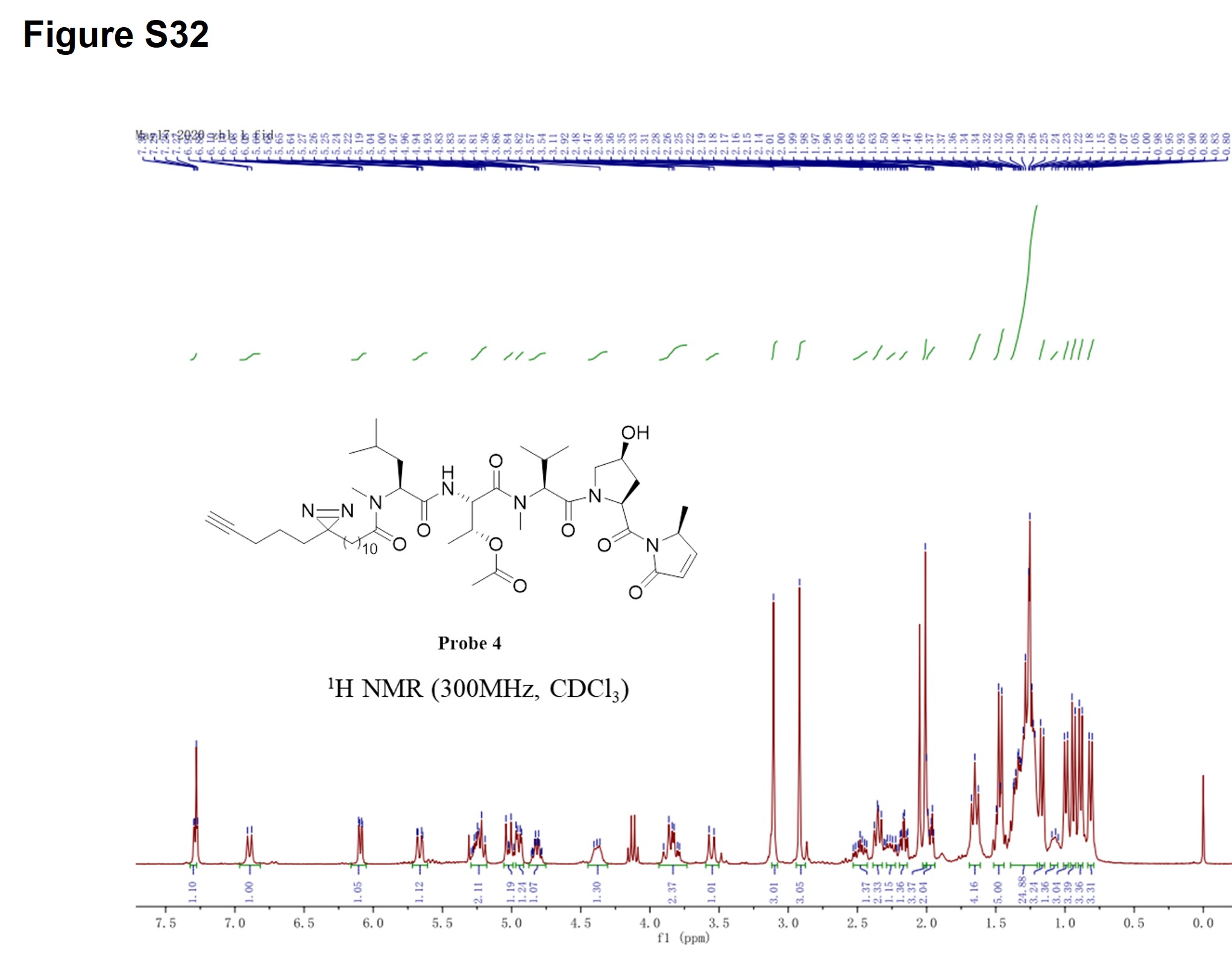
**

**Fig. S32** ^1^H NMR spectrum of compound **Probe 4**.

**
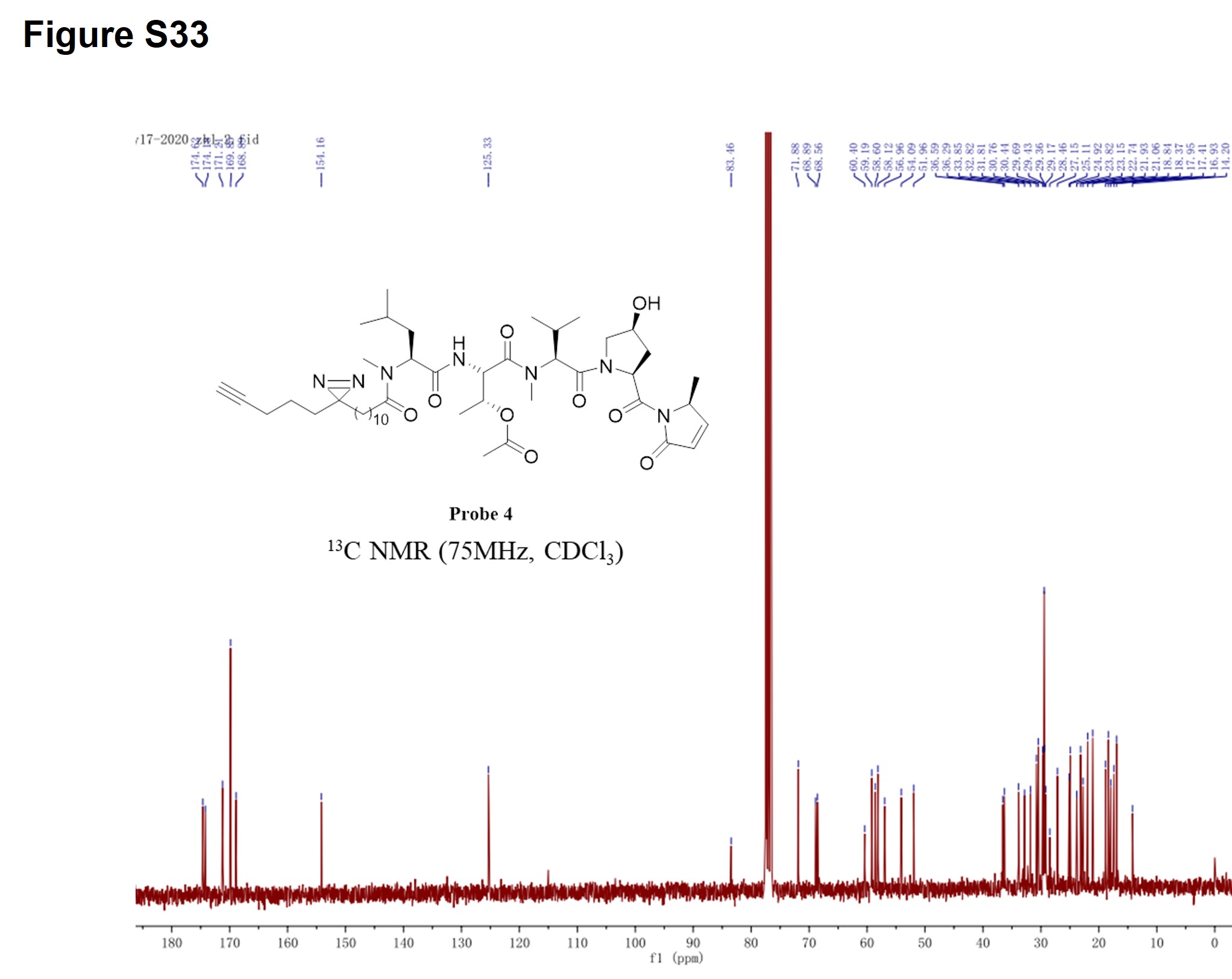
**

**Fig. S33** ^13^C NMR spectrum of compound **Probe 4**.

**
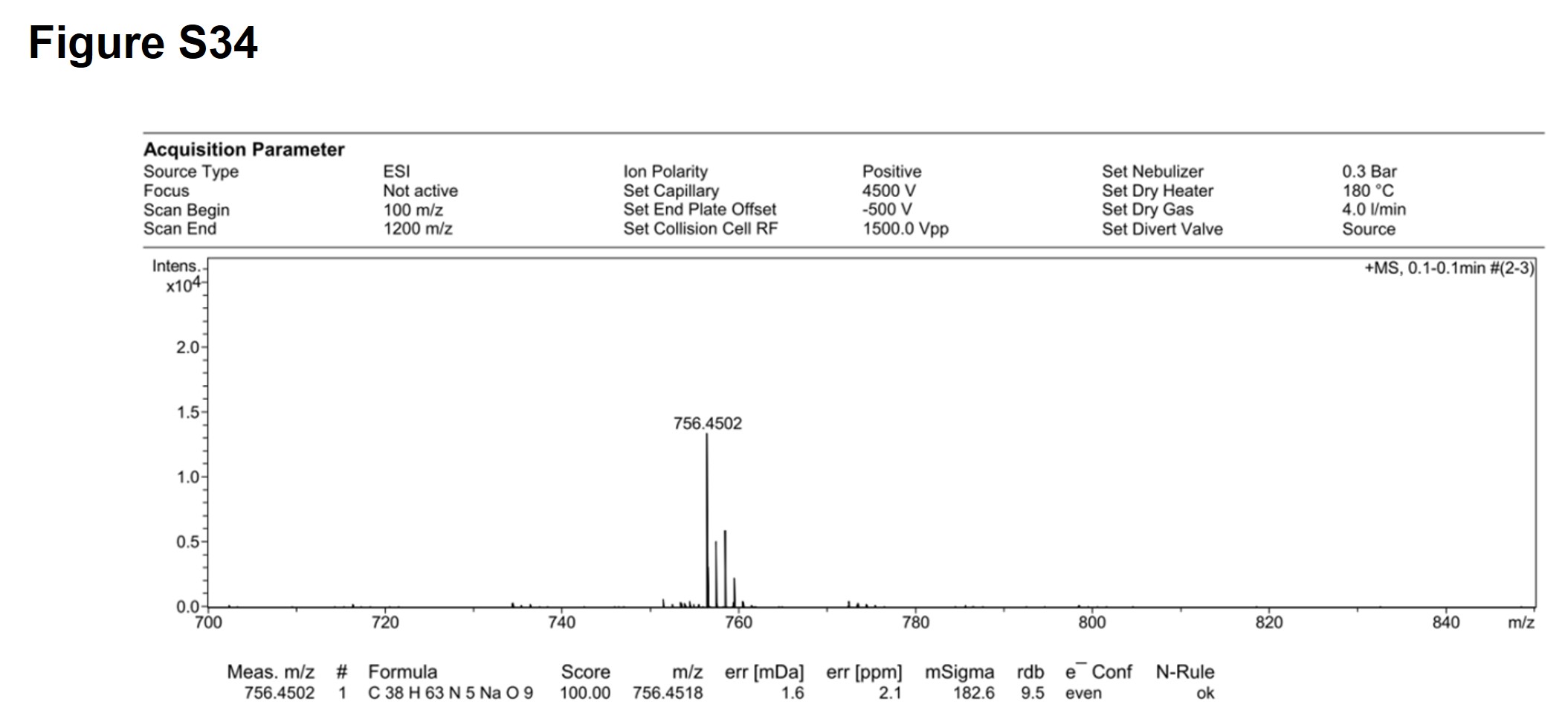
**

**Fig. S34** HRMS of **Microcolin H**.

**
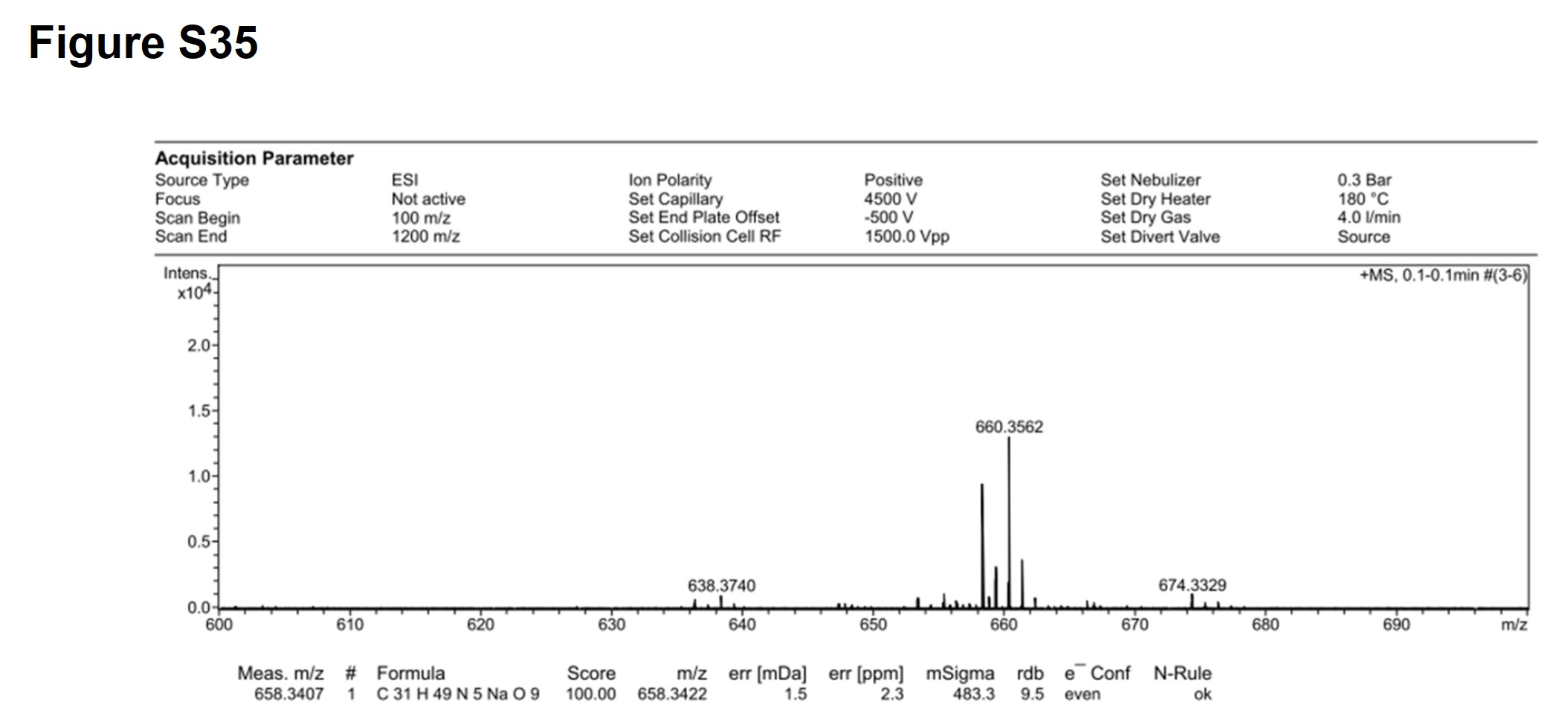
**

**Fig. S35** HRMS of compound **A-1**.

**
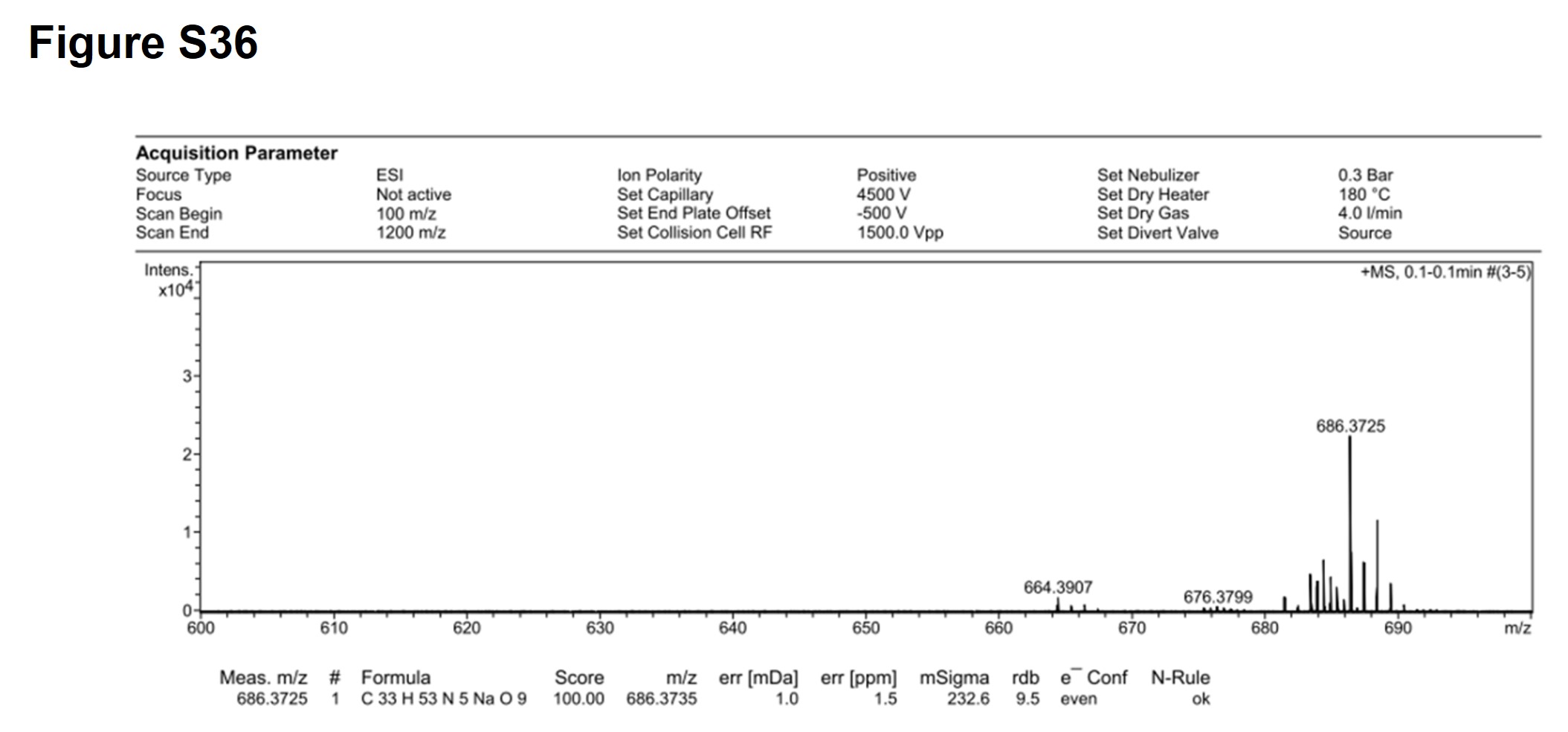
**

**Fig. S36** HRMS of compound **A-2**.

**
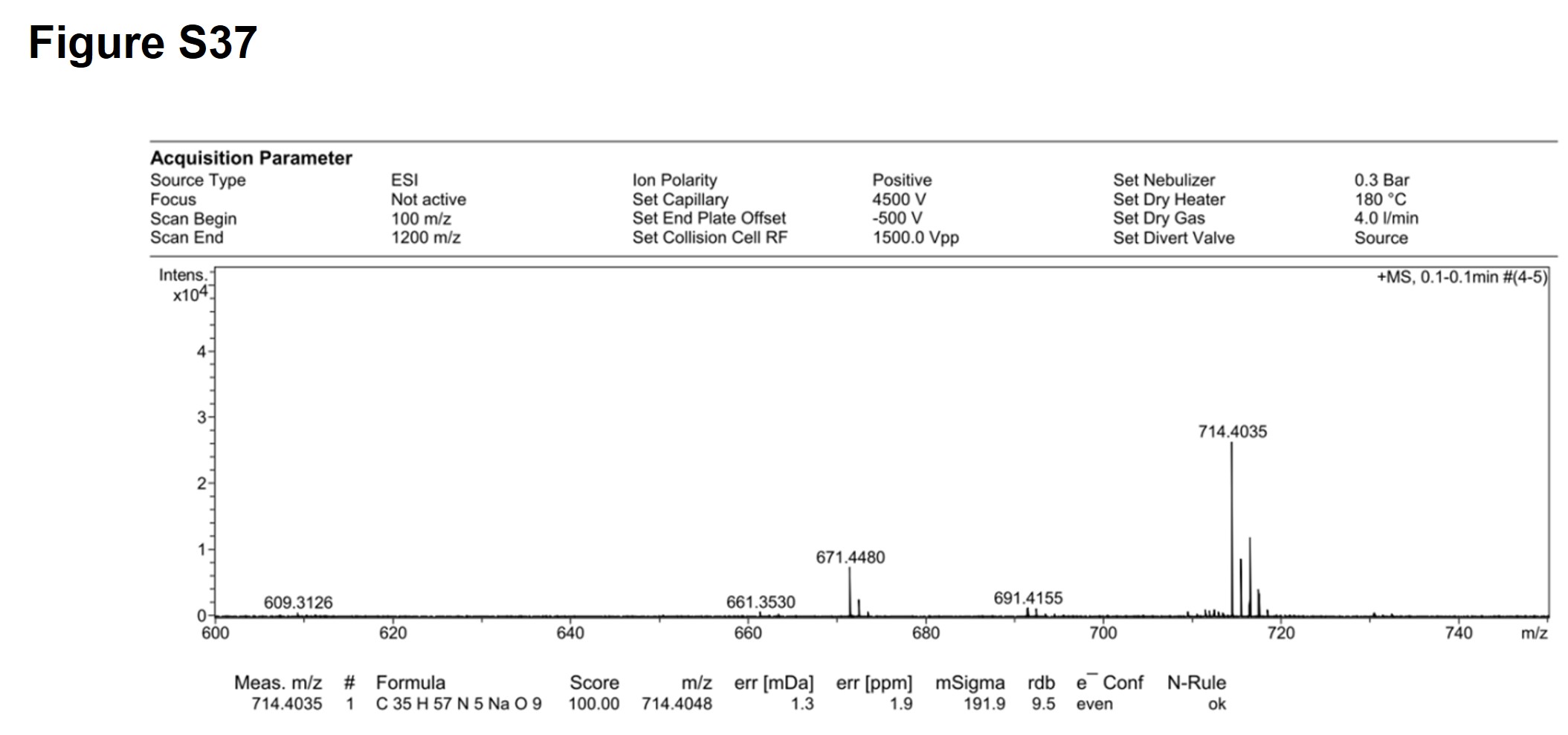
**

**Fig. S37** HRMS of compound **A-3**.

**
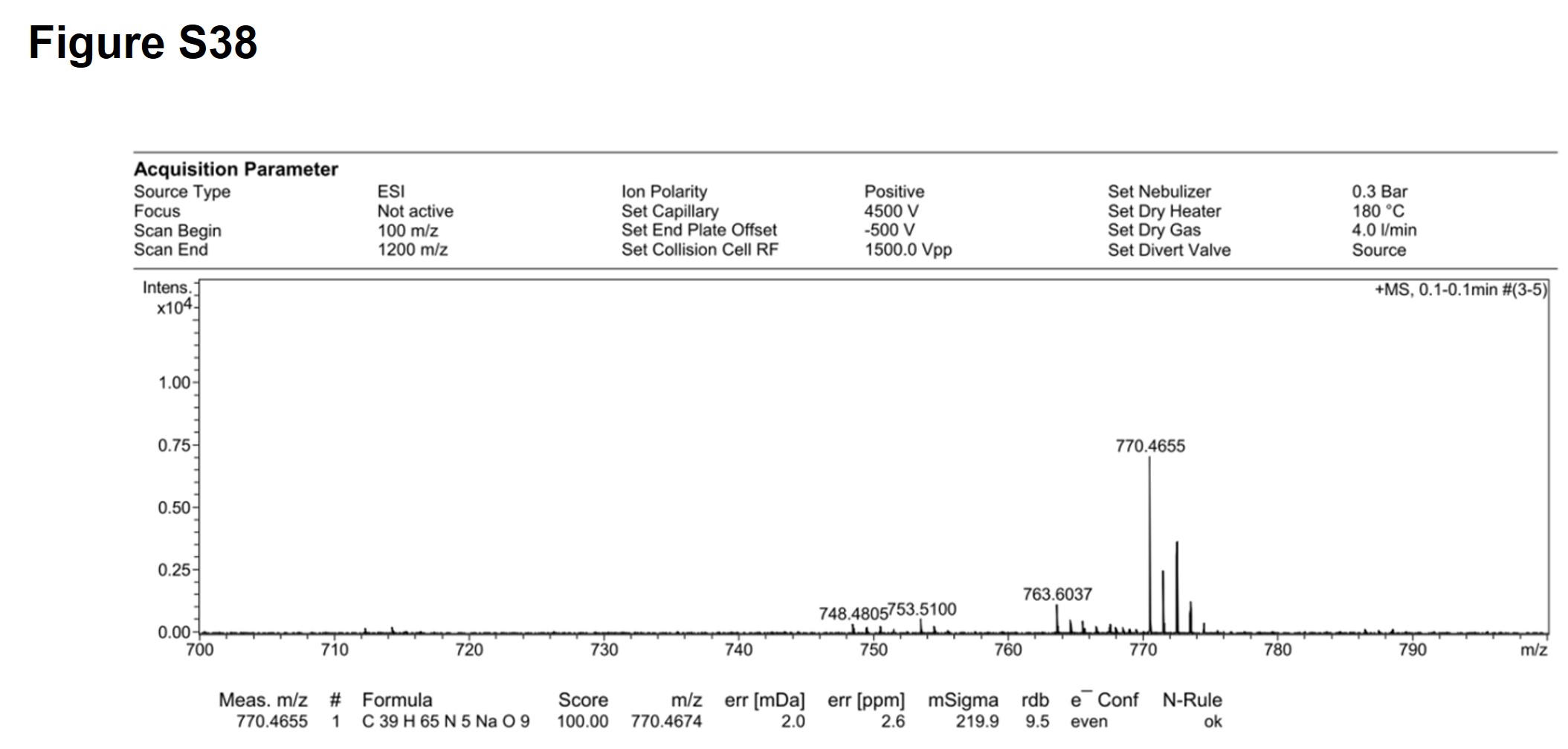
**

**Fig. S38** HRMS of compound **A-4**.

**
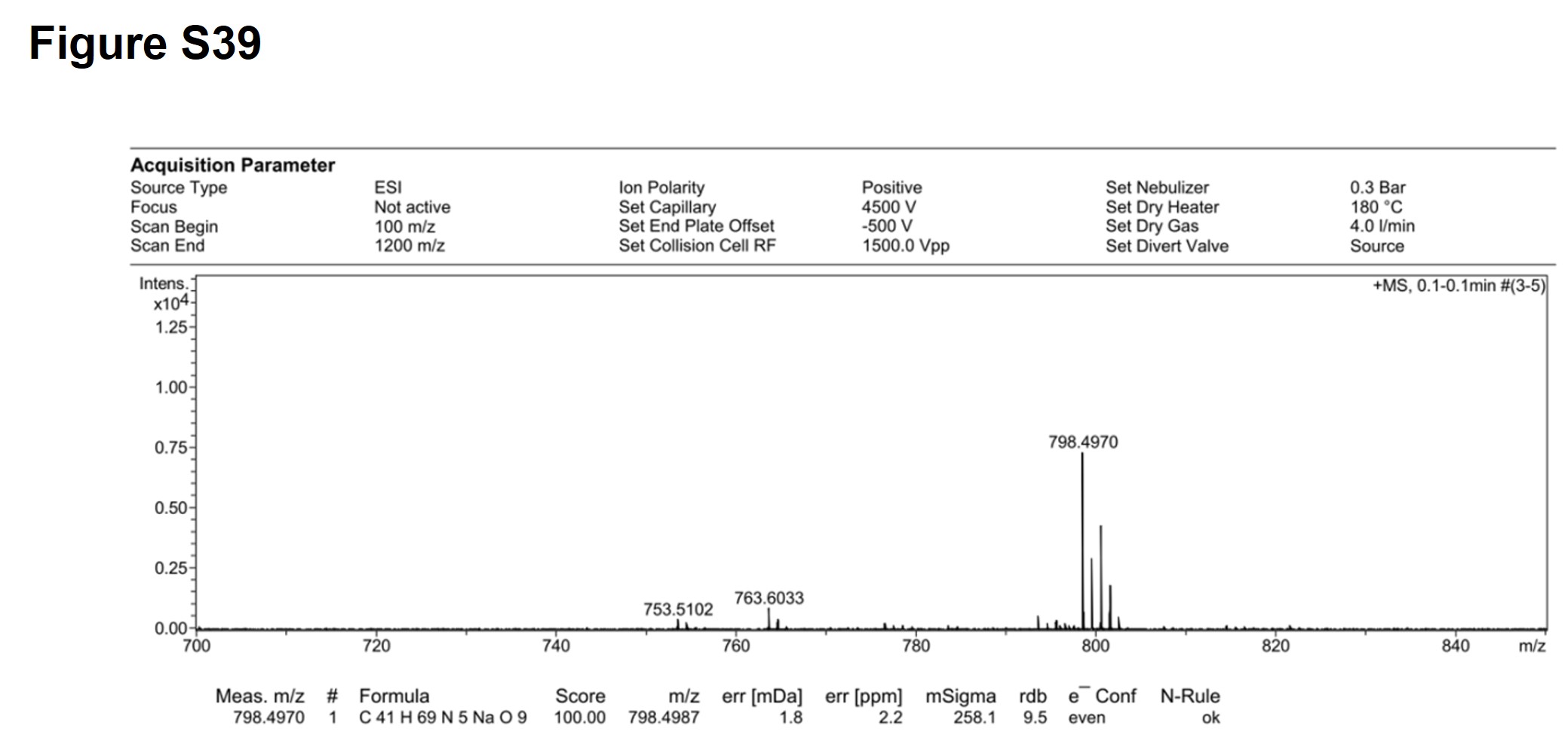
**

**Fig. S39** HRMS of compound **A-5**.

**
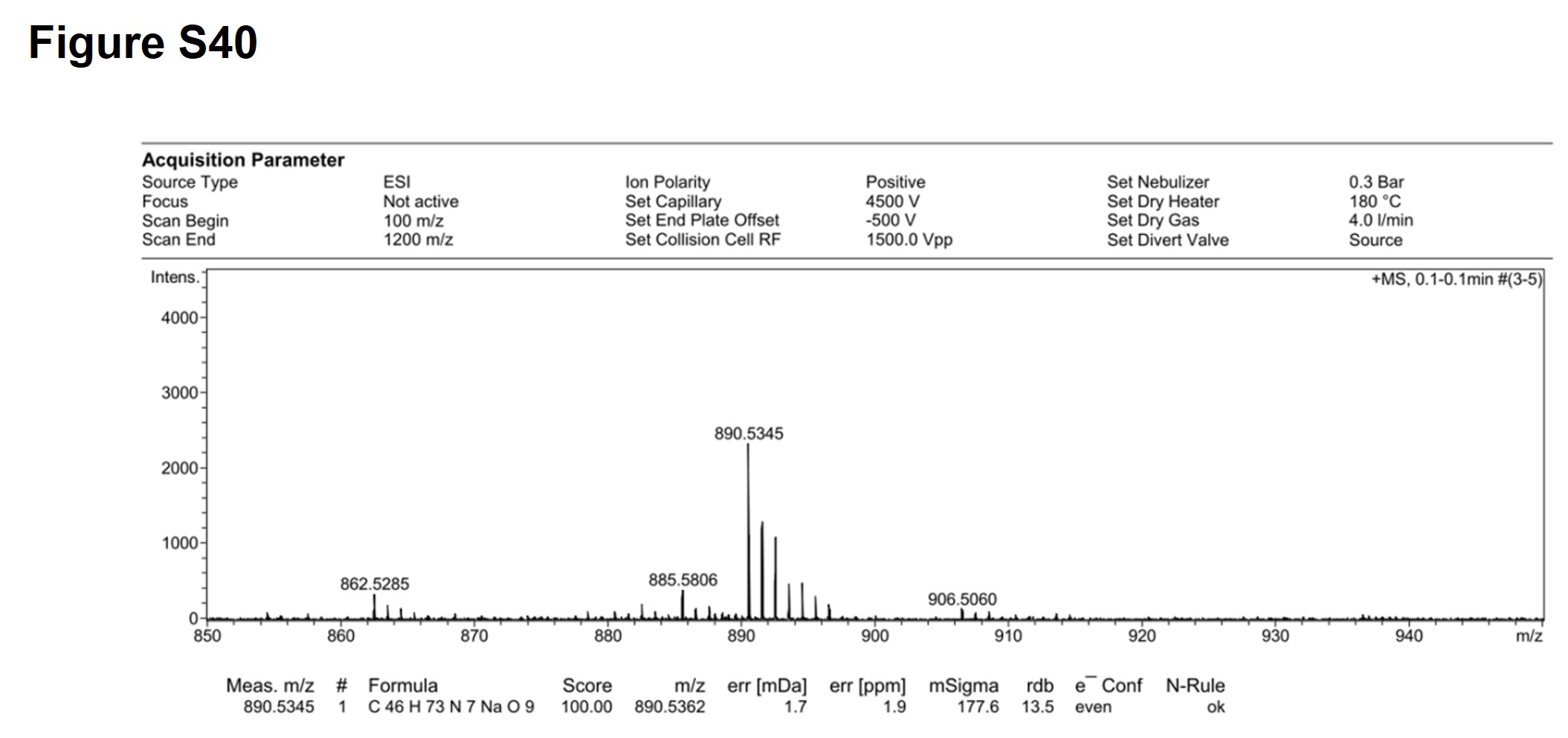
**

**Fig. S40** HRMS of compound **probe 4**.


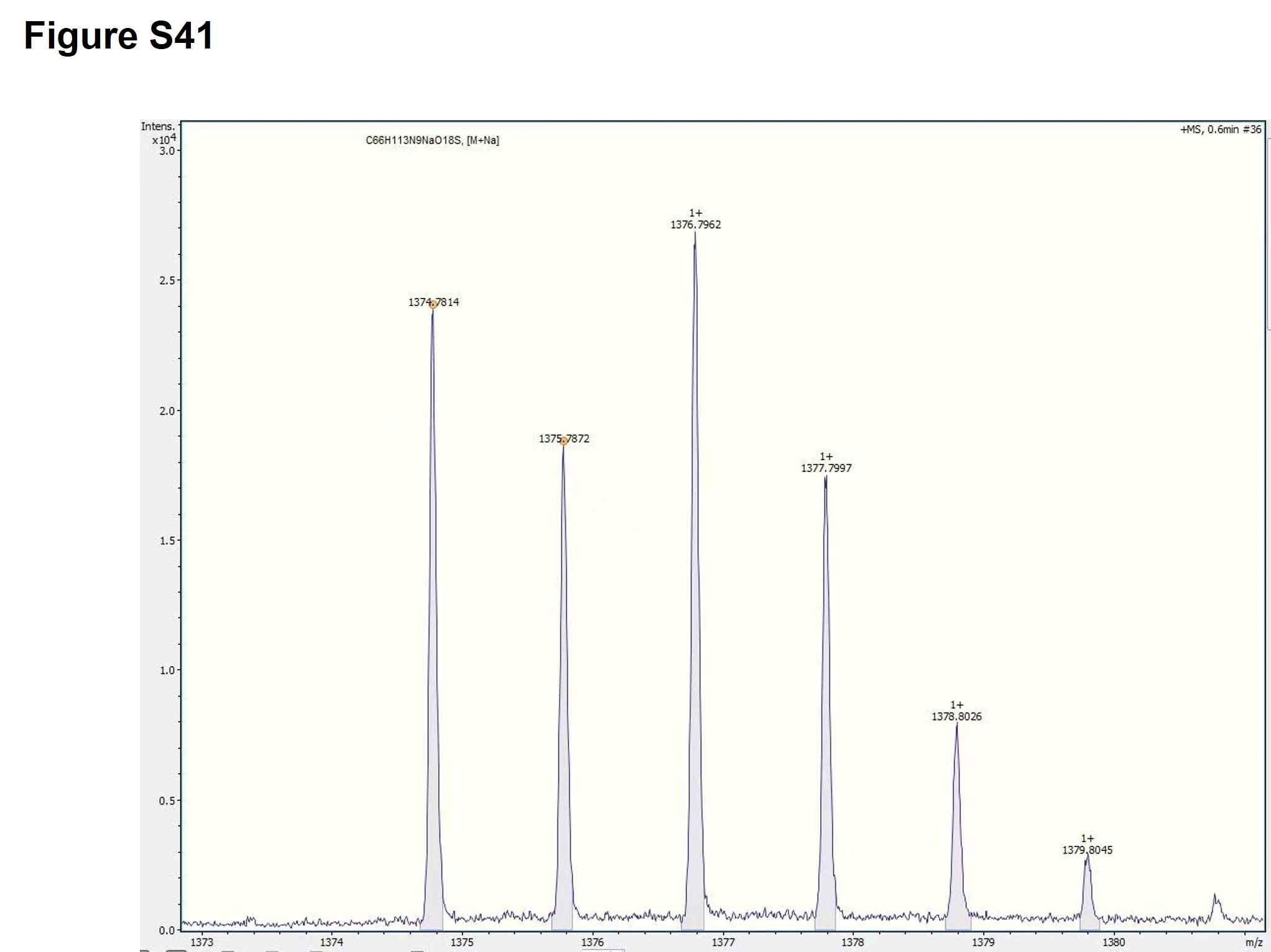


**Fig. S41** HRMS of compound **probe 5**.


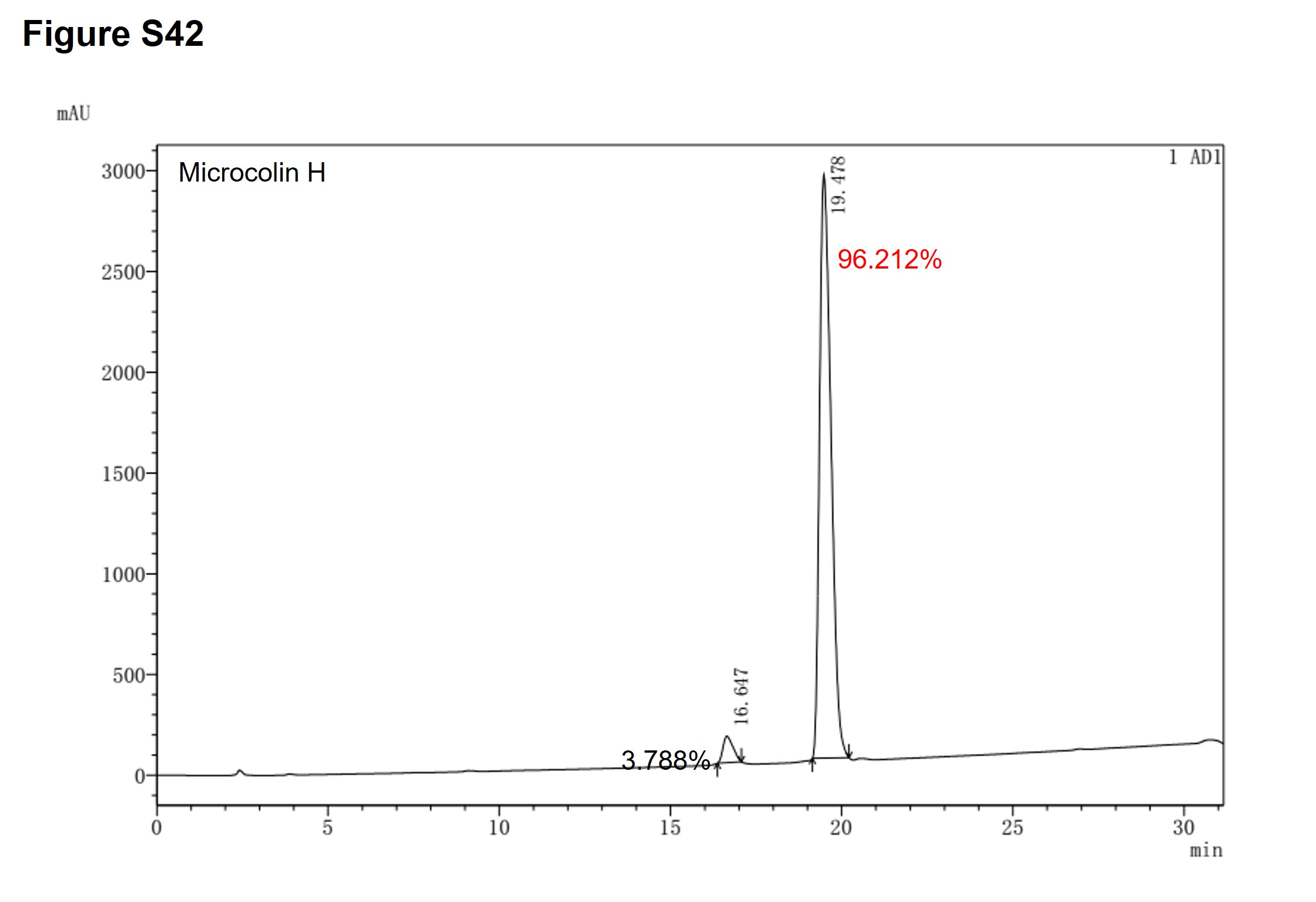


**Fig. S42** RP-HPLC purity analysis chromatogram of Microcolin H.


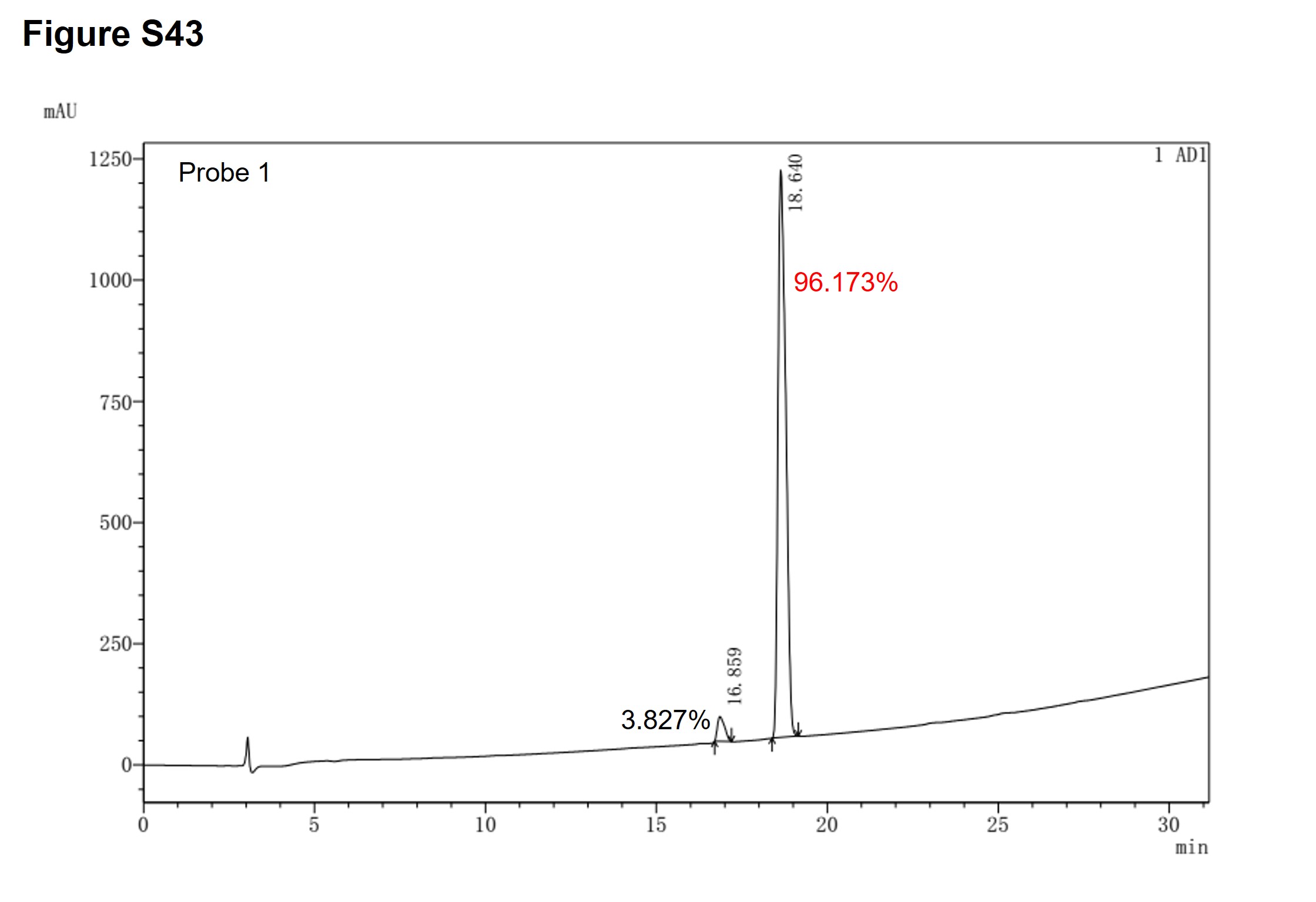


**Fig. S43** RP-HPLC purity analysis chromatogram of Probe 1.


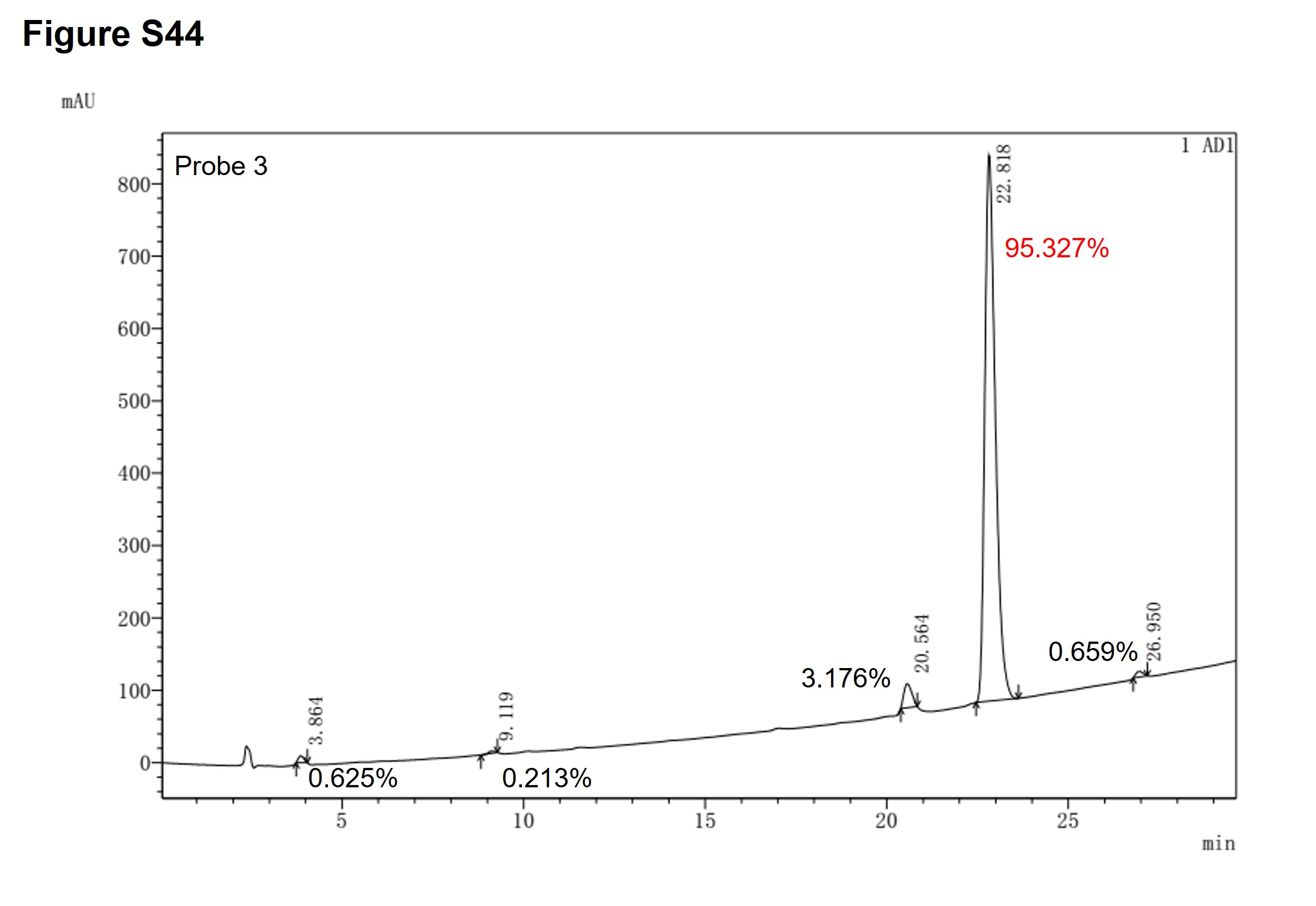


**Fig. S44** RP-HPLC purity analysis chromatogram of Probe 3.


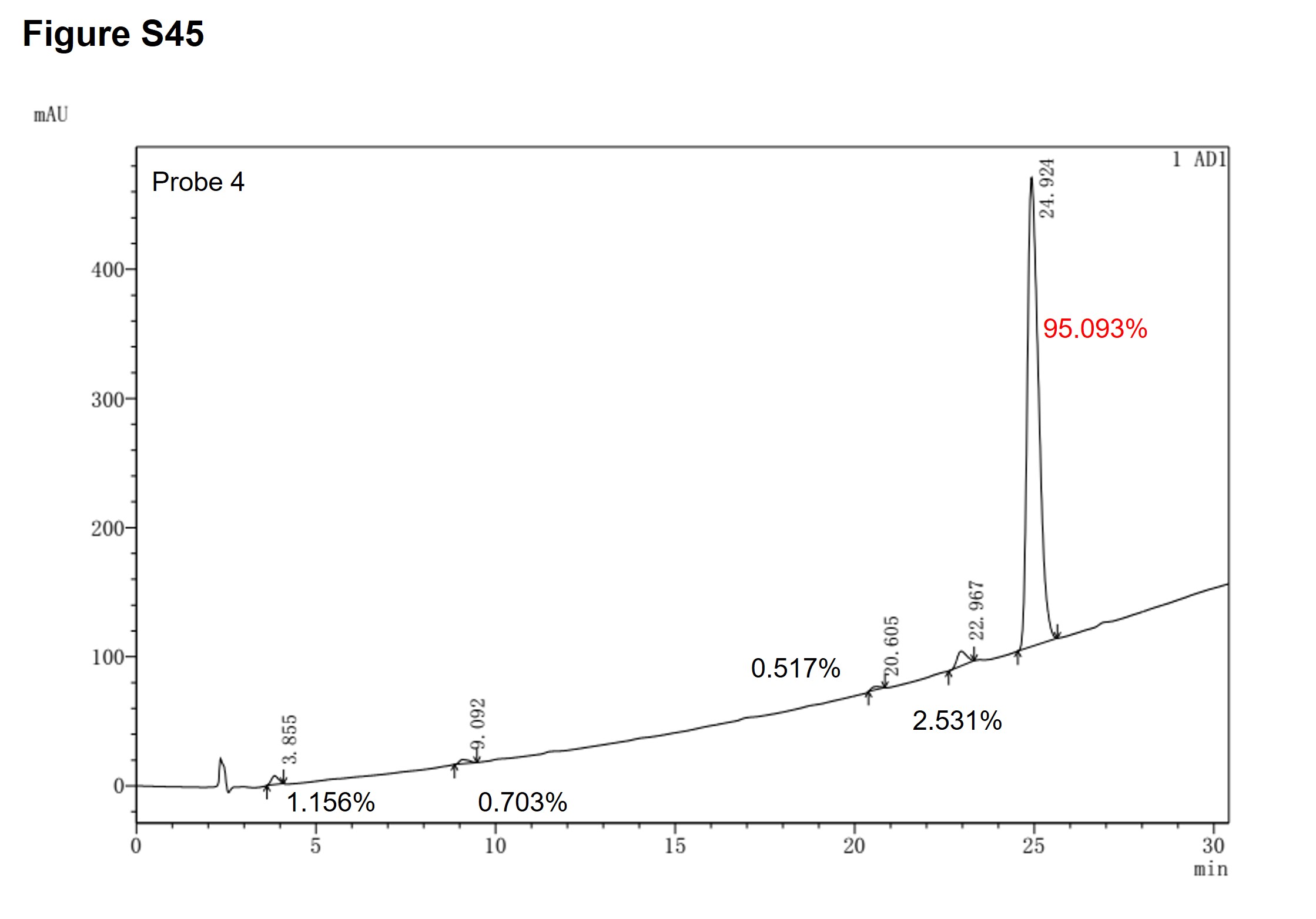


**Fig. S45** RP-HPLC purity analysis chromatogram of Probe 4.


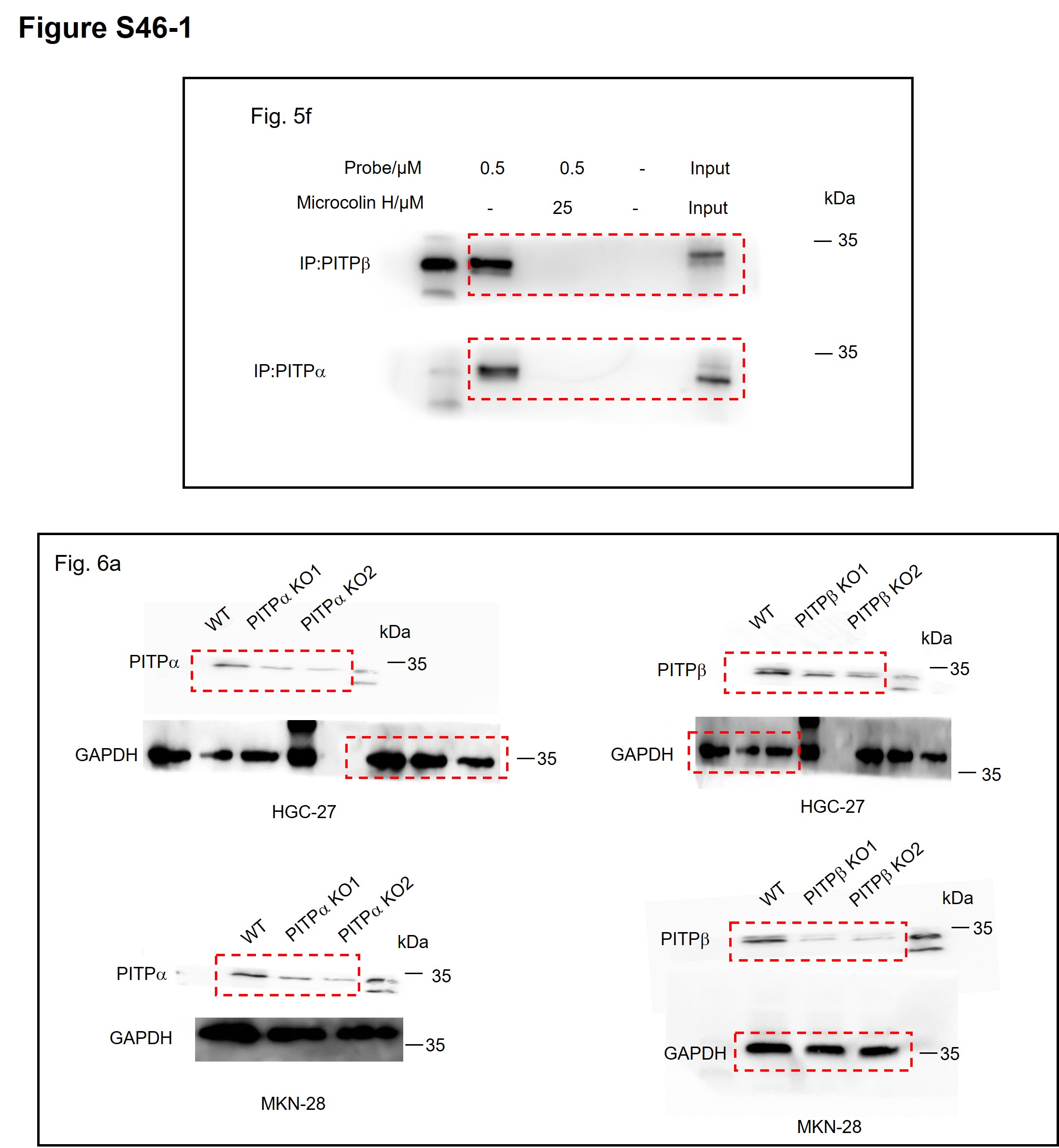


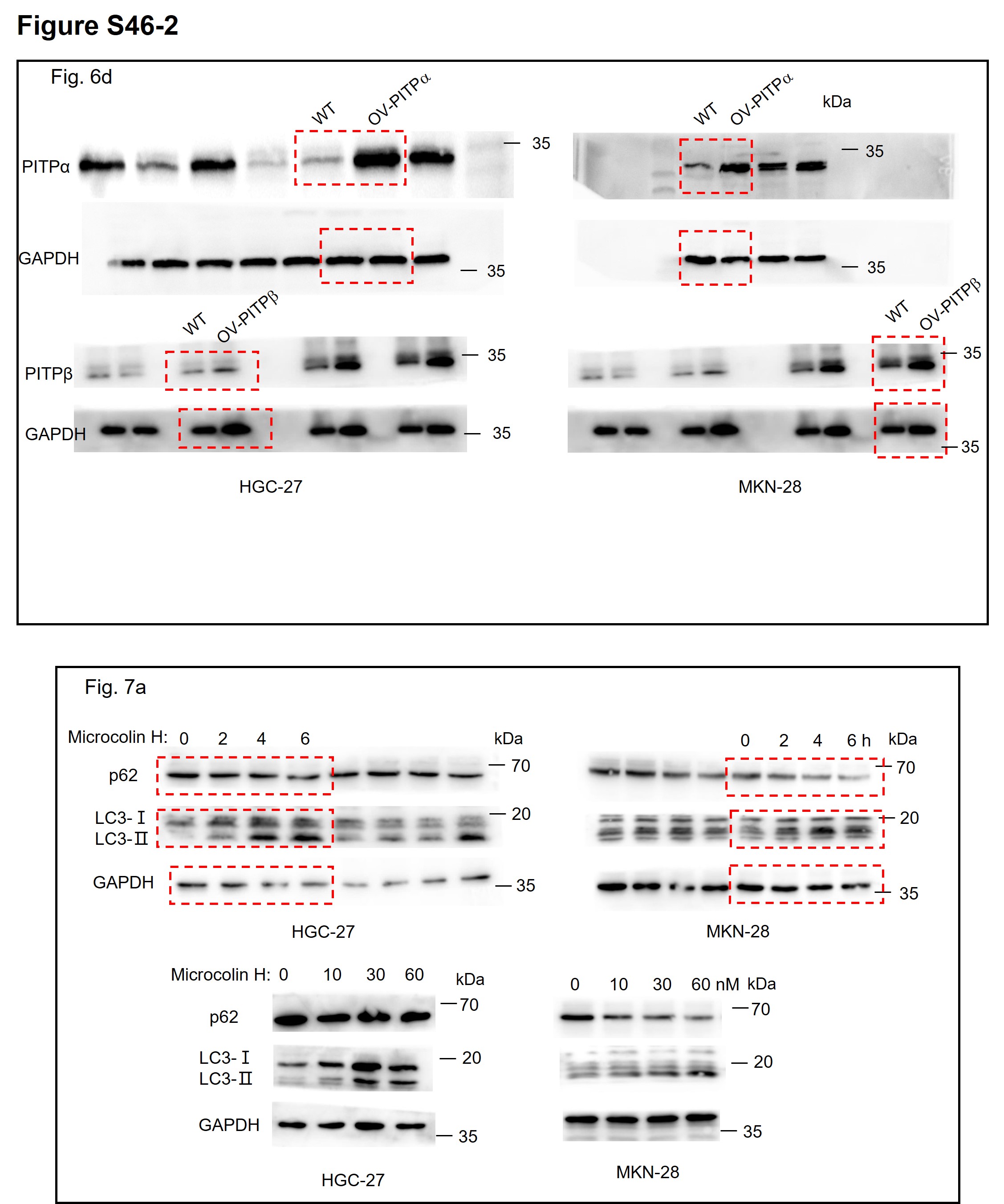


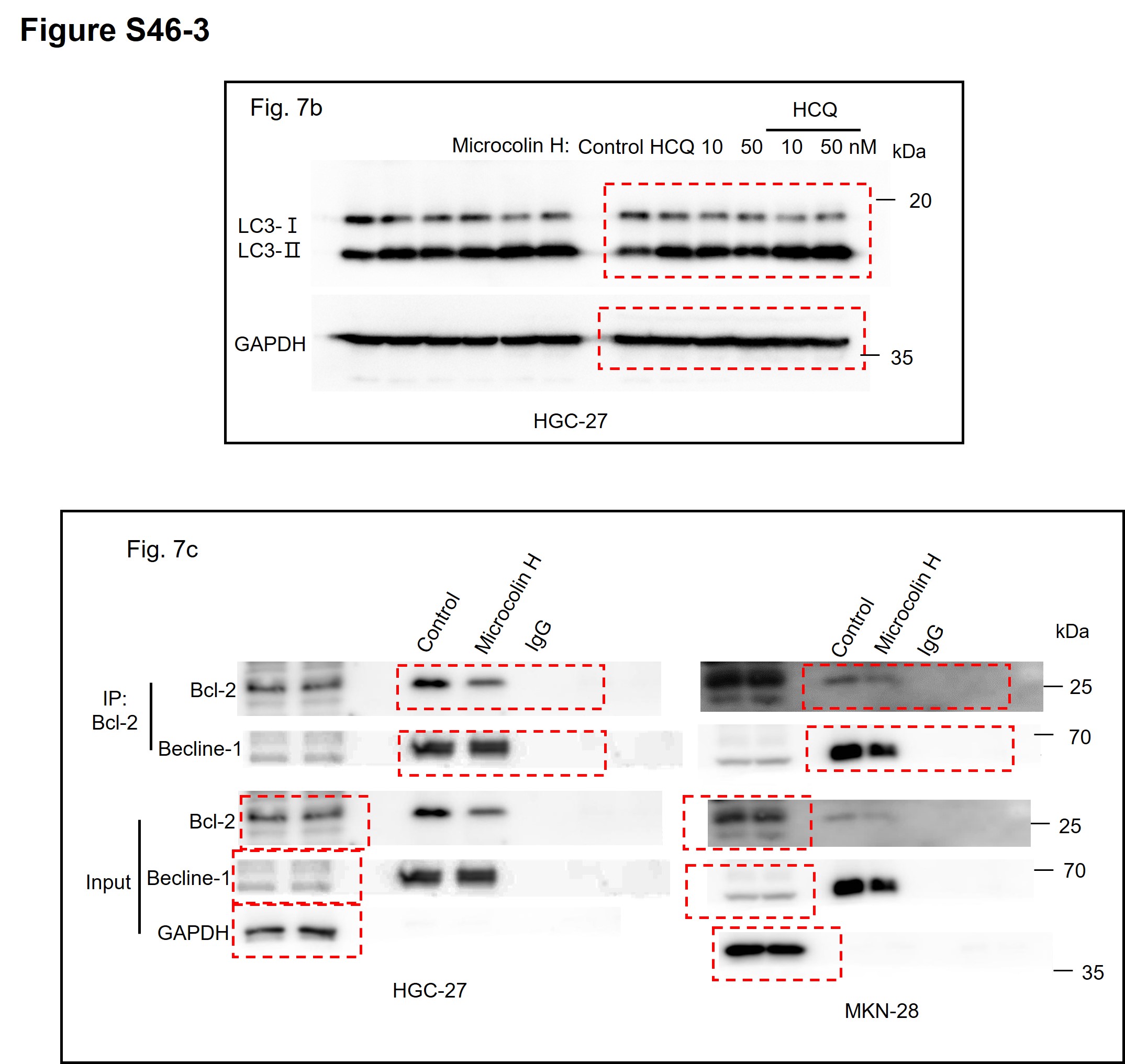


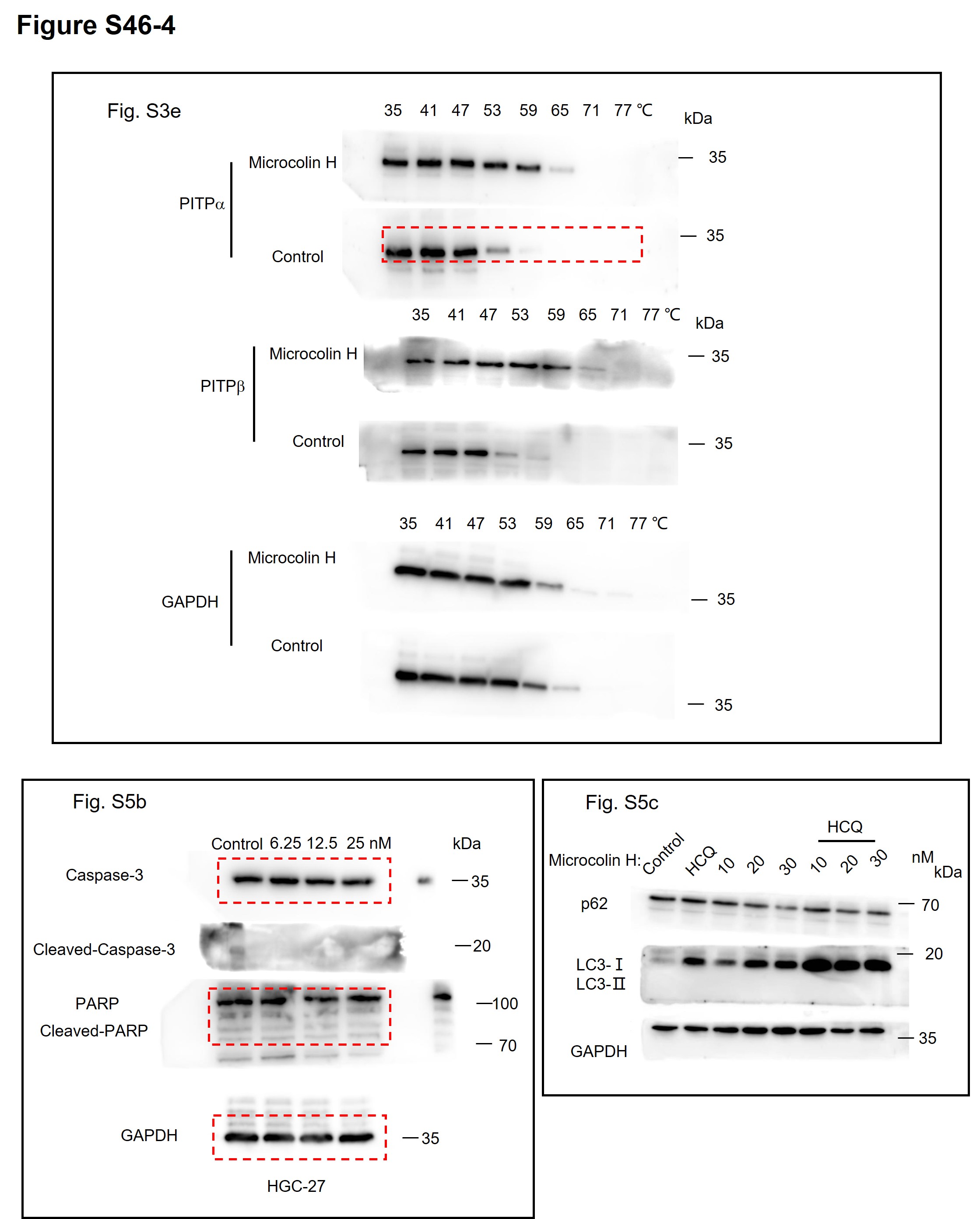


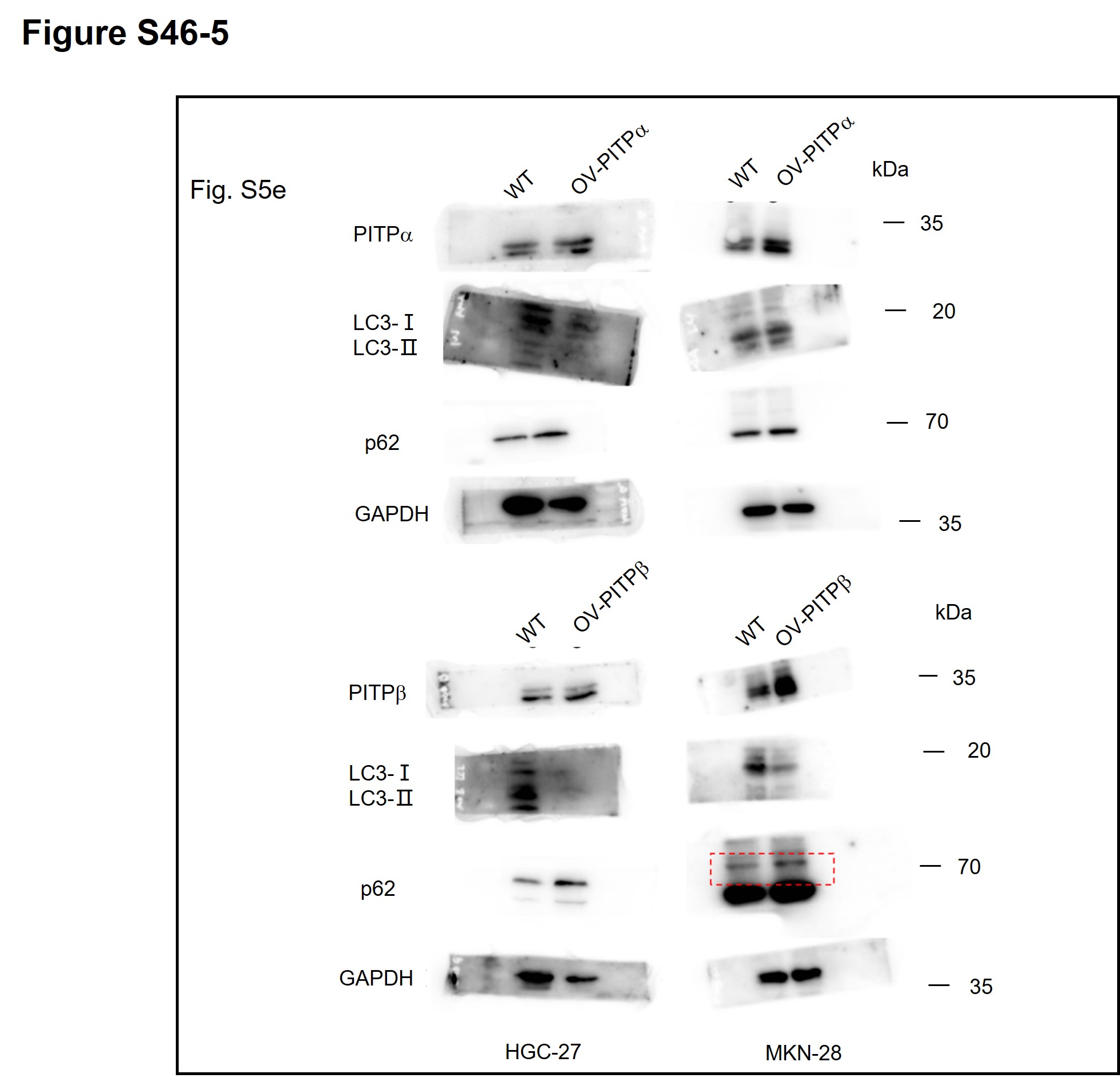


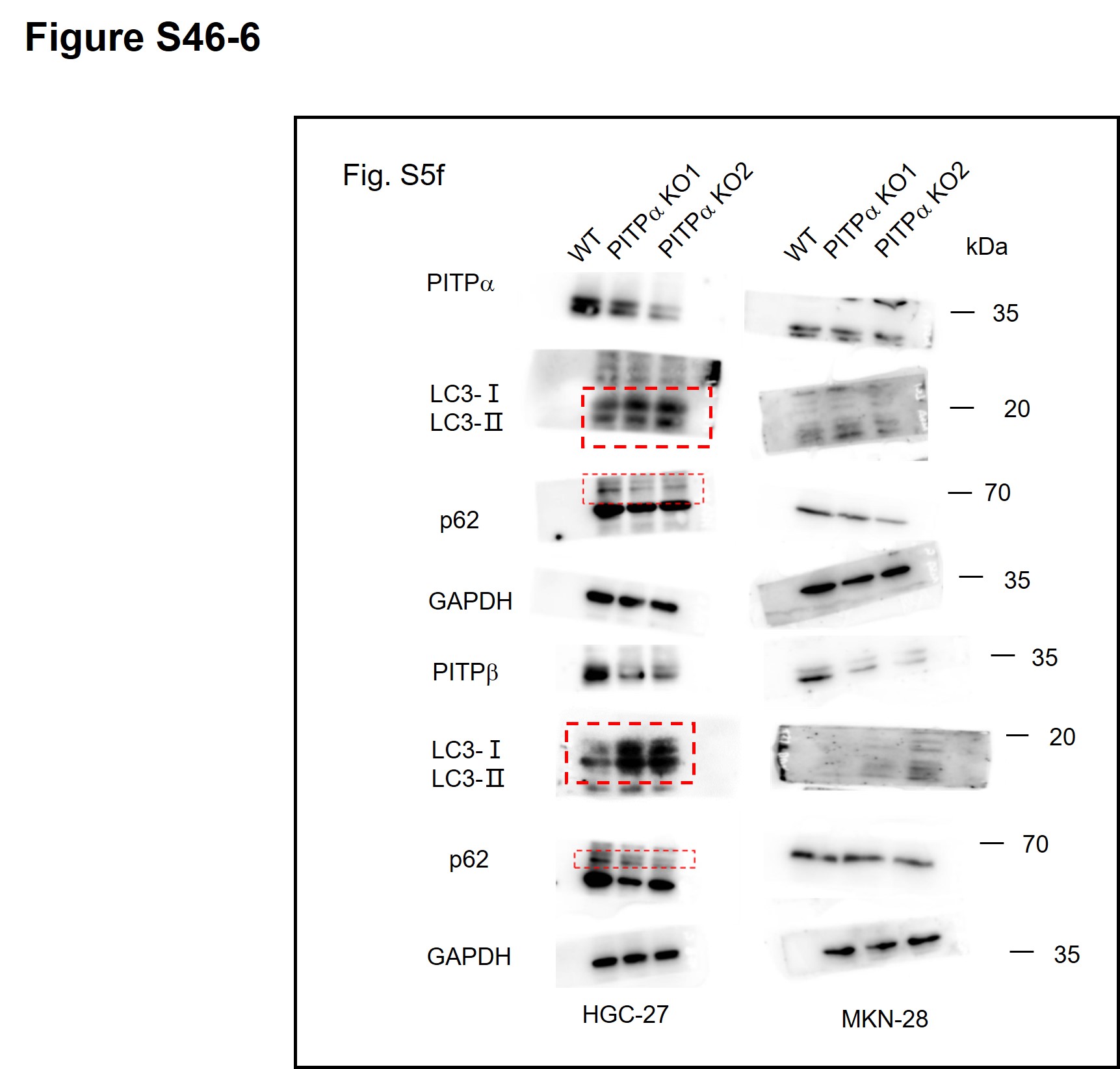


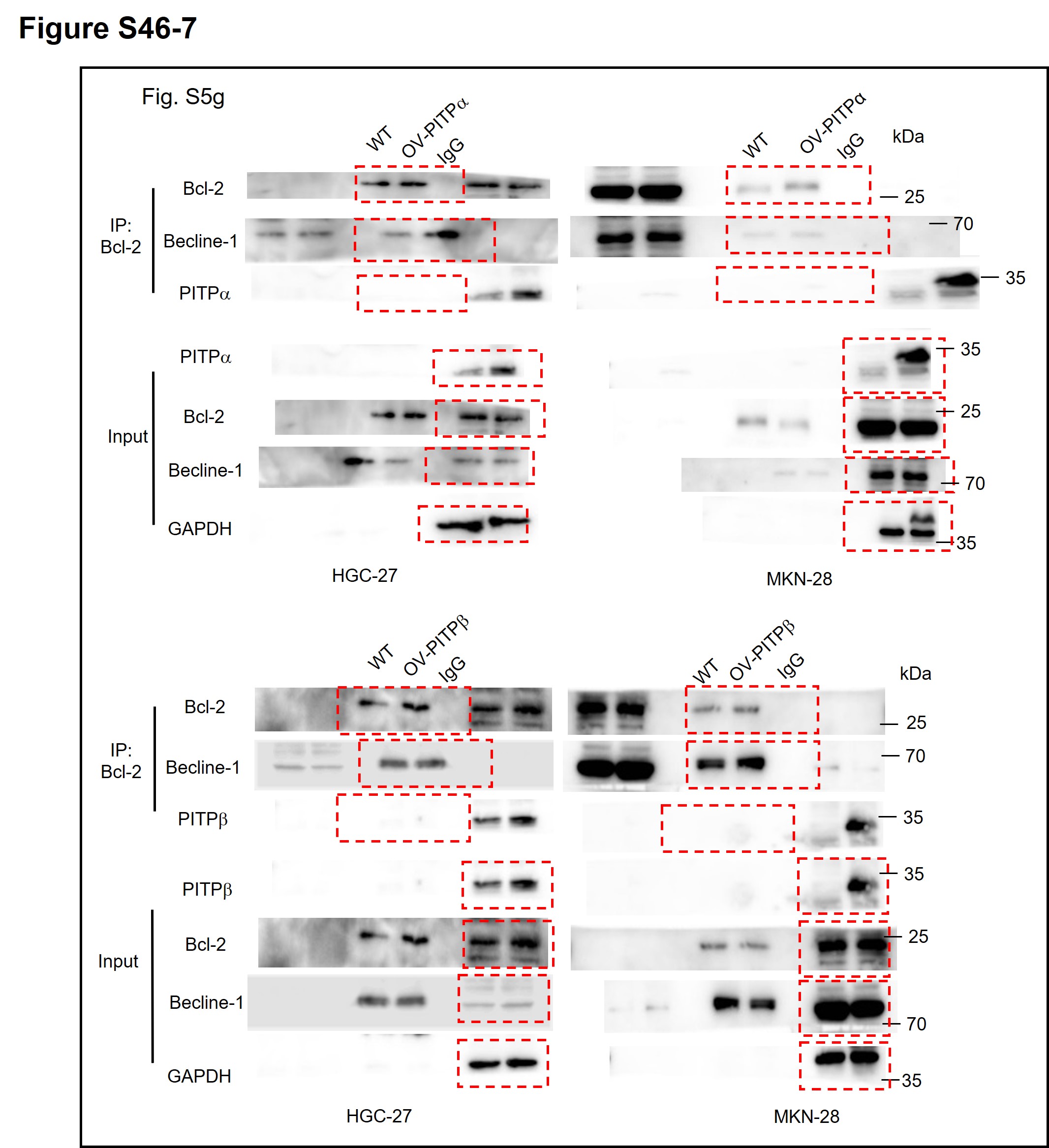


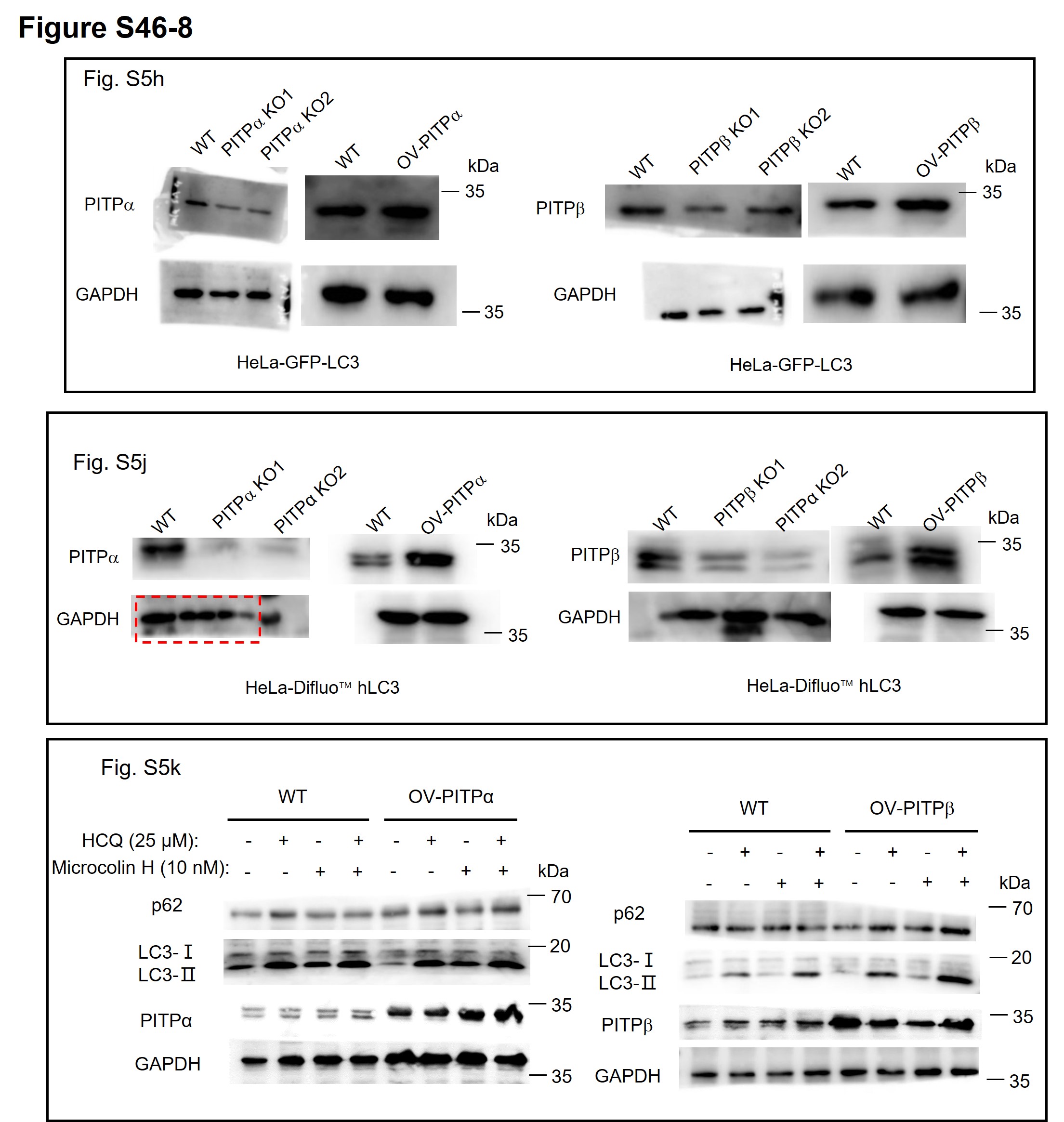


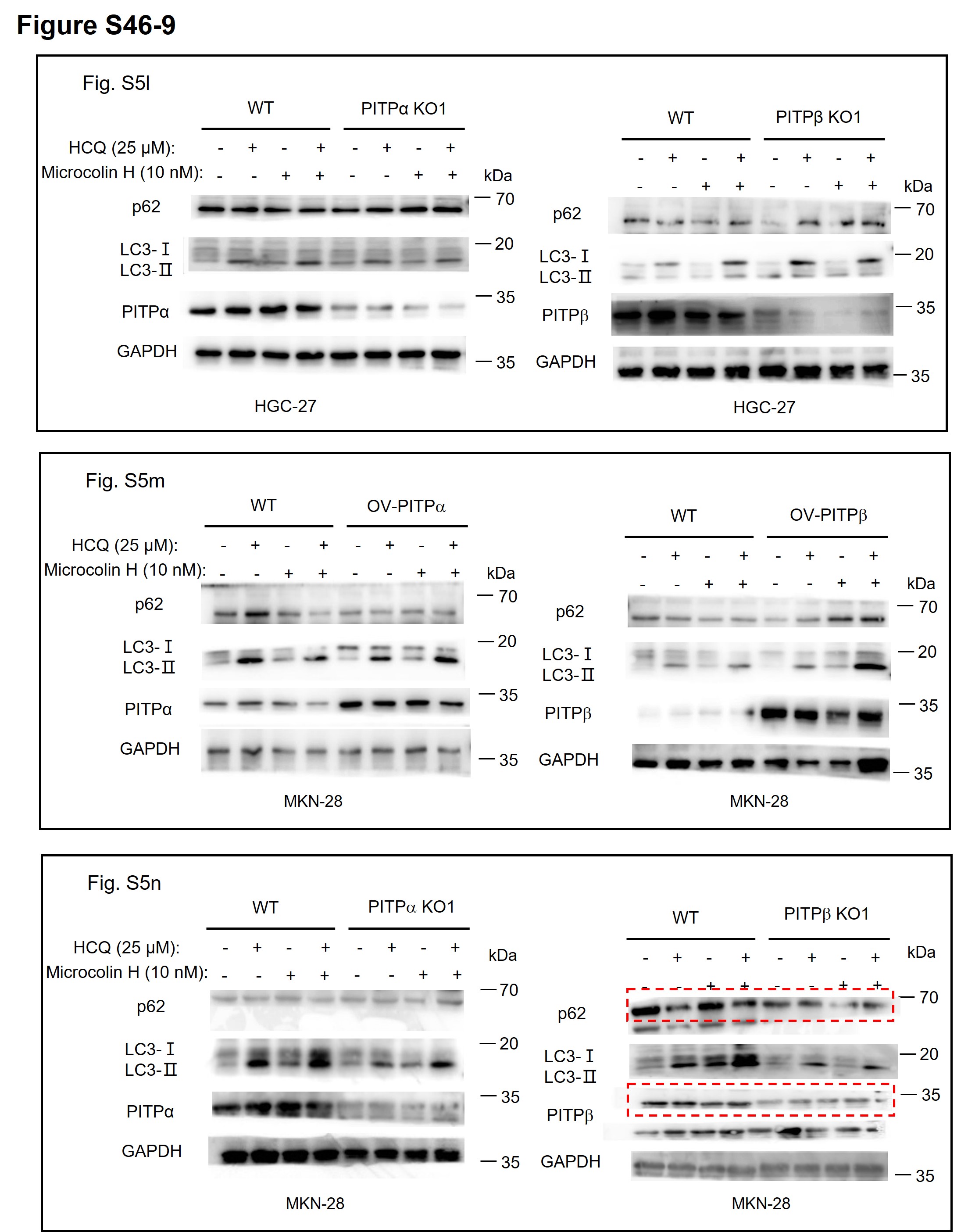


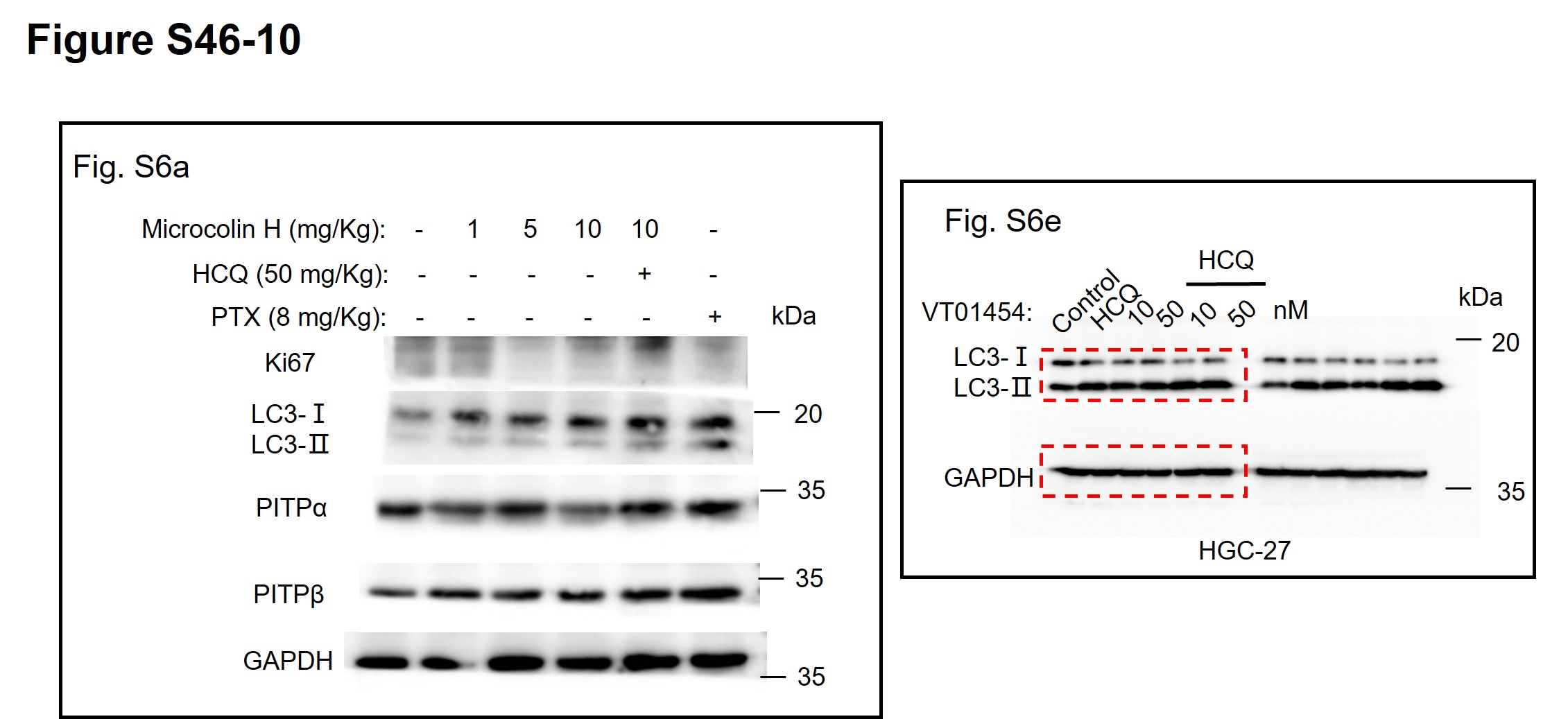


**Fig. S46** Original images of western blots

Supplementary Movies

**Movie. S1** Real-time cell imaging of DMSO treated HeLa-Difluo™ hLC3 cells.

**Movie. S2** Real-time cell imaging of Microcolin H treated HeLa-Difluo™ hLC3 cells.
